# Treatment satisfaction with injectable disease-modifying therapies in patients with relapsing-remitting multiple sclerosis (the STICK study)

**DOI:** 10.1371/journal.pone.0185766

**Published:** 2017-10-19

**Authors:** Oscar Fernández, Eduardo Duran, Teresa Ayuso, Luis Hernández, Inmaculada Bonaventura, Mireia Forner, on behalf of the STICK Study Investigators Group

**Affiliations:** 1 Institute of Biomedical Research (IBIMA), Regional University Hospital, Malaga, Spain; 2 Juan Ramon Jimenez-Infanta Elena Hospitals, Huelva, Spain; 3 Navarra Hospital, Pamplona, Spain; 4 León Hospital, León, Spain; 5 Mutua Terrassa, Terrasa, Spain; 6 Sanofi Genzyme, Barcelona, Spain; Instituto Cajal-CSIC, SPAIN

## Abstract

**Background:**

Treatment satisfaction in patients with relapsing-remitting multiple sclerosis (RRMS) may impact adherence and thus clinical outcomes. The objective of this study was to measure the satisfaction of patients with RRMS with injectable disease-modifying therapies (DMTs) and to evaluate the factors associated with treatment satisfaction.

**Material and methods:**

In this observational retrospective study conducted in the neurology departments of 35 hospitals throughout Spain, demographic data, disease characteristics, and information on treatment with injectable DMTs were collected at a single scheduled visit. Treatment satisfaction was assessed using the Treatment Satisfaction Questionnaire for Medication (TSQM), version 1.4. Patients also answered complementary questions about the factors that might affect treatment satisfaction. The data collected were analyzed descriptively. A regression model was used to explore the factors associated with treatment satisfaction.

**Results:**

The study included 445 patients (mean±SD age, 41±10.2 years; two-thirds women). The percentages treated with each DMT were Avonex 28.5%, Rebif 44 μg 24.5%, Copaxone 22.5%, Betaferon 13.0%, Rebif22 μg 8.3% and Extavia 3.1%. The mean±SD overall satisfaction according to the TSQM was 68.8±18.6 and the highest overall satisfaction was reported for Rebif 22 μg (72.4±20.3) and the lowest for Extavia (61.7±23.7). In the regression analysis, rehabilitation, interference with social life, pain on injection and number of MS treatments received were significantly associated with a decrease in overall TSMQ score. A small but significant negative correlation was found between EDSS scores and TSMQ scores (rho = –0.11, p = 0.02) and effectiveness (rho = –0.17, p<0.001). A perceived inconvenience of injections was reflected by the stated preference of 83% for once-daily oral treatment over other administration routes.

**Conclusions:**

Patients on stable injectable DMT therapy were reasonably satisfied with their treatment. Our results suggest that the main source of dissatisfaction with the current treatment is the inconvenience of the administration regimen.

## Introduction

In recent years, several new treatments for relapsing-remitting multiple sclerosis have come onto the market [1]. and further approvals are expected in the coming years [2]. Although many of the new treatments are subject to certain safety concerns [3], they have also been shown to be more effective than traditional therapies in patients with more advanced or more aggressive disease [1]. Patients with aggressive disease may be more prepared to accept the risks associated with these treatments. In contrast, the risks may appear less acceptable to patients with a mild disease course. Indeed, injectable disease-modifying therapies (DMTs) remain the mainstay of treatment in patients with mild stable disease [4]. Extensive post-marketing experience is available for these agents (interferon beta 1b was first approved by the FDA in 1993) and they have a well-defined adverse effect profile, and the complications can generally be readily managed [5].

Non-adherence has been associated with increased rates of relapse [6] and greater health resource utilization [6]. One factor that may impact adherence is treatment satisfaction [7]. Injectable DMTs can, for example, be inconvenient [8]. The need for regular injections can affect the acceptance of treatment and adherence to therapy [9], with reported non-adherence rates ranging from 21% to over 45% [10]. The problem is further compounded by the fact that these agents do not directly treat the symptoms and the course of MS is unpredictable [11]. The benefits of treatment therefore might not be readily apparent to patients, and this may demotivate patients to adhere to treatment [12]. An understanding of the factors that affect patient treatment satisfaction could improve adherence and thus lead to better clinical outcomes.

The aim of this observational, retrospective study was to measure the satisfaction of patients with RRMS with injectable DMTs and to evaluate the factors associated with treatment satisfaction.

## Material and methods

**Fig 1:**
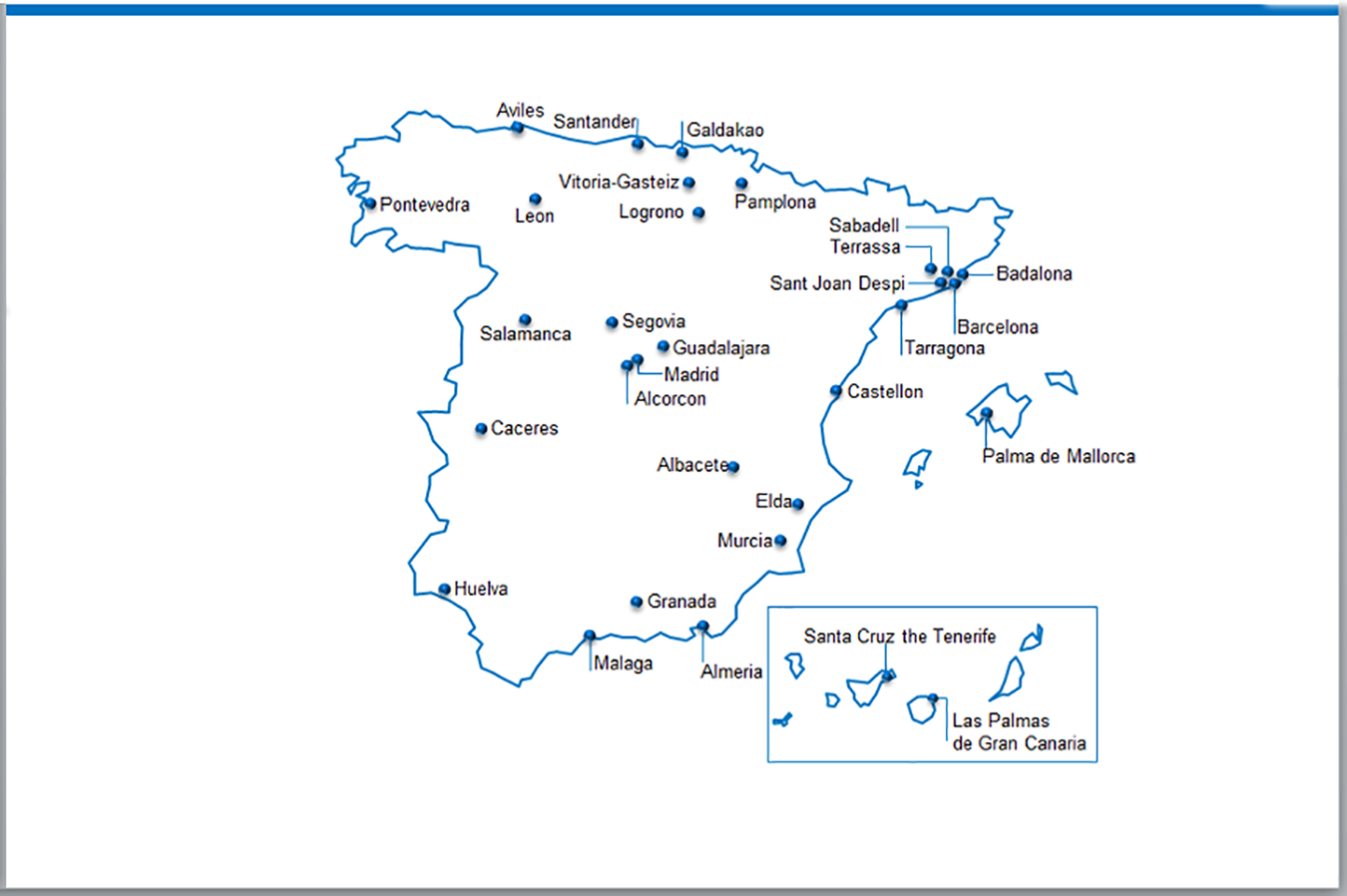
Geographic distribution of participating hospital throughout Spain (mainland and islands).

The STICK study is an observational retrospective study conducted between October 2014 and March 2015 at the Neurology departments of 35 hospitals throughout the entire geographic area of Spain (see Fig 1 for the distribution of investigators throughout the country and the appendix for a list of participating hospitals). Prior to initiation, the study was approved by the Comité de Ética de la Investigación Provincial de Málaga. Servicio Andaluz de Salud. Consejería de Igualdad, Salud y Políticas Sociales CEIC de Hosp Carlos Haya de Málaga and corresponding ethics committees of the participating centers and all patients provided informed consent in writing prior to enrolment.

At a scheduled visit to their neurologist, consecutive patients with RRMS (according to the 2005 revision of the McDonald criteria[13]) were assessed for eligibility. Only 28 eligible patients (approximately 6%) declined to participate for diverse reasons and therefore were not recruited in the study. To be included, patients had to be over 18 years of age and have been receiving an approved injectable disease-modifying therapy (DMTs) for 6 months or more. The protocol also permitted the enrollment of patients with clinically isolated syndrome if they had been on stable injectable DMT for 6 months. In Spain, the approved DMTs at the time of the study were interferon beta-1a intramuscular (IFNB-1a IM, AVONEX), IFNB-1a subcutaneous (IFNB-1a SC, REBIF) 44 μg or 22 μg, IFNB-1b SC (EXTAVIA®), IFNB-1b SC (BETAFERON®), and glatiramer acetate SC (COPAXONE®). Patients participating in another clinical trial and also those considered unable to complete the questionnaires described below were excluded.

Eligible patients who agreed to participate in the study signed an informed consent form and the demographic data as well as expanded disability status scores (EDSS), number of relapses in the previous year, and medication details were recorded on the electronic case report form. The following questionnaires used in the study were then administered:

- The Treatment Satisfaction Questionnaire for Medication (TSQM) version 1.4 was used to assess the patients’ treatment satisfaction. This questionnaire comprises 14 questions covering 4 domains for treatment satisfaction: TSQM effectiveness (questions 1–3), side effects (questions 4–8), convenience (questions 9–11), and global satisfaction (questions 12–14) [14]. Each scores on each domain ranges from 0 (extremely dissatisfied) to 100 (extremely satisfied). The questionnaire has been shown to be valid and robust [15]. The TSMQ can be applied in a range of chronic diseases, and recently, it has been used in several studies of MS treatment [16]. Use of the Spanish version has recently been reported in the treatment of actinic keratosis [17].
- MS Quality of Life-54 (MSQOL-54) was used as a measure of quality of life. A Spanish adaptation of this questionnaire is available. It is divided into 12 subscales and has 2 summary scores, one for the physical dimension and one for mental health [18]. Higher scores indicate better health.

In addition to the above, information was collected on the health resources used for managing MS included the number of visits to primary health care, number of visits to hospital, number of admissions to hospital (with duration), need for adaptation due to disability (home, car, workplace), and need for rehabilitation and informal care. Complementary questions were included to explore factors of potential value in predicting treatment satisfaction, benefits and drawbacks of treatment (multiple choice question with the options “attenuation in health deterioration”, “simple administration”, “safe administration”, “improvement in symptoms”, “fewer relapses”, and “absence of MRI activity”), adherence, and preferred route of administration assuming a similar efficacy (subcutaneous, intramuscular, infusion, oral). For further details on questions on health resource usage and other factors, see Supporting Information.

### Statistical analysis

Based on previous experience with the TSQM in MS patients in treatment with fingolimod, in which 85% of patients reported moderately, very or extremely good satisfaction, assuming an alpha of 0.05 (type 1 error), with a two tailed analysis, 440 patients would be needed (assuming fewer than 10% of patients excluded from the analysis due to incomplete or inconsistent data) to provide a precision of ±3.5%.

Descriptive statistics (mean ±SD or median with range and interquartile range or number and percentage as appropriate) were calculated. Given the limited amount of missing data, missing values were not accounted for in the analyses. For comparisons of continuous variables with a normal distribution, the t test was used both for paired and independent variables. Non-normally distributed variables were compared with the Mann-Whitney test (unpaired data) or the Wilcoxon test (paired data). Proportions were compared with the chi-squared test or Fisher exact test, as appropriate. P values of < 0.05 were considered significant. Calculations were performed with SPSS software (Version 22.2).

To investigate the factors that may potentially affect satisfaction, a linear regression model was constructed with overall satisfaction as the dependent variable and the individual responses to questions in the other domains (effectiveness, side effects, convenience) as the independent variables. A bivariate analysis was performed, with those with a P value of 0.200 or less retained in the model. The correlation between EDSS and TSMQ was assessed by calculating the Spearman correlation coefficient.

## Results

All of the 445 patients screened were enrolled into the study and these patients comprised the evaluable population. The mean±SD age of the patients was 41±10.2 years and approximately two-thirds were women and almost all were white (Table 1). Although the protocol permitted the enrollment of patients with clinically isolated syndrome, all patients had RRMS with 0.3±0.6 relapses in the year prior to enrollment and a baseline EDSS of 1.6±1.0.

**Table 1:** Demographics and clinical characteristics.

Variable	Evaluable patients (N = 445)
Age (years)	41.1±10.2
Female	300 (67.4)
Ethnic origin Caucasia Hispanic or Latino Asian Other	435 (97.8)4 (0.9)3 (0.7)3 (0.7)
Age at disease onset	31.5 (9.5) [n = 430]
Age at diagnosis of multiple sclerosis	33.4 (9.4)
Relapses prior to diagnosis	1.6 ± 0.9 [n = 421]
Relapses in the last year	0.3 ± 0.6 [n = 444]
Relapses in the last year with corticoid therapy	0.2 ± 0.5
Relapses in the last year leading to hospitalization	0.0 (0.2)
EDSS score at diagnosis	1.6 ± 1.0 [n = 336]

Data are mean ± SD or number (%). EDSS = expanded disability status scale; SD = standard deviation

The most frequently used agents were Avonex (28.5%), Rebif 44μg (24.5%) and Copaxone (22.5%), while Extavia was the least frequently used (3%) (Table 2). Mean treatment durations ranged from 71.5 months (Betaferon) to 33.7 months (Extavia).

**Table 2:** Overview of immunomodulatory therapy.

Treatment	Patients currently treated	Time in treatment, months	Number of prescriptions	
			During the entire period on treatment	Per month
**Avonex®** (interferon beta-1a IM)	127 (28.5%)	63.2 ± 53.9	62.2 ± 146.9	1 (1.7)
**Rebif® 44μg** (interferon beta-1a SC)	109 (24.5%)	65.6 ± 44.3	90.1 ± 225.3	1.4 (3.1)
**Copaxone®** (glatiramer acetate SC)	100 (22.5%)	38.9 ± 32.0	105.7 ± 407.1	3.0 (7.8)
**Betaferon®**(interferon beta-1b SC)	58 (13.0%)	71.5 ± 55.0	137.4 ± 388.4	2.0 (4.2)
**Rebif® 22μg** (interferon beta-1a SC)	37 (8.3%)	58.4 ± 44.0	150.7 ± 287.7	3.0 (5.0)
**Extavia®**(interferon beta-1b SC)	14 (3.1%)	33.7 ± 19.9	19.1 ± 20.3	0.6 (0.5)

Data are number (%) or mean ± SD (standard deviation). IM = intramuscular; SC = subcutaneous; IV = intravenous.

The mean±SD overall satisfaction according to the TSQM was 68.8±18.6 (Table 3). In the breakdown by individual components (effectiveness, side effects, and convenience), side effects had the highest score and convenience had the lowest score (higher scores indicate greater satisfaction). By individual treatment, the highest overall satisfaction was reported for Rebif 22 μg (72.4±20.3) and the lowest for Extavia (61.7±23.7). By individual components, for side effects, the highest score was reported for Copaxone (80.6±22.2) and the lowest Avonex (63.9±24.6). For effectiveness, patients were most satisfied with Rebif 44 μg (70.1±16.9) and least satisfied with Betaferon (63.2±17.9). Finally, in the case of convenience, Rebif 22 μg scored highest (69.4±17.4) and Betaferon scored lowest (55.5±17.2). According to the MSQOL-54, the overall population was in better mental health (mean±SD, 69.0±13.7) than physical health (mean±SD, 67.8±16.8).

**Table 3:** TSQM score-distributions.

Treatment Group	Effectiveness		Side effects		Convenience		Overall Satisfaction	
	n	mean ± SD	n	mean ± SD	n	mean ± SD	n	mean ± SD
**Overall**	**439**	**66.8 ± 18.7**	**438**	**72.5 ± 23.9**	**442**	**62.2 ± 19.2**	**443**	**68.8 ± 18.6**
Betaferon^®^	58	63.2 ± 17.9	57	78.2 ± 23.1	58	55.5 ± 17.2	58	64.8 ± 18.4
Rebif^®^ 22μg	37	65.6 ± 22.9	37	73.1 ± 23.6	37	69.4 ± 17.4	37	72.4 ± 20.3
Rebif^®^ 44μg	106	70.1 ± 16.9	105	71.1 ± 22.1	107	62.7 ± 18.5	108	71.0 ± 15.7
Copaxone^®^	99	65.2 ± 18.6	99	80.6 ± 22.2	100	62.0 ± 19.7	100	68.7 ± 17.8
Avonex^®^	125	67.4 ± 18.9	126	63.9 ± 24.6	126	62.7 ± 20.2	126	68.6 ± 20.3
Extavia^®^	14	65.5 ± 19.4	14	77.7 ± 24.1	14	63.7 ± 18.4	14	61.7 ± 23.7

Data are mean ± SD (standard deviation)

**Fig 2:**
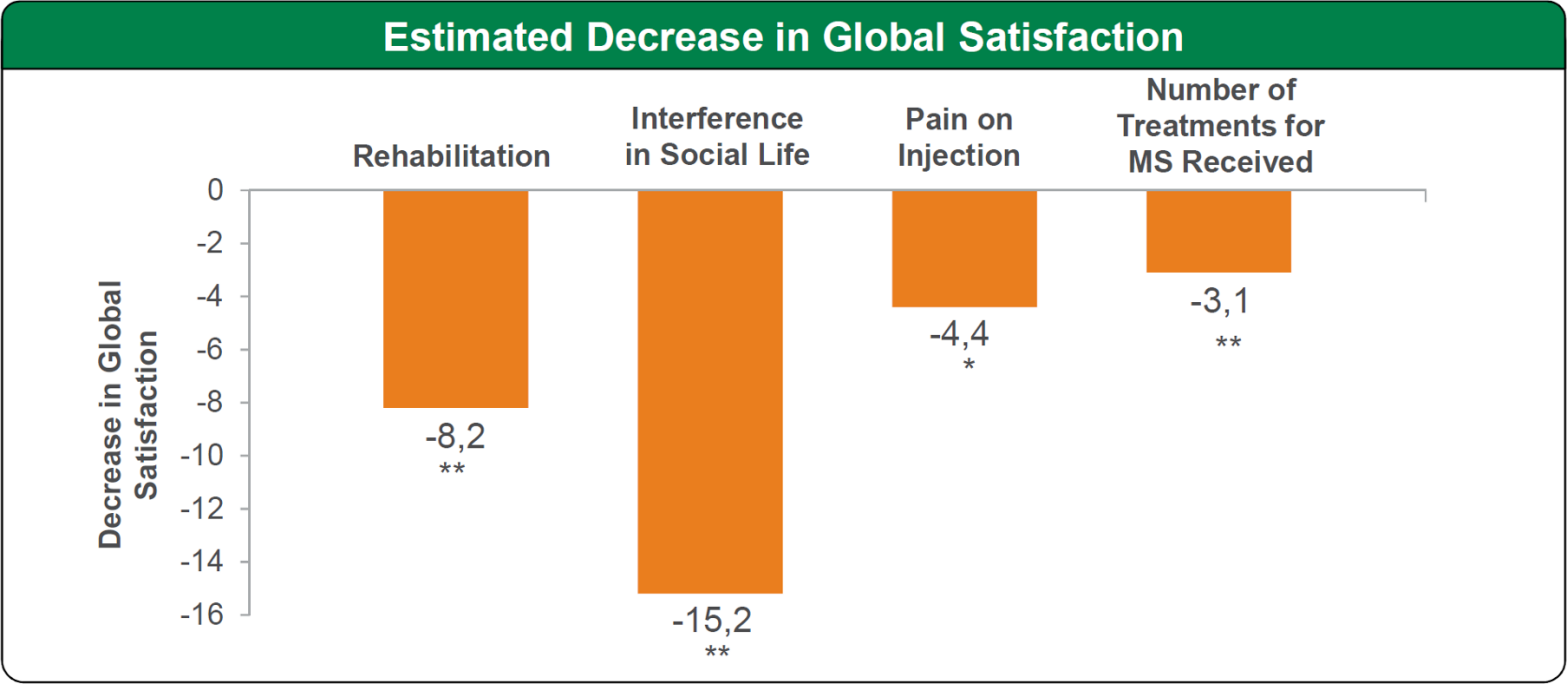
Estimated decrease in patient satisfaction*p<0.05; **p<0.01.Multivariate regression model. Changes in Global Satisfaction associated with “Difficulty in Preparing or Administering Treatment”and “Caregiver Support”were not significant.

A regression model was used to assess the impact on overall satisfaction of rehabilitation, interference with social life, pain on injection, and number of treatments for MS received (variables with a P value≤0.200 in the bivariate analysis). Rehabilitation, interference with social life, pain on injection and number of MS treatments received were significantly associated with a decrease in overall TSMQ score, with interference with social life having the biggest impact (Fig 2). Interference in social life was the potential factor with the biggest impact on side effects (-14.4, p<0.01). Although difficulty in preparing and administering treatment did not have a significant impact on overall satisfaction according to the linear regression model, a large but non-significant impact on convenience of -8.4 was noted (p = 0.069).

A significant but weak negative correlation was found between EDSS scores and TSMQ scores for overall satisfaction (rho = –0.11, p = 0.02) and effectiveness (rho = –0.17, p<0.001). For the other two dimensions, a smaller and non-significant negative correlation was also observed (rho = -0.031 for adverse effects and rho = -0.032 for convenience). Significant but weak negative correlations were also found between number of relapses in the year prior to the study and overall satisfaction (rho = –0.18, p<0.001) and effectiveness (rho = –0.16, p = 0.001). As before, small non-significant negative correlations were observed for the other two dimensions (rho = -0.002 for adverse effects and rho = -0.068 for convenience).

In the complementary questions about the benefits of their MS treatments, 412 (93%) reported at least one benefit. Most patients (250 [61%]) believed that therapy attenuated their health deterioration and almost half (202 [49%]) considered the treatments easy to administer. Among the options for response to the question on perceived benefits, absence of MRI activity was the one least frequently selected (112 patients [27%]). Within treatments, there were substantial variations in responses. For example, responses for “attenuation in health deterioration” ranged from 51% with Rebif 44 μg to 71% with Extavia while “ease of administration” responses ranged from 31% with Betaferon to 70% with Rebif 22 μg.

The main drawbacks of treatment were injection-site problems (243 patients [55%]) and general adverse reactions (193 patients [43%]). As with the benefits, variation was observed among treatments, with 37% of patients treated with Avonex reporting injection-site problems compared with 69% of those treated with Betaferon. For general adverse reactions, the highest proportion of reactions was reported for Avonex users (58%) and the lowest for Copaxone.

Overall, 35% patients had stopped medication or missed a dose. The 2 most frequently cited reasons for treatment interruption or missed doses were side effects (59.8%) and injection-site problems (48.9%).

**Fig 3:**
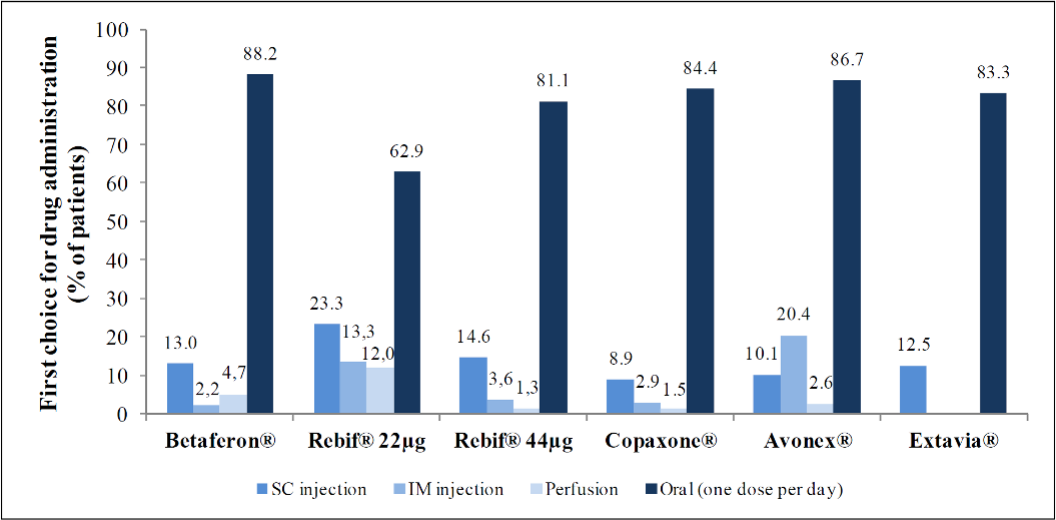
Preference for routes of administration.

Assuming a similar efficacy, as first choice DMT, most patients (83%) preferred once-daily oral treatment over other administration routes (Fig 3). When preference was for a route of administration other than oral, that route of administration usually corresponded to the actual route of administration of the patient.

## Discussion

Although efficacy and safety considerations remain central considerations in the choice of therapy in multiple sclerosis, with the recent increase in the number of treatments available, other patient-reported outcomes such as experience and satisfaction of patients with their treatment have become increasingly important [7]. The TSQM is a tool for assessing patient satisfaction with treatment. It was originally developed to assess treatment satisfaction in patients with chronic diseases such as arthritis, asthma, major depression, type I diabetes, high cholesterol, hypertension, migraine, and psoriasis [14]. This questionnaire has since been used in a number of studies of MS [7,19,20,21].

In the present study, we used the TSMQ to assess patient satisfaction with injectable DMTs. We found a mean overall TSQM score of 68.8±18.6. This compares with a score of approximately 75 for all treatments considered (IFNβ-1a IM, IFNβ-1a SC, glatiramer acetate, and natalizumab) in a study reported by Glanz et al [20] conducted in 226 patients in the United States. The treatment duration of patients receiving IFNβ-1a IM, IFNβ-1a SC and glatiramer acetate in that study was similar to that reported in our study and the EDSS scores also appeared similar. The main differences between the patient population in that study and in our study were age (the mean age in our study was 41 years whereas patients in the study by Glanz et al ranged from 45 years for IFNβ-1a SC to 51 years for IFNβ-1a IM) and disease duration (mean of approximately 8 years in our study compared with between 12 and 15 years in the study by Glanz et al). On analysis by the individual components of the TSMQ, we did find qualitative agreement between the two studies. Thus, side effects had the highest scores in both studies and convenience generally the lowest (except for the 22 μg dose of IFNβ-1a SC). The overall message is therefore perhaps that these injectable treatments are well-tolerated but patients find them relatively inconvenient.

More recently Haase et al [7] investigated the relationship between therapy satisfaction and adherence in patients with relapsing-remitting multiple sclerosis in a large study of 3312 patients in Germany. The overall TSQM scores were also a little higher than in our patients (71.4 for glatiramer acetate, and between 72.3 and 72.6 for interferons). In that study, the mean duration of therapy was 2.7 months, that is, considerably shorter than the duration in our study. We note that in that study, as in our study, patients with clinically isolated syndrome (CIS) could be included. While we eventually recruited no patients with CIS, 3.7% of the patients had CIS in the study by Haase et al.

Finally, a recent Spanish study of 220 patients that primarily investigated treatment compliance in patients with both RRMS and secondary-progressive MS also administered the TSQM [19]. Their study found a similar overall satisfaction according to the TSQM (69.8 versus 68.8 in our study).

In the analysis of association of factors such that might impact treatment satisfaction with overall treatment satisfaction, we found that interference in social life had the strongest association (p<0.05). This would seem to tie in with the overall message that injectable DMTs are well tolerated but the need to frequent injections is inconvenient for the patients in their everyday lives. Indeed, it is interesting to note that, across the injectable DMTs considered in the study, a large majority of patients expressed a preference for oral administration, despite being apparently well-managed with their current injectable DMT. A preference for oral treatment has been noted before, for example, in a study of patients who switched from an injectable DMT to oral fingolimod [22]. In the phase III study that compared oral teriflunomide with IFNβ-1a SC in 324 patients with RRMS, significant differences in TSMQ scores were observed in favor with teriflunomide and similar to those found in the present study, although no differences were found in the primary endpoint (time to treatment failure) [21].

Perceptions of efficacy are thought to be an important factor in treatment satisfaction with treatment [23]. This is consistent with the finding in our study of a significant negative correlation between EDSS scores and TSMQ scores for overall satisfaction and effectiveness. In the complementary questions, the majority of patients (61%) believed that treatment helped slow health deterioration. Interestingly, though, only 27% appreciated that reduction in MRI activity was a perceived benefit, although higher MRI activity may be associated with disease progression.

The main study weakness is that patients were required to be on stable injectable DMT for at least 6 months to be included in the study. Clearly, patients who discontinue their treatment within the first 6 months may be more likely to be unsatisfied with their treatment. The higher rate of discontinuations early in the treatment may be because the side effects of treatment decrease in intensity and frequency after the first 3 months of treatment [24,25]. Thus, patients who discontinue treatment in this period may be more a reflection of tolerability at initiation of treatment. The requirement for stable treatment does, therefore, mean that we can evaluate whether such patients are truly satisfied with the drug they are taking. Among these stable patients, a potential lack of treatment satisfaction can be identified in some cases. Ultimately, this suggests that treating physicians should be prepared to consider changing the treatment even in cases of patients who have been taking the same agent for a long time.

Another weakness of the study is that we did not collect quantitative adherence data and so we cannot investigate the impact of any lack of treatment satisfaction on this variable. Our questionnaire did ask about missed doses and reasons. Overall, 35% had stopped or missed dose, mainly for reasons related to side effects or injection site problems. Glanz et al [20] specifically assessed the impact of TSQM scores on adherence and found that lower convenience scores (rather than side effects scores) on TSQM were associated with lower adherence for IFNβ-1a SC- and GA-treated patients. In the aforementioned Spanish study, stratification of TSQM scores by whether patients were compliant or non-compliant showed higher scores for compliant patients (71.2) than for non-compliant patients (65.7) [19].

A further weakness of the study is that although 445 patients were included, very few patients were taking some of the treatments in the study. For example, only 14 patients were taking, Extavia, which was first approved by the European Medicines Agency in 2008, and launched in Spain in April 2009, much later than the other drugs included in this study.

In conclusion, patients who were on stable injectable DMT therapy for at least 6 months appeared reasonably satisfied with their treatment. In line with other studies, our results suggest that the main source of dissatisfaction with the current treatment is the inconvenience of the administration regimen. It is therefore not surprising that a sizeable proportion of patients would prefer an orally administered drug if efficacy and safety profiles were otherwise comparable.

## Supporting information

S1 File Product registry report. (DOCX)

S2 File Final statistical complete report. (DOC)
