# Supplementary material for: Treatment satisfaction with injectable disease-modifying therapies in patients with relapsing-remitting multiple sclerosis (the STICK study)
Source: PLoS One. 2017 Oct 19;12(10):e0185766. doi: 10.1371/journal.pone.0185766 (PMC5648132; doi:10.1371/journal.pone.0185766)
Supplement: S1 File — (DOCX) [file pone.0185766.s001.docx]

| [Product registry report](#TOC) |
| --- |
| Compound(s): Teriflunomide (INN)  Registry Title: Satisfaction with injectable immunomodulatory treatment in patients with clinically isolated syndrome or relapsing-remitting multiple sclerosis  Registry number: NONE0L07270  Registry name: STICK Study |
| Registry initiation date [date first patient in (FPI)]: 07-Oct-2014  Registry completion date [last patient completed/last patient out (LPO)]: 10-Mar-2015  Registry design: A national, multicenter, retrospective, observational post-authorization study  Report date: 22-Jan-2016 |
| This registry was performed in compliance with the guidelines for Good Epidemiology Practice. This report has been prepared based on the publication ‘Strengthening the Reporting of Observational Studies in Epidemiology (STROBE) – Guidelines for reporting observational studies – Ann Intern Med. 2007’*.*  Part or all of the information presented in this document may be unpublished material and should be treated as the confidential property of the Company. The use of this information or material must be restricted to the recipient for the agreed purpose and must not be disclosed to any unauthorized persons in any form, including publications and presentations, without the written consent of the Company. |

Table of contents

[Product registry report 1](#_Toc423597423)

[Table of contents 2](#_Toc423597424)

[SYNOPSIS 4](#_Toc423597425)

[Appendices 13](#_Toc423597426)

[1 Appendix I – administrative and legal considerations 14](#_Toc423597427)

[1.1 Ethical considerations 14](#_Toc423597428)

[1.1.1 Ethical principles 14](#_Toc423597429)

[1.1.2 Laws and regulations 14](#_Toc423597430)

[1.2 Data protection 14](#_Toc423597431)

[1.3 Record retention 14](#_Toc423597432)

[1.4 The Company audits and inspections by Competent Authorities (CA) 14](#_Toc423597433)

[1.5 Central laboratory 15](#_Toc423597434)

[1.6 Ownership of data and use of registry results 15](#_Toc423597435)

[1.7 STUDY consultants 15](#_Toc423597436)

[1.7.1 Scientific Committee and Charter 15](#_Toc423597437)

[1.7.2 National coordination 15](#_Toc423597438)

[1.7.3 Other experts/consultants 15](#_Toc423597439)

[1.8 Participating Physicians 16](#_Toc423597440)

[1.9 Study personnel 18](#_Toc423597441)

[1.9.1 Personnel involved in the registry 18](#_Toc423597442)

[1.9.2 The Company Internal Staff 18](#_Toc423597443)

[1.9.3 Contract Research Organization (CRO) 18](#_Toc423597444)

[2 Appendix II – tables and graphs 19](#_Toc423597445)

[2.1 OTHER DESCRIPTIVE data 19](#_Toc423597446)

[2.1.1 Factors of potential value in treatment satisfaction 19](#_Toc423597447)

[2.1.2 EDSS progression 20](#_Toc423597448)

[2.1.3 Health-related Quality of Life 20](#_Toc423597449)

[2.1.4 Health resources used for managing multiple sclerosis 21](#_Toc423597450)

[2.1.4.1 Visits to primary health care, to the hospital and number of hospitalizations and tests 21](#_Toc423597451)

[2.1.4.2 Adaptation, rehabilitation and informal care 23](#_Toc423597452)

[2.2 Deviations 24](#_Toc423597453)

[3 Appendix III – supportive documents 25](#_Toc423597454)

[3.1 Protocol 25](#_Toc423597455)

[3.2 Statistical analysis plan (SAP) 25](#_Toc423597456)

[3.2.1 Final Statistical Analysis Plan 25](#_Toc423597457)

[3.2.2 Changes from the final Statistical Analysis Plan 25](#_Toc423597458)

[3.3 Case report form (CRF)/ Patient questionnaire 25](#_Toc423597459)

[3.4 Patient informed consent 25](#_Toc423597460)

[3.5 Other documents relevant to the registry 25](#_Toc423597461)

[3.6 Other registry information 25](#_Toc423597462)

[3.6.1 Safety reporting 25](#_Toc423597463)

[3.6.1.1 Adverse events (AE) 25](#_Toc423597464)

[3.6.1.2 Serious adverse events (SAEs) 26](#_Toc423597465)

[3.6.1.3 Adverse events of Special Interest (AESI) 26](#_Toc423597466)

[3.7 Regulatory authorities’ submissions by country 29](#_Toc423597467)

[3.8 report approval 29](#_Toc423597468)

[3.8.1 Coordinating physician’s approval 29](#_Toc423597469)

[3.8.2 The Company’s approval 29](#_Toc423597470)

[4 Appendix IV - publications 30](#_Toc423597471)

[4.1 References 30](#_Toc423597472)

[4.2 Publications/abstracts of the registry results 30](#_Toc423597473)

[5 References 31](#_Toc423597474)

| SYNOPSIS | |
| --- | --- |
| **Title of the registry:** | Satisfaction with injectable immunomodulatory treatment in patients with clinically isolated syndrome or relapsing-remitting multiple sclerosis |
| **Design:** | The STICK study was an observational retrospective study conducted in neurology units of 35 Spanish hospitals.  The study was conducted in accordance with the Helsinki Declaration of the World Medical Association, all its amendments, and national regulations. Ethical and regulatory approval and patients’ informed consent were obtained. |
| **Objectives:** | Primary objective:  To measure patient satisfaction with immunomodulatory treatment for clinically isolated syndrome (CIS) and/or relapsing-remitting multiple sclerosis (RRMS).  Secondary objectives:   - To determine the main factors affecting treatment satisfaction. - To evaluate the impact of local/systemic reactions related to parenteral administration on treatment satisfaction. - To evaluate the impact of DMT’s treatment satisfaction in the patient adherence - To evaluate the effect of treatment satisfaction and adherence on the results of disease activity (EDSS and relapses). - To evaluate the effect of treatment satisfaction and adherence on the costs associated with disease management. |
| **Treatment:** | Study drugs were injectable immunomodulatory therapies approved for CIS and RRMS.  The study drugs were prescribed at all times in accordance with the local Summary of Product Characteristics and the standard clinical practice of each participating center. |
| **Scientific committee and**  **members:** | Dr. Oscar Fernández:  Director UGCI Neurología Hospitales Regional y Virgen de la Victoria de Málaga y Jefe de Servicio Neurología HRU Málaga, Spain |
| **Publications (reference):** | ECTRIMS 2015 Poster (P594)  SEN 2015 Oral Comunication |
| **Introduction** - **Background/rationale:** | Over recent years interest has increased in the patient’s opinion as part of the clinical assessment of treatment effectiveness. Evaluation of satisfaction with medication is probably the measurement that has been developed most, and though its role has been poorly defined up to now, research in different patient populations and types of treatment suggest that patients’ satisfaction with their medication is a good predictor of their adherence to treatment [1].  There are few studies to date on satisfaction with disease-modifying therapies for multiple sclerosis (MS), some of which are studies based on surveys of small groups of patients about a specific treatment [2,3]. For example, Twork et al.[3] evaluated satisfaction with medication using a Likert-type scale with 6 response levels (from 1= very satisfied to 6=very dissatisfied; the results showed the patients with MS were moderately satisfied with their treatment, and that those who were less satisfied were less compliant.  Validated questionnaires represent the most standardized tool to measure patient satisfaction with their medication. Specifically, the Treatment Satisfaction Questionnaire for Medication (TSQM) is a validated measure of the main dimensions of patient satisfaction with medication[1] that has been used in multiple sclerosis (MS) with oral drugs and injectable immunomodulators. Evaluation of satisfaction with fingolimod and health resources use compared to interferon beta and glatiramer acetate has shown a higher level of satisfaction with daily oral treatment, that could translate into improved compliance and effectiveness ([4]); in turn, in the TENERE study[5], the first head-to-head study comparing teriflunomide and interferon beta-1a SC 44µg (Rebif^®^), patients treated with teriflunomide showed a higher degree of satisfaction with medication compared with Rebif^®^ after 48 weeks of treatment. Recently, Glanz et al.[6] used the TSQM to evaluate satisfaction with injectable disease-modifying therapies and found no significant differences between the different treatments in terms of global satisfaction, but there were differences in terms of satisfaction with effectiveness, side effects and convencience.  However, despite the fact that current immunomodulatory drugs have improved the course of MS and quality of life of patients, long-term and frequent parental administration (even daily) may affect the patients’ perception of their medication and their expectations. Furthermore, while self-injection is a common practice, regular application of injections may be stressing for some patients and even difficult in those with little manual dexterity or impaired cognitive function [7].  Concern about the proper technique of self-injection and needle phobia, together with the perception of lack of efficacy and local/systemic adverse reactions are additional problems affecting acceptance of treatment and adherence to treatment [8-11]. Non-adherence rates to disease-modifying therapy that may even exceed 50% have been reported [12,13]. Hence, it is a common problem that may result in worsening of clinical outcomes and an increased rate of relapses [14], and number of hospitalizations due to MS [15].  The present study presents new data on satisfaction with disease-modifying treatments for MS. In addition to characterizing the patient in terms of sociodemographic and clinical characteristics, and quality of life, this study also provides evidence of the impact of local/systemic adverse reactions related to parenteral administration on treatment satisfaction, and the potential impact of greater/lesser satisfaction with medication on MS activity and resource use related to management of the disease. |
| **Methodology:** | **Patients**:  STICK was an observational retrospective study. Patients with clinically isolated syndrome (CIS) or relapsing-remitting multiple sclerosis (RRMS) were enrolled at 35 centers in Spain. Study participants were adults (≥18 years old) on treatment with one of the approved immunomodulatory therapies for at least 6 months [Avonex^®^, (interferon beta-1a intramuscular), Rebif^®^ (interferon beta-1a subcutaneous), Extavia^®^ (interferon beta-1b intravenous), Betaferon^®^ (interferon beta-1b subcutaneous), Copaxone^®^ (glatiramer acetate subcutaneous)] and had at least one prescription for CIS or RRMS treatment. All patients had to give the informed consent. Patients were excluded if they were participating in a clinical trial, or were unable to complete the study questionnaires.  The study was offered consecutively to the patients visited that met the study criteria. It was considerd that 5% of those patients would refuse to participate in the study which would not significant.  **Data collection procedures:**  The study design did impose a retrospective data collection in a single visit coinciding with any performed by the patients as part of routine follow-up of their disease, without interfering with usual clinical practice of the investigator.  The observational nature of the study was ensured as no diagnostic or therapeutic intervention outside of routine clinical practice was applied. Data collection was limited to those available in the medical record of selected patients in whom a specific therapeutic strategy had already been assigned based on routine practice, without interference with the physician’s prescription habits.  **Measures:**  Satisfaction with medication was evaluated by the Treatment Satisfaction Questionnaire for Medication (TSQM). The TSQM covers four domains of satisfaction with medication: effectiveness, side effects, convenience and global satisfaction. The scores for the domains range from 0 (extremely dissatisfied) to 100 (extremely satisfied) [1].  Patients’ disability was quantified according to Expanded Disability Status Scale (EDSS) scores and number of relapses in the prior year before inclusion.  Health-related quality of life was measured using the MSQOL-54 questionnaire [16].The scale consists of 54 items: 18 MS-specific items and 36 general health items, which are from the 36-item Short Form Health Status Sur­vey (SF-36). The scale is divided into 12 subscales and has two summary scores: the physical health compos­ite summary and the mental health composite summary. In each of the subscales, a higher score indicates better health.  Resources for managing MS included number of visits to primary health care (due to any cause other than MS, and due to MS); number of visits to the hospital (due to any cause other than MS, and due to MS); number of hospitalizations and duration (for any reason other than MS, and for MS); number of additional tests (for any reason other than MS, and for MS); number of visits to the hospital due to MS (by taxi or ambulance); need to adapt patient’ usual places (home, car, workplace); need for rehabilitation and informal care; and number of treatments received (for MS, and for concomitant diseases).  Other variables: patients were asked about factors of potential value in predicting treatment satisfaction, advantages/disadvantages of their current medication for MS, if they had discontinued a treatment or missed a dose indicating reasons and frequency, and preferences regarding the first choice of the route of administration of DMTs. Adverse reactions to current treatment were also retrieved.  **Data analysis:**  To explore satisfaction with medication for immunomodulatory drugs in CIS and RRMS patients as well as other descriptive data such as factors of potential value in predicting treatment satisfaction, health resources used by patients with MS, health-related quality of life and complementary questions, a descriptive analysis was used in both the overall population and treatment groups. Continuous variables are expressed as mean ± standard deviation (SD) or median (interquartile range, IQR) according to their distribution, and categorical variables as percentage.  To determine which factors could be affecting satisfaction,a multivariate linear regression model was constructed for each TSQM domain using the variables described bellow (Table 1).  Table 1. Factors included in regression model   \| **Drug** \|  \| \| --- \| --- \| \| **Injection-related factors** \| - Pain  - Injection-related anxiety  - Local/systemic reactions  - Interference with the social life  - Difficulty in the preparation or administration \| \| **Social factors** \| - Profession  - Family situation  - Caregiver support \| \| **Economical factors** \| - No. of visits to health primary care  - No. of visits to the hospital  - No. of hospitalizations  - No. of tests \| \| **Number of visits to the hospital due to MS** \| - By taxi or ambulance \| \| **Need to adapt patients’ usual places** \| - Home  - Car  - Workplace \| \| **Need for rehabilitation and informal care** \|  \|   The influence of local/systemic reactions related to the parenteral drugs on satisfaction with medication was evaluated by t-test (or Mann-Whitney test according to the sample distribution of the variables). The effect of treatment satisfaction on disability (by EDSS score and number of relapses) was investigated using ANOVA or Kruskal-Wallis tests according to the sample distribution of the variables. To determine the effect of treatment satisfaction on health resources used, the relationship between each TSQM-domain and resources variables was studied. Parametric tests such as *t*-test or ANOVA or non-parametric such as Mann-Whitney for dichotomous variables, Kruskal-Wallis for categorical or correlations for continuous variables were used.  Missing data were not considered in the analyses and a significance level of 0.05 was used for statistical testing. The statistical analyses were performed with the Statistical Package for the Social Sciences (SPSS) version 22.0 (SPSS Inc., Chicago, Illinois, USA). |
| **RESULTS** |  |
| **Participants (actual):** | **Number of Subjects Planned and Analyzed:**  440 planned  445 enrolled  0 selection failure  0 withdrawn  445 analyzed |
| **Participant characteristics and primary analyses:** | **Patient characteristics:**  A total of 445 patients were enrolled into the study. As there were no selection failures or withdrawn subjects, these 445 patients comprised the final evaluable population. Demographics and clinical characteristics are displayed in the table 2 below. On average, patients were 41.1 years old. Most were female (67.4%) and nearly all were Caucasian (97.8%).  Table 2. Demographics and clinical characteristics   \| **Variable** \| **Evaluable patients (N=445)** \| \| --- \| --- \| \| Age (years) \| 41.1±10.2 \| \| Female \| 300 (67.4) \| \| Ethnic origin  Caucasian  Hispanic or Latino  Asian  Other \| 435 (97.8)  4 (0.9)  3 (0.7)  3 (0.7) \| \| Age at beginning of symptoms \| 31.5 (9.5) [n=430] \| \| Age at diagnosis of multiple sclerosis \| 33.4 (9.4) \| \| Relapses prior to diagnosis \| 1.6 ± 0.9 [n=421] \| \| Relapses in the last year \| 0.3 ± 0.6 [n=444] \| \| Relapses in the last year with corticoid therapy \| 0.2 ± 0.5 \| \| Relapses in the last year leading to hospitalization \| 0.0 (0.2) \| \| EDSS score at diagnosis \| 1.6 ± 1.0 [n=336] \| \| Data are mean ± SD or number (%). EDSS = expanded disability status scale; SD = standard deviation \| \|   The most frequently used agents were Avonex^®^ (28.5%), Rebif^®^ 44µg (24.5%) and Copaxone^®^ (22.5%) (Table 3). Patients treated with Betaferon^®^ and Rebif^®^ 44µg had a longer treatment duration (71.5 ± 55.0; 65.6 ± 44.3, respectively). We found no numerical differences in EDSS progression in last year in both the overall population and treatment groups (Figure 2 of Appendix II).  Table 3. Overview of immunomodulatory therapy   \| **Treatment** \| **Patients currently treated** \| **Time in treatment, months** \| **Number of prescriptions** \| \| \| --- \| --- \| --- \| --- \| --- \| \| **During the entire period on treatment** \| **Per month** \| \| Avonex®  (interferon beta-1a IM) \| 127 (28.5) \| 63.2 ± 53.9 \| 62.2 ± 146.9 \| 1 (1.7) \| \| Rebif® 44µg  (interferon beta-1a SC) \| 109 (24.5) \| 65.6 ± 44.3 \| 90.1 ± 225.3 \| 1.4 (3.1) \| \| Copaxone®  (glatiramer acetate SC) \| 100 (22.5) \| 38.9 ± 32.0 \| 105.7 ± 407.1 \| 3.0 (7.8) \| \| Betaferon®  (interferon beta-1b SC) \| 58 (13.0) \| 71.5 ± 55.0 \| 137.4 ± 388.4 \| 2.0 (4.2) \| \| Rebif® 22µg  (interferon beta-1a SC) \| 37 (8.3) \| 58.4 ± 44.0 \| 150.7 ± 287.7 \| 3.0 (5.0) \| \| Extavia®  (interferon beta-1b SC) \| 14 (3.1) \| 33.7 ± 19.9 \| 19.1 ± 20.3 \| 0.6 (0.5) \| \| Data are number (%) or mean ± SD (standard deviation). IM=intramuscular; SC=subcutaneous; IV=intravenous. \| \| \| \| \|   **Primary analysis:**  Satisfaction with immunomodulatory therapy for CIS and RRMM  Table 4 presents the mean scores for all TSQM domains. Although statistical differences accros treatment groups were not analyzed, patients treated with Copaxone® reported the highest levels of satisfaction with the impact of side effects. Patients treated with Rebif® 22µg reported the highest Global satisfaction scores and Rebif® 44µg was found to be the treatment with highest Effectiveness satisfaction.  Table 4. TSQM score-distributions   \| **Treatment Group** \| **Effectiveness**  **(N=439)** \| **Side effects (N=438)** \| **Convenience**  **(N=442)** \| **Global Satisfaction**  **(N= 443)** \| \| --- \| --- \| --- \| --- \| --- \| \| Overall \| 66.8 ± 18.7 \| 72.5 ± 23.9 \| 62.2 ± 19.2 \| 68.8 ± 18.6 \| \| Betaferon^®^ \| 63.2 ± 17.9 \| 78.2 ± 23.1 \| 55.5 ± 17.2 \| 64.8 ± 18.4 \| \| Rebif^®^ 22µg \| 65.6 ± 22.9 \| 73.1 ± 23.6 \| 69.4 ± 17.4 \| 72.4 ± 20.3 \| \| Rebif^®^ 44µg \| 70.1 ± 16.9 \| 71.1 ± 22.1 \| 62.7 ± 18.5 \| 71.0 ± 15.7 \| \| Copaxone^®^ \| 65.2 ± 18.6 \| 80.6 ± 22.2 \| 62.0 ± 19.7 \| 68.7 ± 17.8 \| \| Avonex^®^ \| 67.4 ± 18.9 \| 63.9 ± 24.6 \| 62.7 ± 20.2 \| 68.6 ± 20.3 \| \| Extavia^®^ \| 65.5 ± 19.4 \| 77.7 ± 24.1 \| 63.7 ± 18.4 \| 61.7 ± 23.7 \| \| Data are mean ± SD (standard deviation) \| \| \| \| \| |
| **Other analyses:** | **Secondary objectives:**  1. Factors associated with treatment satisfaction  Table 5 presents the multivariate associations between the Injection-Related Factors, Social and Economical Factors, Number of Displacements due to MS (by taxi or ambulance), Rehabilitation, Drug and Number of Treatments for MS received, and satisfaction with medication. The following variables were significantly associated with a decrease in the four TSQM-domains: need for rehabilitation, interference with social life, pain, difficulty in preparing or administer the treatment (Injection-related Factors), need for caregiver support (Social Factors), and Number of Treatments for MS received.  Table 5. Multivariate linear regression model: estimated change in TSQM domains   \| **Variable** \| **Effectiveness** \| **Side effects** \| **Convenience** \| **Global Satisfaction** \| \| --- \| --- \| --- \| --- \| --- \| \| **Rehabilitation** \| - 10.3^a^ \|  \|  \| - 8.2 ^a^ \| \| **Interference with social life** \| - 9.2 ^b^ \| - 14.4 ^a^ \| - 12.7 ^a^ \| -15.2 ^a^ \| \| **Difficulty in preparing or administer the treatment** \|  \|  \| - 8.4 ^c^ \|  \| \| **Pain on injection** \|  \|  \| - 4.3 ^b^ \| - 4.4 ^b^ \| \| **Caregiver support** \|  \|  \| - 6.5 ^b^ \|  \| \| **Number of treatments for MS received** \|  \|  \|  \| - 3.1 ^a^ \| \| ^a^ p<0.01; ^b^ p<0.05; ^c^ p=0.069. \| \| \| \| \|   2. Impact of local and systemic reactions to parenteral administration on treatment satisfaction:  No significant differences were observed between patients who suffered adverse reactions (AR) to parenteral administration versus those who did not with regard to all TSQM-domains (Figure 1). [Mann-Whitney U test: Effectiveness (p=0.127); Side effects (0.393); Convenience (p=0.115); Global satisfaction (p=0.321)].    Figure 1. Mean in the TSQM-domains    3. Impact of disease modifying-treatments (DMTs) satisfaction on patient’s adherence to treatment  This objective could not be evaluated as there were insufficient data to generate an accurate answer.  4. Effect of treatment satisfaction on disease activity (by EDSS and number of relapses):   - *EDSS:* There was a negative correlation between EDSS scores and Effectiveness and Global Satisfaction TSQM-domain scores (for effectiveness r= - 0.17, p< 0.001; for global satisfaction r= - 0.11, p=0.02). - *Number of relapses*: The number of relapses in the prior year was negatively correlated with the Effectiveness and Global SatisfactionTSQM-domains (for effectiveness r = -0.16, p< 0.001; for global satisfaction r = - 0.18, p<0.001).   5. Effect of treatment adherence on disease activity (by EDSS and number of relapses)  This objective could not be evaluated as there were insufficient data to generate an accurate answer.  6. Effect of treatment satisfaction on health resources for managing multiple sclerosis  No differences were seen between patients who attended at least one *visit to primary health care* due to MS and those who did not with regard to all TSQM-domain scores (Mann-Whitney U test: Effectiveness (p=0.339); Side Effects (p=0.973); Convenience (p=0.175); Global Satisfaction (p=0.095).  These results were similar to those found for the variables *number of visits to the hospital due to MS* [Mann-Whitney U test: Effectiveness (p=0.461); Side Effects (p=0.992); Convenience (p=0.558); Global Satisfaction (p=0.372)], *number of hospitalizations due to MS* [(Mann-Whitney U test: Effectiveness (p=0.498); Side Effects (p=0.929); Convenience (p=0.186); Global Satisfaction (p=0.609)], *number of additional tests* *due to MS* [(Mann-Whitney U test: Effectiveness (p=0.227); Side Effects (p=0.846); Convenience (p=0.241); Global Satisfaction (p=0.533)]; *number of taxi trips* [(Mann-Whitney U test: Side Effects (p=0.109); Convenience (p=0.183); Global Satisfaction (p=0.163)], and *number of ambulance trips* (Mann-Whitney U test: Effectiveness (p=0.339); Side Effects (p=0.504); Convenience (p=0.236); Global Satisfaction (p=0.839)]. Only for the *number of taxi displacements did* the analysis reveal a significant difference in the domain “Effectiveness” in favor of patients who did not make any taxi displacement due to MS (p<0.05).  For patients who had not received *rehabilitation,* significantly better outcomes were measured on the domains Effectiveness (p<0.001) and Global Satisfaction (p<0.005); those patients who did not receive *informal care* showed significantly better outcomes on the domain Convenience (p<0.05). In addition, patients who did not need to adapt their car due to disability showed statistically significant better outcomes on the domain Side Effects (p<0.05).  7.- Effect of treatment adherence on the costs associated with disease management.  This objective could not be evaluated as there were insufficient data to generate an accurate answer.  **Other descriptive data:**   - Factors of potential value in predicting treatment satisfaction:   Among factors that could interfere with treatment satisfaction, injection-related factors and specifically local reactions and pain on injection were reported by nearly half of patients (47.6%, 46.3%, respectively). Social aspects that may have an impact on satisfaction with the MS treatment are the need for a caregiver (10.1%), and being disabled (4.9%). (Table 7 and Table 8 of Appendix II).   - Heath-related Quality of life:   According to the MSQOL-54, the overall population was in better mental health (mean±SD, 69.0±13.7) than physical health (mean±SD, 67.8±16.8). Conversely, patients treated with Extavia^®^ perceived their physical health as better than mental health. Table 9 of the Appendix II presents the descriptive statistic for MSQOL-4 composite scores by treatment group.   - Health resources used by patients with MS:   *Visits to primary health care, visits to the hospital, and number of hospitalizations -* All of these resources were used more often due to any reason other than MS (Figure 4, Figure 5, Figure 6, of Appendix II). For the overall population, the duration of hospitalizations due to MS was higher than that for any other reason.  *Number of additional tests-*The number of additional tests required by patients due to MS was higher that those performed for any other reason in both the overall population and treatment groups (Figure 7 of Appendix II).  *Adaptation, rehabilitation and informal care -* The percentage of patients who needed to adapt their car and workplace were higher among those treated with Betaferon® (Figure 8 of Appendix II). Patients treated with Betaferon^®^ were also those who needed rehabilitation and informal care most. (Figure 9 of Appendix II).   - Complementary questions:   The majority of patients both in the overall population (60.7%) and treatment groups believed that MS therapy had attenuated their health deterioration. Even so, 7.4% of patients did not indicate any advantage of their current MS medication. The most frequent concerns of patients related to MS therapy were injection-site problems (66.8%) and side effects (53.0%).  Overall, 34.6% of patients had stopped a medication or missed a dose. Similar data were observed in all treatment groups, except for Extavia^®^, in which patients were more likely to have discontinued the medication or missed a dose (57.1%). The two main reasons for treatment interruption were side effects (59.8%) and injection-site problems (48.9%). A cumbersome administration of the drug and poor effectiveness also constituted reasons for discontinuing treatment in 20.7% and 7.6% of patients, respectively.  When patients stated their preferences for a first choice of a disease-modifying treatment (DMT) in the case of similar efficacy, most of them would prefer daily oral medications (Figure 10 of Appendix II).  A summary of adverse events is displayed in the Section 4.6.1 Safety Reporting. |
| **Discussion:** | Patients’ dissatisfaction with treatment plays an important role in chronic disorders as it may alert the physician to possible poor adherence to treatment, with the subsequent effect on clinical effectiveness.  Despite the benefits of DMTs for MS, several concerns over their use have been described such as inconvenient methods and schedules of administration and side effects [3,17] that may affect adherence to therapy. In fact, non-adherence rates for MS injectable drugs from 21-25% [13,18] to above 45% [12,13] have been reported even with a high percentage of women participating who are believed to be more compliant than men [18]. In the STICK study, we found a lower but considerable proportion of patients who reported having discontinued a treatment or missed a dose (34.6%). Regarding reasons for discontinuing therapy, side effects (59.8%) and injection-related issues (49.9%) were the two most common causes, followed by a cumbersome administration (20.7%). A perceived lack of treatment effect, however, was not among the main causes influencing DMT discontinuation as previously reported [3,19]. Even so, 65.4% of patients were adherent to injectable DMTs, a rate of adherence between those reported in patients treated with DMTs for similar and longer periods of time [18,20].  Several survey-based studies that have examined DMT satisfaction have not found differences in satisfaction ratings between types of treatment [2,3]. A recent longitudinal analysis also has not shown differences in terms of global TSQM satisfaction scores but did find differences in terms of satisfaction with effectiveness, side effects and convenience [6]. Our study measured satisfaction ratings in CIS and RRMS treated with Betaferon^®^, two doses of Rebif^®^, Copaxone^®^, Avonex^®^ and Extavia^®^. In general, patients perceived their medication as effective and safe, and they were globally satisfied. However, as in other studies [6], ratings of convenience of use were not so high, particularly in the case of Betaferon^®^, which, together with Extavia^®,^ are the DMTs with the greatest number of doses administered per week. The fact that patients in the STICK study on first-line DMTs exhibited no disease progression and had fewer relapses than in the year prior to the study reflects, as in other studies, that perceived benefits of medication may predict satisfaction [2]. In fact, EDSS and relapses have been shown in our study to influence treatment satisfaction, specifically in the perceived effectiveness and global satisfaction. Although not evaluated, the results of a good quality of life in both mental and physical domains could be also influencing this observed patient satisfaction.  When asked about the advantages of their current DMT, more patients on Extavia^®^ than on Betaferon^®^, Rebif^®^, Copaxone^®^ and Avonex^®^ perceived a lesser deterioration of their health as the most important. As expected, periodic administration was stressed more frequently by patients treated with weekly Avonex^®^ and three times per week Rebif^®^ than with the daily Copaxone^®^ and every other day Betaferon^®^ or Extavia^®^. Injection-related factors were rated as the main disadvantages among patients treated with Copaxone^®^, Betaferon^®^ and Rebif^®^ 44µg, and side effects among those on Rebif^®^ 22 µg and Avonex^®^; indeed, most patients in all treatment groups would prefer daily oral medications as the first choice of DMT in a situation of similar efficacy. Despite the fact that local reactions and injection-site pain were given as factors that could theoretically influence treatment satisfaction, no statistical significant differences were seen when comparing patients who suffered adverse reactions to parenteral administration and those who did not in all domains of treatment satisfaction. It appears that patients’ beliefs and concerns about medication can be modified by their personal experience of effectiveness and side effects, leading to acceptance of treatment. In fact, the incidence of adverse events related to MS therapies tends to diminish over time on treatment, thus making it more likely to achieve long-term adherence [21].  In the STICK study the Injection-related Factors, specifically interference with social life and need for rehabilitation, were the strongest predictors of worse treatment satisfaction, the former affecting all domains of treatment satisfaction. It is possible that the impact of any treatment on usual activities influences satisfaction with a drug in a more realistic way since patients’ expectations are not met. Understanding that patients’ expectations can enhance their level of satisfaction reinforces the idea to focus on the patients’ beliefs concerning their disease management. Need for caregiver support, pain on injection and number of treatments for MS received were also negatively associated with all TSQM-domains. As for the latter, it is reasonable that satisfaction with the current treatment may be influenced by experience with previous DMTs [6].  Given the increase in use of resources expected from non-compliance and treatment failure [1,15], being satisfied with medication as a determinant of patient adherence should contribute to saving healthcare costs. However, in the STICK study no associations were found between satisfaction with medication and use of medical resources.  Apart from limitations inherent to all retrospective studies, in which data are limited to those available in the medical record collected as part of routine clinical care, comparisons between treatment groups with regard to satisfaction with medication, quality of life and issues related to advantages/disadvantages and treatment discontinuation are lacking. For this reason, it cannot be assumed that significant differences may or may not exist in favor of any treatment even though numerical differences were minimal. Also, this study did not analyze the individual domain items, which would have provided the specific aspects of therapy with which patients are satisfied or dissatisfied the most regarding the effectiveness, side effects and convenience of their medication (i.e. ability to treat or prevent condition, ability to relieve symptoms, bothersomeness of side effects, ease/difficulty of use and planning to use, confidence that taking medication is good, etc). |
| **Conclusions:** | The STICK study demonstrated that patients with CIS and RRMS are generally satisfied with their current DMT, being reflected in the high percentage of patients who are adherent to MS medication and well controlled.  Nonetheless, patients on injectable MS treatments face injection-related factors as the main disadvantage and concerns influencing treatment satisfaction; indeed, in a situation of similar efficacy, the majority of them would choose oral alternatives. The fact that some of these injection-related factors affect the satisfaction with medication both in patients who have experienced these reactions and those who did not, suggests there exists individual experiences and expectations, which if met, result in the acceptance of treatment. Therefore, we encourage clinicians to try to understand patient’s expectations in an attempt to maintan patient satisfaction and control health care costs. |
| **Date of report:** | 22-Jan-2016 |

# Appendices

# Appendix I – administrative and legal considerations

## Ethical considerations

### Ethical principles

This registry was conducted in accordance with the principles laid down by the 18th World Medical Assembly (Helsinki, 1964) including all subsequent amendments.

### Laws and regulations

This registry was conducted in compliance with all international guidelines, and national laws and regulations of the country(ies) in which the registry was performed, as well as any applicable guidelines.

Each participating country locally ensured that all necessary regulatory submissions (eg, IRB/IEC) were performed in accordance with local regulations including local data protection regulations.

Regulatory authorities’ submissions by country are presented in [Section](#_Ref347134774) 3.7 (Appendix III).

## Data protection

The patient's personal data and physician's personal data which were to be included in the Company’s databases were treated in compliance with all local applicable laws and regulations.

When archiving or processing personal data pertaining to the physician and/or to the patients, the Company took all appropriate measures to safeguard and prevent access to this data by any unauthorized third party.

## Record retention

The physician was responsible for the retention of the registry documentation until the end of the registry. In addition, the physician had to comply with specific local regulations and recommendations regarding patient record retention.

## The Company audits and inspections by Competent Authorities (CA)

The physician agreed to allow the Company’s auditors and Competent Authorities’ inspectors to have direct access to records of the registry for review, it being understood that all personnel with access to patients’ records are bound by professional secrecy and as such, could not disclose any personal identity or personal medical information.

The physician had to make every effort to help with the performance of the audits and inspections, giving access to all necessary facilities, data, and documents. As soon as notification from the authorities for an inspection was received by the physician, he/she had to inform the Company and authorize the Company to participate in this inspection. The confidentiality of the data to verify and the protection of the patients must be respected during these inspections. Any results or information arising from the inspections by the Competent Authorities were to be immediately communicated by the physician to the Company. The physician had to take appropriate measures required by the Company to ensure corrective actions for all problems found during audits and inspections.

## Central laboratory

Not applicable.

## Ownership of data and use of registry results

Unless otherwise specified by local laws and regulations, the Company retains ownership of data, results, reports, findings, and discoveries related to the registry. Therefore, the Company reserves the right to use the data from the present registry for any purpose, including to submit them to the Competent Authorities of any country.

The Study Committee, if any involved in the registry, has full access to the final data base allowing for appropriate academic analysis and reporting of the registry results.

## STUDY consultants

### Scientific Committee and Charter

Not applicable.

### National coordination

Not applicable.

### Other experts/consultants

Not applicable.

## Participating Physicians

The physicians performed the registry in accordance with the protocol, applicable local regulations and international guidelines.

The physician or a person designated by the physician, fully informed the patient, in language and terms they were able to understand, to the fullest extent possible, about the registry, objectives, constraints, duration, and patient’s rights.

It was the responsibility of the physician’s or a person designated by the physician to obtain written and signed informed consent from patients prior to inclusion. The patient’s legal representative could also sign the written informed consent form (ICF) on behalf of the patient. A copy of the signed and dated written ICF was provided to the patient and/ or his legal representative.

A list of all participating physicians is provided in the table below.

Table 6. List of principal investigators

| **Site no.** | **Site** | **City** | **PI** |
| --- | --- | --- | --- |
| 001 | Complejo Hospitalario de Navarra | Pamplona | Teresa Ayuso Blanco |
| 002 | Hospital Universitario Son Espases | Palma de Mallorca | María del Carmen de la Bandera Sánchez |
| 003 | Hospital Universitario Mutua de Terrassa | Terrassa | Inmaculada Bonaventura Ibars |
| 004 | Hospital Universitario Germans Trias i Pujol | Badalona | Anna Suñol Camas |
| 005 | Hospital del Mar | Barcelona | Elvira Munteis Olivas |
| 006 | Complejo Asistencial de Segovia | Segovia | Amelia Mendoza Rodríguez |
| 007 | Hospital St Pau | Barcelona | Antonio Escartin |
| 008 | Hospital San Pedro de Alcántara | Cáceres | Montserrat Gómez Gutiérrez |
| 009 | Hospital Universitario de Salamanca | Salamanca | Yasmina Berdei Montero |
| 010 | Hospital San Pedro | Logroño | Mª Eugenia Marzo Sola |
| 011 | Hospital de Galdakao-Usansolo | Galdakao | José Luís Sánchez Menoyo |
| 012 | Hospital San Agustín | Avilés | Joaquín Peña Martínez |
| 013 | CHOP, Complejo Hospitalario de Pontevedra | Pontevedra | María del Campo Amigo Jorrin |
| 014 | CHOP, Complejo Hospitalario de Pontevedra | Pontevedra | Ana Rodríguez Regal |
| 015 | Hospital Universitario Fundación Alcorcón | Alcorcón | Laura Borrega Canelo |
| 016 | Hospital Doce de Octubre | Madrid | Sara Moreno García |
| 017 | Hospital Virgen de las Nieves | Granada | Carmen Arnal Garcia |
| 018 | Hospital Torrecárdenas | Almería | Carmen Muñoz Fernández |
| 019 | Hospital Parc Taulí | Sabadell | Miguel Marco Igual |
| 020 | Hospital Regional U. Carlos Haya | Málaga | Alejandro Gallardo-Tur |
| 021 | Hospital Infanta Elena | Huelva | Eduardo Durán Ferreras |
| 022 | Hospital de Txagorritxu | Vitoria-Gasteiz | Amaya Álvarez de Arcaya Esquide |
| 023 | Hospital de Txagorritxu | Vitoria-Gasteiz | Francisco Julián-Villaverde |
| 024 | Hospital de León | León | Luís Hernández Echevarría |
| 025 | Hospital General U. de Elda | Elda | Javier Mallada Frechin |
| 026 | Hospital Marqués de Valdecilla | Santander | Agustín Oterino Duran |
| 027 | Hospital Nuestra Señora de Candelaria | Santa Cruz de Tenerife | Miguel Ángel Hernández Pérez |
| 028 | Complejo Hospitalario Universitario Materno Insular de Gran Canaria | Las Palmas de Gran Canaria | Miguel Hervás García |
| 029 | Hospital General de Castellón | Castellón | Antonio Belenguer Benavides |
| 030 | Fundación Jiménez Díaz | Madrid | Ricardo Constantino Ginestal López |
| 031 | Hospital Universitario de Guadalajara | Guadalajara | Antonio Yusta Izquierdo |
| 032 | Hospital Santa Tecla Tarragona | Tarragona | Jordi Batlle Nadal |
| 033 | Hospital Moisés Broggi | H. Sant Joan Despí | Ester Moral Torres |
| 034 | Hospital General Universitario Albacete | Albacete | Julia Gracia Gil |
| 035 | Hospital Morales Meseguer | Murcia | Ramón Villaverde González |

## Study personnel

### Personnel involved in the registry

The Coordinating physician’s and Company responsible medical officer’s signed approvals of the report are provided in [Section](#_Ref347149099) 3.8

This report was prepared by:

- Mireia Forner Andrés, Clinical Study Medical Manager, Genzyme
- Maria Dolores Pérez, Biostatistician, Dynamic S.L., Madrid
- Mireia Forner Andrés CTOM
- Isabel Caballero, Medical Writer, Dynamic S.L., Madrid

### The Company Internal Staff

The Company was responsible for providing adequate resources to ensure the proper conduct of the registry.

The Company was responsible for local submission(s) complying with data protection rules and any other local submission(s) required.

### Contract Research Organization (CRO)

Dynamic, S.L. Calle Sant Antoni Maria Claret, 434. 08027 Barcelona.

Data management, statistical activities and preparation of the report were carried out by Dynamic S.L. under the supervision of the Company.

# Appendix II – tables and graphs

## OTHER DESCRIPTIVE data

### Factors of potential value in treatment satisfaction

**Table 7. Injection-related factors**

| **Variable*** | **Evaluable patients (N=445)** |
| --- | --- |
| Local reactions | 212 (47.6) |
| Pain on injection | 206 (46.3) |
| Nervousness or anxiety caused by injection | 69 (15.5) |
| Self-injection or injection help | 69 (15.5) |
| Interference with the social life | 39 (8.8) |
| Difficulty in preparing or administration | 17 (3.8) |
| Others | 119 (26.7) |
| Data are number (%). Multiple response variables: percentages do not usually add up to 100% | |

**Table 8. Social factors**

| **Variable** | **Evaluable patients (N=445)** |
| --- | --- |
| **Profession** |  |
| Student/in training | 29 (6.5) |
| Part-time worker | 40 (9.0) |
| Full-time worker | 241 (54.2) |
| Housewife | 88 (19.8) |
| Disabled (100%) | 22 (4.9) |
| Retired | 25 (5.6) |
| **Structure of social relations** |  |
| Single/separated | 130 (29.2) |
| Cohabiting | 50 (11.2) |
| Married | 247 (55.5) |
| Divorced | 15 (3.4) |
| Widowed | 3 (0.7) |
| **Caregiver** | 45 (10.1) |
| Relative | 2 (4.4) |
| External | 43 (95.6) |
| Data are number (%). EDSS = expanded disability status scale; SD = standard deviation | |

### EDSS progression

**Figure 2. EDSS progression on DMT treatment**

### Health-related Quality of Life

**Table 9. MSQOL-54 composite scores**

| **Treatment Group** | **Physical Health**  **(N=419)** | **Mental Health (N=442)** |
| --- | --- | --- |
| Overall | 67.8 ± 16.8 | 69.0 ± 13.7 |
| Betaferon® | 65.4 ± 17.3 | 68.9 ± 13.2 |
| Rebif® 22µg | 69.2 ± 16.6 | 68.3 ± 13.2 |
| Rebif® 44µg | 69.7 ± 15.9 | 71.4 ± 11.7 |
| Copaxone® | 66.8 ± 16.5 | 67.1 ± 14.5 |
| Avonex® | 67.3 ± 17.6 | 69.0 ± 14.9 |
| Extavia® | 69.5 ± 19.1 | 65.5 ± 14.7 |
| Data are mean ± SD (standard deviation) | | |

### Health resources used for managing multiple sclerosis

#### Visits to primary health care, visits to the hospital, number and duration of hospitalizations and number of additional tests

Figure 3. Visits to primary health care

Figure 4. Visits to the hospital

Figure 5. Number of hospitalizations

Figure 6. Duration of hospitalizations

Figure 7. Number of additional tests

#### Adaptation, rehabilitation and informal care

Figure 8. Need to adapt usual places

Figure 9. Need for rehabilitation and informal care

### Complementary questions

Figure 10. First choice of a DMT

## Deviations

Not applicable

# Appendix III – supportive documents

## Protocol

File attached

## Statistical analysis plan (SAP)

### Final Statistical Analysis Plan

File attached

### Changes from the final Statistical Analysis Plan

Not applicable

## Case report form (CRF)/ Patient questionnaire

File attached

## Patient informed consent

File attached

## Other documents relevant to the registry

Not applicable

## Other registry information

Not applicable

### Safety reporting

#### Adverse events (AE)

The proportion of patients reporting adverse reactions was different across treatment groups. More patients treated with Avonex^®^ (49.6%), Rebif^®^ 44 µg (48.6%) and Copaxone^®^ (47.0%) reported side effects than those receiving Extavia^®^ (35.7%), Rebif^®^ 22 µg (35.1%) and Betaferon^®^ (34.5%).

In total, 201 (45.1%) patients reported having experienced at least one adverse event (AE). Most of AEs reported (238) were mild and non-serious, and all of them except four were possibly, probably, or remotely related to treatment. The only 4 AEs not related to treatment were: an event of autoinjector failure and of gastrointestinal disorder (during treatment with Copaxone^®^), an event of influenza-like illness (during treatment with Betaferon^®^) and an event of ocular disorder (during treatment with Rebif^®^ 44µg).

The most common events reported were influenza-like illness and injection-site reactions. A summary of all adverse reactions (RAs) reported throughout the study is shown in Table 10.

#### Serious adverse events (SAEs)

Two (2) serious adverse events (SAEs) were reported, 1 event of immune system disorder with Copaxone^®^ and 1 event of influenza-like illness with Avonex^®^.

#### Adverse events of Special Interest (AESI)

Not applicable.

| Table 10. Most common treatment-emergent adverse events | | | | | | | | | | | | |
| --- | --- | --- | --- | --- | --- | --- | --- | --- | --- | --- | --- | --- |
|  | **Betaferon®**  **Patients (n=58)** | | **Rebif® 22µg**  **Patients (n=37)** | | **Rebif® 44µg**  **Patients (n=109)** | | **Copaxone®**  **Patients (n=100)** | | **Avonex®**  **Patients (n=127)** | | **Extavia®**  **Patients (n=17)** | |
|  | **Mild** | **Moderate** | **Mild** | **Moderate** | **Mild** | **Moderate** | **Mild** | **Moderate** | **Mild** | **Moderate** | **Mild** | **Moderate** |
| **Systemic** |  |  |  |  |  |  |  |  |  |  |  |  |
| Influenza-like illness | 10 (17.2)* | - | 7 (18.9) | - | 20 (18.3) | 5 (4.6) | - | - | 42 (33.1) | 8 (6.3) | 1 (7.1) | - |
| Endocrine disorder | 1 (1.7) | - | - | - | 1 (0.9) | - | - | - | - | 1(0.8) | - | - |
| Hepatobiliary disorder | 1 (1.7) | - | - | - | - | 1 (0.9) | - | - | 3 (2.4) | - | - | - |
| Psychiatric disorder | - | - | 1 (2.7) | - |  |  | - | 1 (1) | - | - | - | - |
| Psychological disorder | - | - | - | - | - | 1 (0.9) | 1 (1) | - | - | - | - | - |
| Ocular disorder* | **-** | **-** | **-** | **-** | 1 (0.9) | - | **-** | **-** | **-** | **-** | **-** | **-** |
| Blood disorder | **-** | **-** | **-** | **-** | 2 (1.8) | - | **-** | **-** | 1(0.8) | **-** | **-** | **-** |
| Cardiac disorder | - | - | - | - | - | - | - | 1 (1) | - | - | - | - |
| Thoracic disorder | **-** | **-** | **-** | **-** | **-** | **-** | - | 1 (1) | **-** | **-** | **-** | **-** |
| Gastrointestinal disorder* | - | - | - | - | - | - | 1 (1) | - | - | - | - | - |
| Vascular disorder | **-** | **-** | **-** | **-** | **-** | **-** | **-** | **-** | - | 1(0.8) | **-** | **-** |
| **Local** |  |  |  |  |  |  |  |  |  |  |  |  |
| Injection-site reaction | 9 (15.5) | 1 (1.7) | 8 (21.6) | 1 (2.7) | 27 (24.8) | 9 (8.3) | 33 (33) | 7 (7) | 11 (8.7) | 3 (2.4) | 4 (28.6) | - |
| **Others** |  |  |  |  |  |  |  |  |  |  |  |  |
| Autoinjector failure* | - | - | - | - | - | - | 1 (1) | - | - | - | - | - |
| Interference when travelling | - | - | - | - | - | - | 1 (1) | - | - | - | - | - |
| Difficulty with the administration | - | - | - | - | - | - | - | - | 1 (0.8) | 1 (0.8) | - | - |
| Data are number (%). *Four adverse events were not related to treatment. | | | | | | | | | | | | |

## Regulatory authorities’ submissions by country

A copy of the document (memo, letter, form) attesting to the local regulatory authority approval of the Study will be provided upon request for all participating countries/regions.

## report approval

The final approved version of the report should be signed by the coordinating physician and by the Company.

### Coordinating physician’s approval

Add a copy of the coordinating physician’s approval of the registry report.

### The Company’s approval

Add a copy of the Company’s approval of the registry report.

# Appendix IV - publications

## References

## Publications/abstracts of the registry results

Add a copy of all publications/abstracts of the results of the registry.

# References

1 Atkinson MJ, Sinha A, Hass SL, Colman SS, Kumar RN, Brod M, et al. (2004) Validation of a general measure of treatment satisfaction, the Treatment Satisfaction Questionnaire for Medication (TSQM), using a national panel study of chronic disease. Health Qual Life Outcomes;2:12.

2 Turner AP, Kivlahan DR, Sloan AP, Haselkorn JK (2007) Predicting ongoing adherence to disease modifying therapies in multiple sclerosis: utility of the health beliefs model. Mult Scler;13:1146-52.

3 Twork S, Nippert I, Scherer P, Haas J, Pohlau D, Kugler J (2007) Immunomodulating drugs in multiple sclerosis: compliance, satisfaction and adverse effects evaluation in a German multiple sclerosis population. Curr Med Res Opin;23:1209-15.

4 Ziemssen T, Vollmar P, Meergans M, Mark Tracik F, Diaz Lorente M, Neidhardt K, et al. Interim Results of the PANGAEA and PEARL Studies, Comparing Treatment Satisfaction and Pharmaco-Economic Data of Fingolimod (Gilenya®) and First-Line Therapies in Multiple Sclerosis Patients in Germany . Neurology . 2013.

5 Vermersch P, Czlonkowska A, Grimaldi L, Confavreux C, Comi G, Kappos L (2012) Evaluation of patient satisfaction from the TENERE study: a comparison of teriflunomide and subcutaneous interferon beta-1a in patients with relapsing multiple sclerosis. J Neurol;1-236.

6 Glanz BI, Musallam A, Rintell DJ, Chitnis T, Weiner HL, Healy BC (2014) Treatment satisfaction in multiple sclerosis. Int J MS Care;16:68-75.

7 Devonshire V, Arbizu T, Borre B, Lang M, Lugaresi A, Singer B, et al. (2010) Patient-rated suitability of a novel electronic device for self-injection of subcutaneous interferon beta-1a in relapsing multiple sclerosis: an international, single-arm, multicentre, Phase IIIb study. BMC Neurol;10:28.

8 Cox D, Stone J (2006) Managing self-injection difficulties in patients with relapsing-remitting multiple sclerosis. J Neurosci Nurs;38:167-71.

9 Lugaresi A (2009) Addressing the need for increased adherence to multiple sclerosis therapy: can delivery technology enhance patient motivation? Expert Opin Drug Deliv;6:995-1002.

10 Mohr DC, Boudewyn AC, Likosky W, Levine E, Goodkin DE (2001) Injectable medication for the treatment of multiple sclerosis: the influence of self-efficacy expectations and injection anxiety on adherence and ability to self-inject. Ann Behav Med;23:125-32.

11 Tremlett HL, Oger J (2003) Interrupted therapy: stopping and switching of the beta-interferons prescribed for MS. Neurology;61:551-4.

12 Portaccio E, Zipoli V, Siracusa G, Sorbi S, Amato MP (2008) Long-term adherence to interferon beta therapy in relapsing-remitting multiple sclerosis. Eur Neurol;59:131-5.

13 Reynolds MW, Stephen R, Seaman C, Rajagopalan K (2010) Persistence and adherence to disease modifying drugs among patients with multiple sclerosis. Curr Med Res Opin;26:663-74.

14 Al-Sabbagh A, Bennet R, Kozma C, Dickson M, Meletiche D (2015) Medication gaps in disease-modifying therapy for multiple sclerosis are associated with an increased risk of relapse: findings from a national managed care database**.** J Neurol;S79.

15 Burks J, Malangone E, Jhaveri M, Zhou S, Stern L, Bhurke S, et al. The clinical outcomes associated with adherence to and discontinuation of disease-modifying treatments. 2012.

16 Aymerich M, Guillamon I, Perkal H, Nos C, Porcel J, Berra S, et al. (2006) [Spanish adaptation of the disease-specific questionnaire MSQOL-54 in multiple sclerosis patients]. Neurologia;21:181-7.

17 Klauer T, Zettl UK (2008) Compliance, adherence, and the treatment of multiple sclerosis. J Neurol;255 Suppl 6:87-92.

18 Devonshire V, Lapierre Y, Macdonell R, Ramo-Tello C, Patti F, Fontoura P, et al. (2011) The Global Adherence Project (GAP): a multicenter observational study on adherence to disease-modifying therapies in patients with relapsing-remitting multiple sclerosis. Eur J Neurol;18:69-77.

19 Rio J, Porcel J, Tellez N, Sanchez-Betancourt A, Tintore M, Arevalo MJ, et al. (2005) Factors related with treatment adherence to interferon beta and glatiramer acetate therapy in multiple sclerosis. Mult Scler;11:306-9.

20 Remington G, Rodriguez Y, Logan D, Williamson C, Treadaway K (2013) Facilitating medication adherence in patients with multiple sclerosis. Int J MS Care;15:36-45.

21 Patti F (2010) Optimizing the benefit of multiple sclerosis therapy: the importance of treatment adherence. Patient Prefer Adherence;4:1-9.
