# Supplementary material for: Treatment satisfaction with injectable disease-modifying therapies in patients with relapsing-remitting multiple sclerosis (the STICK study)
Source: PLoS One. 2017 Oct 19;12(10):e0185766. doi: 10.1371/journal.pone.0185766 (PMC5648132; doi:10.1371/journal.pone.0185766)
Supplement: S2 File — (DOC) [file pone.0185766.s002.doc]

STICK Study

“Satisfaction with injectable immunomodulatory treatment in patients with clinically isolated syndrome (CIS) or relapsing-remitting multiple sclerosis relapsing-remitting multiple sclerosis (RRMS)”

***Final Statistical Report***

***April 2015***

**Sponsor:** Sanofi

**Prepared by:** Mª Dolores Pérez Rodríguez

**Date:** April 2015

# TABLE OF CONTENTS

[1 INTRODUCTION 4](#__RefHeading___Toc489558878)

[2 OBJECTIVES 4](#__RefHeading___Toc489558879)

[2.1 Primary objective 4](#__RefHeading___Toc489558880)

[2.2 Secondary objectives 4](#__RefHeading___Toc489558881)

[3 STUDY POPULATION 5](#__RefHeading___Toc489558882)

[4 METHODOLOGY 6](#__RefHeading___Toc489558883)

[5 DESCRIPTIVE ANALYSIS 7](#__RefHeading___Toc489558884)

[5.1 Demographic and anthropometric data 7](#__RefHeading___Toc489558885)

[5.2 Medical history of multiple sclerosis 7](#__RefHeading___Toc489558886)

[5.3 Data on immunomodulatory treatment 9](#__RefHeading___Toc489558887)

[5.4 Current treatment 9](#__RefHeading___Toc489558888)

[5.5 Compliance during treatment 11](#__RefHeading___Toc489558889)

[5.6 Adverse events 12](#__RefHeading___Toc489558890)

[5.7 Factors related to satisfaction 15](#__RefHeading___Toc489558891)

[5.8 Resources associated with management of disease during current treatment 16](#__RefHeading___Toc489558892)

[5.9 Questionnaires 23](#__RefHeading___Toc489558893)

[6 ANALYSIS OF OBJECTIVES 32](#__RefHeading___Toc489558894)

[6.1 Primary objective 32](#__RefHeading___Toc489558895)

[6.2 Secondary objective 1 32](#__RefHeading___Toc489558896)

[6.3 Secondary objective 2 41](#__RefHeading___Toc489558897)

[6.4 Secondary objective 3 42](#__RefHeading___Toc489558898)

[6.5 Secondary objective 4 43](#__RefHeading___Toc489558899)

[6.6 Secondary objective 5 51](#__RefHeading___Toc489558900)

GLOSSARY

| PC | Primary care |
| --- | --- |
| CRF | Case report form |
| SD | Standard deviation |
| EDSS | Expanded Disability Status Scale |
| MS | Multiple sclerosis |
| RMS | Relapsing-remitting multiple sclerosis. |
| CI | Confidence interval |
| LCI | Lower confidence interval |
| UCL | Upper confidence interval |
| MSQOL-54 | Multiple Sclerosis Quality of Life Inventory |
| N | Number of patients |
| NA | Not available |
| Q1 | 1st quartile |
| Q3 | 3rd quartile |
| AR | Adverse reactions |
| CIS | Clinically isolated syndrome |
| TSQM | Treatment satisfaction questionnaire for medication |

# INTRODUCTION

The final statistical report of the STICK Study is presented below, a national, multicenter, retrospective, observational post-authorization study (PAS-OD). Data collection for the study was performed at a single visit only. This visit coincided with one to the visits made by the patient to his/her doctor during usual follow-up. Patient data were collected provided they were available in the patient’s medical history and in the questionnaires provided to the patients.

In the framework of the observational study, the treating physician did not receive guidelines on diagnosis, treatment or follow-up tests.

# OBJECTIVES

## Primary objective

To measure patient satisfaction with immunomodulatory treatment for clinically isolated syndrome (CIS) and/or relapsing-remitting multiple sclerosis (RRMS)

## Secondary objectives

- To determine the main factors affecting treatment satisfaction.
- To evaluate the impact of local/systemic reactions related to parenteral administration on treatment satisfaction.
- To evaluate the impact of satisfaction with immunomodulatory therapies on patient treatment adherence.
- To evaluate the effect of treatment satisfaction and adherence on the results of disease activity (EDSS and relapses).
- To determine the effect of treatment satisfaction and adherence on the costs associated with disease management.

# STUDY POPULATION

The study population was formed by patients meeting the following selection criteria:

**Inclusion criteria**

- Age ≥ 18 years.
- Patients diagnosed with clinically isolated syndrome (CIS) or relapsing-remitting multiple sclerosis (RRMS).
- Patients treated with only one of the following first-line immunomodulatory therapies for at least six months:
  - Avonex® (interferon beta-1a, intramuscular)
  - Rebif® (interferon beta-1a, subcutaneous)
  - Extavia® (interferon beta-1b)
  - Betaferon® (interferon beta-1b)
  - Copaxone® (glatiramer acetate)
- Patients with at least one prescription for treatment of RRMS or CIS.
- Written informed consent.

**Exclusion criteria**

- Patients who are participating in a clinical trial.
- Patients who are incapable of completing the study questionnaires.

At the time of database closure, 445 enrolled patients were available. All patients enrolled met the selection criteria so no patient was eliminated. The report was made with this population.

| **The number of evaluable patients for this report was 445.** |
| --- |

# METHODOLOGY

Quantitative variables are described with measures of central tendency and dispersion (mean, SD, median, minimum, maximum, Q_1_ and Q_3_). The number of patients until completing the total N of the study refers to the patients with no data.

Qualitative variables are described using absolute (N) and relative (%) frequencies. When two columns of percentages are presented, they refer to:

- Total percentage (%): Percentage over the sum of valid responses plus missing values, that is, over the total study sample.
- Percentage valid (% valid): Percentage over total valid responses, that is, with data in the variable.

When there are no missing values, the two percentages (total and valid) coincide and only one is presented.

For comparison of two means, parametric (Student’s t-test) or nonparametric (Mann-Whitney U test) statistical tests were used depending on sample distribution. When the variable factor has less than 10 patients in one of its categories, the result should be interpreted with caution.

For comparison of qualitative variables, the chi-squared or Fisher test were used depending on the sample distribution.

To study the correlation between two quantitative variables, the Pearson (parametric) or Spearman (nonparametric) correlation coefficient was used.

Multiple linear regressions were performed to study which factors influence treatment satisfaction.

In all cases, these tests were two-sided and with a significance level of 0.05. In cases where a p-value less than 0.05 appears, this indicates that the result is considered statistically significant.

The data were analyzed using the SPSS version 22.0 statistical program.

# DESCRIPTIVE ANALYSIS

## Demographic and anthropometric data

The age of the patient was calculated as the time from the patient’s date of birth to the date of the visit.

|  | **Mean** | **SD** | **Median** | **Minimum** | **Maximum** | **Q1** | **Q3** | **N** |
| --- | --- | --- | --- | --- | --- | --- | --- | --- |
| **Age (years)** | 41.1 | 10.2 | 40.6 | 18.6 | 89.0 | 32.6 | 47.8 | 445 |

|  | | **N** | **%** |
| --- | --- | --- | --- |
| **Sex** | **Male** | 145 | 32.6 |
|  | **Female** | 300 | 67.4 |
| **Total** | | 445 | 100.0 |

|  | | **N** | **%** |
| --- | --- | --- | --- |
| **Race** | **Caucasian** | 435 | 97.8 |
|  | **Asian** | 3 | 0.7 |
|  | **Arab** | 2 | 0.4 |
|  | **Hispanic or Latino** | 4 | 0.9 |
|  | **Other (*)** | 1 | 0.2 |
| **Total** | | 445 | 100.0 |

^(*)^ “Arab/Spanish”.

## Medical history of multiple sclerosis

Age at onset of symptoms was calculated as the number of years from the patient’s date of birth to the date of appearance of the first symptoms.

|  | **Mean** | **SD** | **Median** | **Minimum** | **Maximum** | **Q1** | **Q3** | **N** |
| --- | --- | --- | --- | --- | --- | --- | --- | --- |
| **Age at onset of symptoms (years)** | 31.5 | 9.5 | 30.4 | 11.9 | 76.6 | 24.7 | 37.3 | 430 |

Age at diagnosis was calculated as the number of years from the patient’s date of birth to the date of diagnosis of relapsing-remitting multiple sclerosis.

|  | **Mean** | **SD** | **Median** | **Minimum** | **Maximum** | **Q1** | **Q3** | **N** |
| --- | --- | --- | --- | --- | --- | --- | --- | --- |
| **Age at diagnosis of RRMS (years)** | 33.4 | 9.4 | 32.1 | 13.1 | 89.0 | 26.3 | 38.6 | 445 |

|  | **Mean** | **SD** | **Median** | **Minimum** | **Maximum** | **Q1** | **Q3** | **N** |
| --- | --- | --- | --- | --- | --- | --- | --- | --- |
| **Number of relapses prior to diagnosis** | 1.6 | 0.9 | 2.0 | 0.0 | 6.0 | 1.0 | 2.0 | 421 |

|  | **Mean** | **SD** | **Median** | **Minimum** | **Maximum** | **Q1** | **Q3** | **N** |
| --- | --- | --- | --- | --- | --- | --- | --- | --- |
| **EDSS score at diagnosis** | 1.6 | 1.0 | 1.5 | 0.0 | 4.5 | 1.0 | 2.0 | 336 |

## Data on immunomodulatory treatment

|  | **Mean** | **SD** | **Median** | **Minimum** | **Maximum** | **Q1** | **Q3** | **N** |
| --- | --- | --- | --- | --- | --- | --- | --- | --- |
| **Number of relapses in the last year** | 0.3 | 0.6 | 0.0 | 0.0 | 4.0 | 0.0 | 0.0 | 444 |
| **Number of relapses in the last year with corticosteroids** | 0.2 | 0.5 | 0.0 | 0.0 | 4.0 | 0.0 | 0.0 | 445 |
| **Number of relapses in the last year with hospitalization** | 0.0 | 0.2 | 0.0 | 0.0 | 4.0 | 0.0 | 0.0 | 445 |

## Current treatment

|  | | **N** | **%** |
| --- | --- | --- | --- |
| **Treatment** | **Betaferon®** | 58 | 13.0 |
|  | **Rebif® 22µg** | 37 | 8.3 |
|  | **Rebif® 44µg** | 109 | 24.5 |
|  | **Copaxone®** | 100 | 22.5 |
|  | **Avonex®** | 127 | 28.5 |
|  | **Extavia®** | 14 | 3.1 |
| **Total** | | 445 | 100.0 |

The tables shown below were calculated for total patients and per treatment.

Treatment time was defined as the number of months from the treatment start date to the date of the visit.

| **Treatment time (months)** | **Mean** | **SD** | **Median** | **Minimum** | **Maximum** | **Q1** | **Q3** | **N** |
| --- | --- | --- | --- | --- | --- | --- | --- | --- |
| **Betaferon®** | 71.5 | 55.0 | 59.4 | 5.9 | 214.6 | 20.0 | 113.7 | 58 |
| **Rebif® 22µg** | 58.4 | 44.0 | 41.2 | 6.0 | 184.9 | 29.9 | 85.5 | 37 |
| **Rebif® 44µg** | 65.6 | 44.3 | 57.7 | 6.6 | 193.6 | 30.0 | 93.8 | 109 |
| **Copaxone®** | 38.9 | 32.0 | 28.2 | 5.9 | 162.1 | 13.3 | 57.7 | 100 |
| **Avonex®** | 63.2 | 53.9 | 44.4 | 6.1 | 204.0 | 19.5 | 91.3 | 127 |
| **Extavia®** | 33.7 | 19.9 | 38.9 | 6.9 | 61.6 | 13.7 | 50.5 | 14 |
| **Total** | 58.1 | 47.2 | 44.9 | 5.9 | 214.6 | 20.4 | 80.0 | 445 |

|  | | **Did the patient experience adverse reactions during this treatment?** | | **Total** |
| --- | --- | --- | --- | --- |
|  |  | **No (1)** | **Yes (2)** |  |
| **Treatment** | **Betaferon®** | 29 (50.0%) | 29 (50.0%) | 58 (100.0%) |
|  | **Rebif® 22µg** | 20 (54.1%) | 17 (45.9%) | 37 (100.0%) |
|  | **Rebif® 44µg** | 40 (36.7%) | 69 (63.3%) | 109 (100.0%) |
|  | **Copaxone®** | 46 (46.0%) | 54 (54.0%) | 100 (100.0%) |
|  | **Avonex®** | 48 (37.8%) | 79 (62.2%) | 127 (100.0%) |
|  | **Extavia®** | 9 (64.3%) | 5 (35.7%) | 14 (100.0%) |
| **Total** | | 192 (43.1%) | 253 (56.9%) | 445 (100.0%) |

^(1)^ If a patient has specified “No AR” in the AR form, the value “No” is imputed.

^(2)^ If a patient has recorded at least one AR in the AR form, the value “Yes” is imputed.

| **EDSS score 1 year ago** | **Mean** | **SD** | **Median** | **Minimum** | **Maximum** | **Q1** | **Q3** | **N** |
| --- | --- | --- | --- | --- | --- | --- | --- | --- |
| **Betaferon®** | 2.2 | 1.9 | 2.0 | 0.0 | 6.5 | 1.0 | 3.5 | 55 |
| **Rebif® 22µg** | 1.6 | 1.6 | 1.5 | 0.0 | 6.0 | 0.0 | 2.4 | 36 |
| **Rebif® 44µg** | 1.5 | 1.3 | 1.0 | 0.0 | 7.0 | 1.0 | 2.0 | 107 |
| **Copaxone®** | 1.6 | 1.5 | 1.5 | 0.0 | 6.5 | 1.0 | 2.0 | 99 |
| **Avonex®** | 1.4 | 1.2 | 1.0 | 0.0 | 6.0 | 0.0 | 2.0 | 122 |
| **Extavia®** | 1.3 | 1.0 | 1.0 | 0.0 | 3.5 | 0.8 | 1.6 | 14 |
| **Total** | 1.6 | 1.4 | 1.5 | 0.0 | 7.0 | 1.0 | 2.0 | 433 |

| **Current EDSS score** | **Mean** | **SD** | **Median** | **Minimum** | **Maximum** | **Q1** | **Q3** | **N** |
| --- | --- | --- | --- | --- | --- | --- | --- | --- |
| **Betaferon®** | 2.2 | 2.0 | 2.0 | 0.0 | 6.5 | 0.0 | 3.5 | 57 |
| **Rebif® 22µg** | 1.5 | 1.5 | 1.0 | 0.0 | 6.0 | 0.0 | 2.4 | 36 |
| **Rebif® 44µg** | 1.5 | 1.4 | 1.0 | 0.0 | 7.0 | 1.0 | 2.0 | 108 |
| **Copaxone®** | 1.4 | 1.4 | 1.0 | 0.0 | 6.5 | 0.0 | 2.0 | 100 |
| **Avonex®** | 1.4 | 1.3 | 1.0 | 0.0 | 6.0 | 0.0 | 2.0 | 125 |
| **Extavia®** | 1.3 | 1.0 | 1.0 | 0.0 | 4.0 | 0.8 | 1.6 | 14 |
| **Total** | 1.6 | 1.5 | 1.0 | 0.0 | 7.0 | 0.0 | 2.0 | 440 |

| **Number of prescriptions during that period** | **Mean** | **SD** | **Median** | **Minimum** | **Maximum** | **Q1** | **Q3** | **N** |
| --- | --- | --- | --- | --- | --- | --- | --- | --- |
| **Betaferon®** | 137.4 | 388.4 | 7.0 | 1.0 | 2448.0 | 1.0 | 74.8 | 58 |
| **Rebif® 22µg** | 150.7 | 287.7 | 13.0 | 1.0 | 1368.0 | 1.0 | 164.0 | 37 |
| **Rebif® 44µg** | 90.1 | 225.3 | 13.0 | 1.0 | 1440.0 | 2.0 | 77.0 | 109 |
| **Copaxone®** | 105.7 | 407.1 | 12.0 | 1.0 | 3650.0 | 3.0 | 32.8 | 100 |
| **Avonex®** | 62.2 | 146.9 | 15.0 | 1.0 | 1008.0 | 3.0 | 52.0 | 127 |
| **Extavia®** | 19.1 | 20.3 | 12.5 | 1.0 | 56.0 | 1.8 | 42.3 | 14 |
| **Total** | 94.6 | 287.3 | 12.0 | 1.0 | 3650.0 | 2.0 | 52.0 | 445 |

## Compliance during treatment

The number of monthly prescription was calculated as the quotient of the total number of prescriptions divided by the number of months on treatment.

| **Number of monthly prescriptions** | **Mean** | **SD** | **Median** | **Minimum** | **Maximum** | **Q1** | **Q3** | **N** |
| --- | --- | --- | --- | --- | --- | --- | --- | --- |
| **Betaferon®** | 2.0 | 4.2 | 0.2 | 0.007 | 14.8 | 0.0 | 1.0 | 58 |
| **Rebif® 22µg** | 3.0 | 5.0 | 0.4 | 0.007 | 19.0 | 0.1 | 2.8 | 37 |
| **Rebif® 44µg** | 1.4 | 3.1 | 0.3 | 0.006 | 15.5 | 0.1 | 1.0 | 109 |
| **Copaxone®** | 3.0 | 7.8 | 0.6 | 0.009 | 31.9 | 0.2 | 1.0 | 100 |
| **Avonex®** | 1.0 | 1.7 | 0.2 | 0.005 | 12.2 | 0.1 | 1.0 | 127 |
| **Extavia®** | 0.6 | 0.5 | 0.5 | 0.026 | 1.3 | 0.1 | 1.0 | 14 |
| **Total** | 1.8 | 4.7 | 0.3 | 0.005 | 31.9 | 0.1 | 1.0 | 445 |

## Adverse events

In the AR form, a total of 238 AR reported by 201 patients were recorded. A total of 52 patients did not specify any AR in the form.

|  | | **Patient had at least 1 AR** | | **Total** |
| --- | --- | --- | --- | --- |
|  |  | **No** | **Yes** |  |
| **Treatment** | **Betaferon®** | 38 (65.5%) | 20 (34.5%) | 58 (100.0%) |
|  | **Rebif® 22µg** | 24 (64.9%) | 13 (35.1%) | 37 (100.0%) |
|  | **Rebif® 44µg** | 56 (51.4%) | 53 (48.6%) | 109 (100.0%) |
|  | **Copaxone®** | 53 (53.0%) | 47 (47.0%) | 100 (100.0%) |
|  | **Avonex®** | 64 (50.4%) | 63 (49.6%) | 127 (100.0%) |
|  | **Extavia®** | 9 (64.3%) | 5 (35.7%) | 14 (100.0%) |
| **Total** | | 244 (54.8%) | 201 (45.2%) | 445 (100.0%) |

Adverse reactions by severity are listed below. If a patient recorded the same adverse reaction with different severity, the highest grade was considered.

|  | **Severity** | | | | |
| --- | --- | --- | --- | --- | --- |
|  | **Mild** | **Moderate** | **Severe** | **Life-threatening** | **Total** |
| **Psychological disorder** | 0 (0%) | 1 (0.2%) | 0 (0%) | 0 (0%) | 1 (0.2%) |
| **Administration difficulty** | 1 (0.2%) | 1 (0.2%) | 0 (0%) | 0 (0%) | 2 (0.4%) |
| **Autoinjector failure** | 1 (0.2%) | 0 (0%) | 0 (0%) | 0 (0%) | 1 (0.2%) |
| **Interference when traveling** | 1 (0.2%) | 0 (0%) | 0 (0%) | 0 (0%) | 1 (0.2%) |
| **Injection site reactions** | 92 (20.7%) | 21 (4.7%) | 0 (0%) | 1 (0.2%) | 114 (25.6%) |
| **Flu-like symptoms** | 80 (18%) | 13 (2.9%) | 1 (0.2%) | 0 (0%) | 94 (21.1%) |
| **Cardiac disorder** | 0 (0%) | 1 (0.2%) | 0 (0%) | 0 (0%) | 1 (0.2%) |
| **Immune system disorder** | 0 (0%) | 0 (0%) | 0 (0%) | 1 (0.2%) | 1 (0.2%) |
| **Endocrine disorder** | 2 (0.4%) | 1 (0.2%) | 0 (0%) | 0 (0%) | 3 (0.7%) |
| **Gastrointestinal disorder** | 1 (0.2%) | 0 (0%) | 0 (0%) | 0 (0%) | 1 (0.2%) |
| **Hepatobiliary disorder** | 4 (0.9%) | 1 (0.2%) | 0 (0%) | 0 (0%) | 5 (1.1%) |
| **Eye disorder** | 1 (0.2%) | 0 (0%) | 0 (0%) | 0 (0%) | 1 (0.2%) |
| **Psychological disorder** | 1 (0.2%) | 1 (0.2%) | 0 (0%) | 0 (0%) | 2 (0.4%) |
| **Psychiatric disorder** | 1 (0.2%) | 1 (0.2%) | 0 (0%) | 0 (0%) | 2 (0.4%) |
| **Blood disorder** | 3 (0.7%) | 0 (0%) | 0 (0%) | 0 (0%) | 3 (0.7%) |
| **Thoracic disorder** | 0 (0%) | 1 (0.2%) | 0 (0%) | 0 (0%) | 1 (0.2%) |
| **Vascular disorder** | 0 (0%) | 1 (0.2%) | 0 (0%) | 0 (0%) | 1 (0.2%) |

Percentage calculated over total patients in the study (N=445).

| **BETAFERON®** | **Severity** | | | | |
| --- | --- | --- | --- | --- | --- |
|  | **Mild** | **Moderate** | **Severe** | **Life-threatening** | **Total** |
| **Injection site reactions** | 9 (15.5%) | 1 (1.7%) | (0%) | (0%) | 10 (17.2%) |
| **Flu-like symptoms** | 10 (17.2%) | 0 (0%) | (0%) | (0%) | 10 (17.2%) |
| **Endocrine disorder** | 1 (1.7%) | 0 (0%) | (0%) | (0%) | 1 (1.7%) |
| **Hepatobiliary disorder** | 1 (1.7%) | 0 (0%) | (0%) | (0%) | 1 (1.7%) |

Percentage calculated over total patients with Betaferon® (N=58).

| **REBIF22®** | **Severity** | | | | |
| --- | --- | --- | --- | --- | --- |
|  | **Mild** | **Moderate** | **Severe** | **Life-threatening** | **Total** |
| **Injection site reactions** | 8 (21.6%) | 1 (2.7%) | (0%) | (0%) | 9 (24.3%) |
| **Flu-like symptoms** | 7 (18.9%) | 0 (0%) | (0%) | (0%) | 7 (18.9%) |
| **Psychiatric disorder** | 1 (2.7%) | 0 (0%) | (0%) | (0%) | 1 (2.7%) |

Percentage calculated over total patients with Rebif22® (N=37).

| **REBIF44®** | **Severity** | | | | |
| --- | --- | --- | --- | --- | --- |
|  | **Mild** | **Moderate** | **Severe** | **Life-threatening** | **Total** |
| **Injection site reactions** | 27 (24.8%) | 9 (8.3%) | (0%) | (0%) | 36 (33%) |
| **Flu-like symptoms** | 20 (18.3%) | 5 (4.6%) | (0%) | (0%) | 25 (22.9%) |
| **Endocrine disorder** | 1 (0.9%) | 0 (0%) | (0%) | (0%) | 1 (0.9%) |
| **Hepatobiliary disorder** | 0 (0%) | 1 (0.9%) | (0%) | (0%) | 1 (0.9%) |
| **Eye disorder** | 1 (0.9%) | 0 (0%) | (0%) | (0%) | 1 (0.9%) |
| **Psychological disorder** | 0 (0%) | 1 (0.9%) | (0%) | (0%) | 1 (0.9%) |
| **Blood disorder** | 2 (1.8%) | 0 (0%) | (0%) | (0%) | 2 (1.8%) |

Percentage calculated over total patients with Rebif44® (N=109).

| **COPAXONE®** | **Severity** | | | | |
| --- | --- | --- | --- | --- | --- |
|  | **Mild** | **Moderate** | **Severe** | **Life-threatening** | **Total** |
| **Autoinjector failure** | 1 (1%) | 0 (0%) | (0%) | 0 (0%) | 1 (1%) |
| **Interference when traveling** | 1 (1%) | 0 (0%) | (0%) | 0 (0%) | 1 (1%) |
| **Injection site reactions** | 33 (33%) | 7 (7%) | (0%) | 1 (1%) | 41 (41%) |
| **Cardiac disorder** | 0 (0%) | 1 (1%) | (0%) | 0 (0%) | 1 (1%) |
| **Immune system disorder** | 0 (0%) | 0 (0%) | (0%) | 1 (1%) | 1 (1%) |
| **Gastrointestinal disorder** | 1 (1%) | 0 (0%) | (0%) | 0 (0%) | 1 (1%) |
| **Psychological disorder** | 1 (1%) | 0 (0%) | (0%) | 0 (0%) | 1 (1%) |
| **Psychiatric disorder** | 0 (0%) | 1 (1%) | (0%) | 0 (0%) | 1 (1%) |
| **Thoracic disorder** | 0 (0%) | 1 (1%) | (0%) | 0 (0%) | 1 (1%) |

Percentage calculated over total patients with Copaxone® (N=100).

| **AVONEX®** | **Severity** | | | | |
| --- | --- | --- | --- | --- | --- |
|  | **Mild** | **Moderate** | **Severe** | **Life-threatening** | **Total** |
| **Psychological disorder** | 0 (0%) | 1 (0.8%) | 0 (0%) | (0%) | 1 (0.8%) |
| **Administration difficulty** | 1 (0.8%) | 1 (0.8%) | 0 (0%) | (0%) | 2 (1.6%) |
| **Injection site reactions** | 11 (8.7%) | 3 (2.4%) | 0 (0%) | (0%) | 14 (11%) |
| **Flu-like symptoms** | 42 (33.1%) | 8 (6.3%) | 1 (0.8%) | (0%) | 51 (40.2%) |
| **Endocrine disorder** | 0 (0%) | 1 (0.8%) | 0 (0%) | (0%) | 1 (0.8%) |
| **Hepatobiliary disorder** | 3 (2.4%) | 0 (0%) | 0 (0%) | (0%) | 3 (2.4%) |
| **Blood disorder** | 1 (0.8%) | 0 (0%) | 0 (0%) | (0%) | 1 (0.8%) |
| **Vascular disorder** | 0 (0%) | 1 (0.8%) | 0 (0%) | (0%) | 1 (0.8%) |

Percentage calculated over total patients with Avonex® (N=127).

| **EXTAVIA®** | **Severity** | | | | |
| --- | --- | --- | --- | --- | --- |
|  | **Mild** | **Moderate** | **Severe** | **Life-threatening** | **Total** |
| **Injection site reactions** | 4 (28.6%) | (0%) | (0%) | (0%) | 4 (28.6%) |
| **Flu-like symptoms** | 1 (7.1%) | (0%) | (0%) | (0%) | 1 (7.1%) |

Percentage calculated over total patients with Extavia® (N=14).

All adverse reactions are possibly, probably or definitely related to the drug, except 4, which are listed below.

- Autoinjector failure. Not related. Current drug: Copaxone®.
- Gastrointestinal disorder Improbable. Current drug: Copaxone®.
- Flu-like symptoms. Not related. Current drug: Betaferon®.
- Eye disorder. Not related. Current drug: Rebif44®.

## Factors related to satisfaction

|  | | **N** | **% ^(*)^** |
| --- | --- | --- | --- |
| **Injection-related factors** | **Local reactions** | 212 | 47.6 |
|  | **Pain** | 206 | 46.3 |
|  | **Other** | 119 | 26.7 |
|  | **Injection anxiety** | 69 | 15.5 |
|  | **Self-injected or needs help to self-inject** | 69 | 15.5 |
|  | **Interference with social life** | 39 | 8.8 |
|  | **Difficulty in preparation or administration** | 17 | 3.8 |
| **Total** | | 445 | 100.0 |

^(*)^ Multiple response, so the sum of the percentages does not necessarily equal 100.

|  | | **N** | **%** |
| --- | --- | --- | --- |
| **Occupation** | **Pupil/Student/Trainee** | 29 | 6.5 |
|  | **Full-time worker** | 241 | 54.2 |
|  | **Part-time worker** | 40 | 9.0 |
|  | **Housekeeper** | 88 | 19.8 |
|  | **Disabled (100%)** | 22 | 4.9 |
|  | **Retired** | 25 | 5.6 |
| **Total** | | 445 | 100.0 |

| **Part-time worker, housekeeper or disabled** | | **N** | **%** |
| --- | --- | --- | --- |
| **Related to MS?** | **No** | 110 | 73.3 |
|  | **Yes** | 40 | 26.7 |
| **Total** | | 150 | 100.0 |

|  | | **N** | **%** |
| --- | --- | --- | --- |
| **Family status** | **Single/Independent/Separated** | 130 | 29.2 |
|  | **Unmarried couple** | 50 | 11.2 |
|  | **Married** | 247 | 55.5 |
|  | **Divorced** | 15 | 3.4 |
|  | **Widow(er)** | 3 | 0.7 |
| **Total** | | 445 | 100.0 |

|  | | **N** | **%** |
| --- | --- | --- | --- |
| **Caregiver?** | **No** | 400 | 89.9 |
|  | **Yes** | 45 | 10.1 |
| **Total** | | 445 | 100.0 |

|  | | **N** | **%** |
| --- | --- | --- | --- |
| **Type of caregiver** | **External staff** | 2 | 4.4 |
|  | **Family member** | 43 | 95.6 |
| **Total** | | 45 | 100.0 |

## Resources associated with management of disease during current treatment

### Number of visits to primary care

| **Number of visits to primary care for any cause** | **Mean** | **SD** | **Median** | **Minimum** | **Maximum** | **Q1** | **Q3** | **N** |
| --- | --- | --- | --- | --- | --- | --- | --- | --- |
| **Betaferon®** | 6.1 | 6.7 | 3.0 | 0.0 | 21.0 | 1.0 | 10.0 | 34 |
| **Rebif® 22µg** | 15.5 | 24.0 | 3.5 | 0.0 | 68.0 | 1.3 | 19.0 | 12 |
| **Rebif® 44µg** | 4.7 | 8.7 | 2.0 | 0.0 | 50.0 | 0.0 | 5.0 | 64 |
| **Copaxone®** | 5.3 | 8.4 | 3.0 | 0.0 | 52.0 | 1.0 | 5.8 | 60 |
| **Avonex®** | 7.7 | 11.6 | 2.0 | 0.0 | 56.0 | 0.0 | 11.8 | 76 |
| **Extavia®** | 2.8 | 4.6 | 0.0 | 0.0 | 11.0 | 0.0 | 7.0 | 9 |
| **Total** | 6.3 | 10.6 | 2.0 | 0.0 | 68.0 | 0.0 | 7.0 | 255 |

| **Number of visits to primary care for multiple sclerosis** | **Mean** | **SD** | **Median** | **Minimum** | **Maximum** | **Q1** | **Q3** | **N** |
| --- | --- | --- | --- | --- | --- | --- | --- | --- |
| **Betaferon®** | 5.0 | 13.2 | 0.0 | 0.0 | 70.0 | 0.0 | 3.0 | 35 |
| **Rebif® 22µg** | 1.3 | 5.3 | 0.0 | 0.0 | 23.0 | 0.0 | 0.0 | 19 |
| **Rebif® 44µg** | 0.8 | 2.1 | 0.0 | 0.0 | 10.0 | 0.0 | 0.0 | 70 |
| **Copaxone®** | 1.1 | 2.3 | 0.0 | 0.0 | 16.0 | 0.0 | 2.0 | 69 |
| **Avonex®** | 2.1 | 6.9 | 0.0 | 0.0 | 56.0 | 0.0 | 1.0 | 84 |
| **Extavia®** | 1.4 | 4.0 | 0.0 | 0.0 | 12.0 | 0.0 | 0.5 | 9 |
| **Total** | 1.8 | 6.4 | 0.0 | 0.0 | 70.0 | 0.0 | 1.0 | 286 |

### Number of visits to the hospital

| **Number of visits to the hospital for any cause** | **Mean** | **SD** | **Median** | **Minimum** | **Maximum** | **Q1** | **Q3** | **N** |
| --- | --- | --- | --- | --- | --- | --- | --- | --- |
| **Betaferon®** | 12.1 | 13.9 | 7.0 | 0.0 | 55.0 | 2.0 | 21.0 | 47 |
| **Rebif® 22µg** | 9.5 | 13.7 | 5.0 | 0.0 | 53.0 | 2.0 | 13.0 | 24 |
| **Rebif® 44µg** | 11.5 | 19.6 | 4.0 | 0.0 | 116.0 | 0.0 | 15.0 | 85 |
| **Copaxone®** | 6.7 | 11.9 | 3.0 | 0.0 | 79.0 | 0.0 | 8.0 | 77 |
| **Avonex®** | 12.6 | 24.4 | 5.0 | 0.0 | 170.0 | 1.0 | 12.5 | 101 |
| **Extavia®** | 5.1 | 6.5 | 2.5 | 0.0 | 21.0 | 0.8 | 8.5 | 10 |
| **Total** | 10.5 | 18.6 | 4.0 | 0.0 | 170.0 | 1.0 | 12.0 | 344 |

| **Number of visits to the hospital for multiple sclerosis** | **Mean** | **SD** | **Median** | **Minimum** | **Maximum** | **Q1** | **Q3** | **N** |
| --- | --- | --- | --- | --- | --- | --- | --- | --- |
| **Betaferon®** | 12.0 | 11.7 | 8.0 | 0.0 | 55.0 | 3.0 | 19.0 | 53 |
| **Rebif® 22µg** | 7.2 | 9.3 | 4.0 | 0.0 | 46.0 | 2.0 | 8.8 | 30 |
| **Rebif® 44µg** | 11.8 | 14.0 | 7.0 | 0.0 | 82.0 | 2.0 | 15.0 | 96 |
| **Copaxone®** | 8.2 | 8.9 | 5.0 | 0.0 | 58.0 | 3.0 | 10.0 | 92 |
| **Avonex®** | 11.0 | 17.6 | 6.0 | 0.0 | 110.0 | 3.0 | 12.0 | 108 |
| **Extavia®** | 6.1 | 6.4 | 4.0 | 0.0 | 21.0 | 1.0 | 10.0 | 13 |
| **Total** | 10.2 | 13.4 | 6.0 | 0.0 | 110.0 | 3.0 | 12.8 | 392 |

### Number of hospitalizations

| **Number of hospitalizations for any cause** | **Mean** | **SD** | **Median** | **Minimum** | **Maximum** | **Q1** | **Q3** | **N** |
| --- | --- | --- | --- | --- | --- | --- | --- | --- |
| **Betaferon®** | 0.4 | 1.0 | 0.0 | 0.0 | 5.0 | 0.0 | 0.0 | 50 |
| **Rebif® 22µg** | 0.2 | 0.5 | 0.0 | 0.0 | 2.0 | 0.0 | 0.0 | 27 |
| **Rebif® 44µg** | 0.1 | 0.5 | 0.0 | 0.0 | 3.0 | 0.0 | 0.0 | 91 |
| **Copaxone®** | 0.2 | 0.7 | 0.0 | 0.0 | 4.0 | 0.0 | 0.0 | 94 |
| **Avonex®** | 0.2 | 0.6 | 0.0 | 0.0 | 4.0 | 0.0 | 0.0 | 110 |
| **Extavia®** | 0.1 | 0.3 | 0.0 | 0.0 | 1.0 | 0.0 | 0.0 | 13 |
| **Total** | 0.2 | 0.7 | 0.0 | 0.0 | 5.0 | 0.0 | 0.0 | 385 |

| **Duration (in days) of hospitalization for any cause** | **Mean** | **SD** | **Median** | **Minimum** | **Maximum** | **Q1** | **Q3** | **N** |
| --- | --- | --- | --- | --- | --- | --- | --- | --- |
| **Betaferon®** | 9.7 | 10.1 | 4.0 | 1.0 | 25.0 | 1.0 | 20.0 | 7 |
| **Rebif® 22µg** | 6.5 | 9.0 | 2.5 | 1.0 | 20.0 | 1.3 | 15.8 | 4 |
| **Rebif® 44µg** | 7.3 | 6.5 | 7.0 | 1.0 | 14.0 | 1.0 | - | 3 |
| **Copaxone®** | 4.7 | 3.3 | 5.0 | 1.0 | 11.0 | 2.0 | 6.0 | 7 |
| **Avonex®** | 3.9 | 2.2 | 3.5 | 1.0 | 7.0 | 2.0 | 6.3 | 10 |
| **Extavia®** | 4.0 | - | 4.0 | 4.0 | 4.0 | 4.0 | 4.0 | 1 |
| **Total** | 6.0 | 6.3 | 4.0 | 1.0 | 25.0 | 2.0 | 7.0 | 32 |

| **Number of hospitalizations for multiple sclerosis** | **Mean** | **SD** | **Median** | **Minimum** | **Maximum** | **Q1** | **Q3** | **N** |
| --- | --- | --- | --- | --- | --- | --- | --- | --- |
| **Betaferon®** | 0.3 | 0.7 | 0.0 | 0.0 | 3.0 | 0.0 | 0.0 | 53 |
| **Rebif® 22µg** | 0.1 | 0.2 | 0.0 | 0.0 | 1.0 | 0.0 | 0.0 | 31 |
| **Rebif® 44µg** | 0.2 | 1.2 | 0.0 | 0.0 | 11.0 | 0.0 | 0.0 | 98 |
| **Copaxone®** | 0.1 | 0.5 | 0.0 | 0.0 | 4.0 | 0.0 | 0.0 | 98 |
| **Avonex®** | 0.0 | 0.2 | 0.0 | 0.0 | 1.0 | 0.0 | 0.0 | 112 |
| **Extavia®** | 0.0 | 0.0 | 0.0 | 0.0 | 0.0 | 0.0 | 0.0 | 14 |
| **Total** | 0.1 | 0.7 | 0.0 | 0.0 | 11.0 | 0.0 | 0.0 | 406 |

| **Duration (in days) of hospitalization for multiple sclerosis** | **Mean** | **SD** | **Median** | **Minimum** | **Maximum** | **Q1** | **Q3** | **N** |
| --- | --- | --- | --- | --- | --- | --- | --- | --- |
| **Betaferon®** | 6.1 | 5.3 | 5.5 | 1.0 | 15.0 | 1.0 | 11.0 | 8 |
| **Rebif® 22µg** | 10.5 | 13.4 | 10.5 | 1.0 | 20.0 | 1.0 | - | 2 |
| **Rebif® 44µg** | 5.0 | 6.2 | 3.0 | 0.0 | 14.0 | 0.5 | 11.5 | 4 |
| **Copaxone®** | 7.2 | 5.7 | 5.0 | 1.0 | 16.0 | 3.0 | 12.5 | 5 |
| **Avonex®** | 5.3 | 2.4 | 6.0 | 2.0 | 7.0 | 2.8 | 7.0 | 4 |
| **Total** | 6.4 | 5.6 | 5.0 | 0.0 | 20.0 | 1.0 | 9.0 | 23 |

### Number of tests / additional tests

| **Total number of tests/tests for multiple sclerosis** | **Mean** | **SD** | **Median** | **Minimum** | **Maximum** | **Q1** | **Q3** | **N** |
| --- | --- | --- | --- | --- | --- | --- | --- | --- |
| **Betaferon®** | 8.4 | 8.1 | 6.0 | 0.0 | 37.0 | 2.0 | 14.0 | 53 |
| **Rebif® 22µg** | 8.6 | 10.3 | 4.5 | 1.0 | 47.0 | 2.8 | 9.5 | 30 |
| **Rebif® 44µg** | 9.0 | 9.2 | 5.0 | 0.0 | 40.0 | 2.0 | 13.0 | 95 |
| **Copaxone®** | 5.1 | 8.1 | 3.0 | 1.0 | 75.0 | 2.0 | 6.0 | 91 |
| **Avonex®** | 8.7 | 9.2 | 5.0 | 0.0 | 51.0 | 3.0 | 11.0 | 107 |
| **Extavia®** | 5.9 | 5.1 | 4.0 | 1.0 | 15.0 | 2.0 | 12.0 | 14 |
| **Total** | 7.8 | 8.9 | 5.0 | 0.0 | 75.0 | 2.0 | 10.0 | 390 |

| **Total number of tests/tests for related diseases** | **Mean** | **SD** | **Median** | **Minimum** | **Maximum** | **Q1** | **Q3** | **N** |
| --- | --- | --- | --- | --- | --- | --- | --- | --- |
| **Betaferon®** | 2.0 | 6.3 | 0.0 | 0.0 | 38.0 | 0.0 | 1.0 | 42 |
| **Rebif® 22µg** | 0.3 | 0.7 | 0.0 | 0.0 | 2.0 | 0.0 | 0.0 | 19 |
| **Rebif® 44µg** | 0.7 | 2.0 | 0.0 | 0.0 | 10.0 | 0.0 | 0.5 | 69 |
| **Copaxone®** | 1.0 | 3.3 | 0.0 | 0.0 | 17.0 | 0.0 | 0.0 | 70 |
| **Avonex®** | 2.9 | 8.0 | 0.0 | 0.0 | 52.0 | 0.0 | 2.0 | 80 |
| **Extavia®** | 0.3 | 0.9 | 0.0 | 0.0 | 3.0 | 0.0 | 0.0 | 10 |
| **Total** | 1.5 | 5.3 | 0.0 | 0.0 | 52.0 | 0.0 | 1.0 | 290 |

### Number of trips for multiple sclerosis

| **Number of ambulance trips for multiple sclerosis** | **Mean** | **SD** | **Median** | **Minimum** | **Maximum** | **Q1** | **Q3** | **N** |
| --- | --- | --- | --- | --- | --- | --- | --- | --- |
| **Betaferon®** | 0.0 | 0.0 | 0.0 | 0.0 | 0.0 | 0.0 | 0.0 | 52 |
| **Rebif® 22µg** | 0.0 | 0.0 | 0.0 | 0.0 | 0.0 | 0.0 | 0.0 | 31 |
| **Rebif® 44µg** | 0.0 | 0.4 | 0.0 | 0.0 | 4.0 | 0.0 | 0.0 | 95 |
| **Copaxone®** | 0.0 | 0.0 | 0.0 | 0.0 | 0.0 | 0.0 | 0.0 | 93 |
| **Avonex®** | 0.0 | 0.1 | 0.0 | 0.0 | 1.0 | 0.0 | 0.0 | 112 |
| **Extavia®** | 0.0 | 0.0 | 0.0 | 0.0 | 0.0 | 0.0 | 0.0 | 14 |
| **Total** | 0.0 | 0.2 | 0.0 | 0.0 | 4.0 | 0.0 | 0.0 | 397 |

| **Number of taxi trips for multiple sclerosis** | **Mean** | **SD** | **Median** | **Minimum** | **Maximum** | **Q1** | **Q3** | **N** |
| --- | --- | --- | --- | --- | --- | --- | --- | --- |
| **Betaferon®** | 0.1 | 0.4 | 0.0 | 0.0 | 3.0 | 0.0 | 0.0 | 49 |
| **Rebif® 22µg** | 0.0 | 0.0 | 0.0 | 0.0 | 0.0 | 0.0 | 0.0 | 30 |
| **Rebif® 44µg** | 0.0 | 0.0 | 0.0 | 0.0 | 0.0 | 0.0 | 0.0 | 90 |
| **Copaxone®** | 0.0 | 0.0 | 0.0 | 0.0 | 0.0 | 0.0 | 0.0 | 90 |
| **Avonex®** | 0.3 | 1.8 | 0.0 | 0.0 | 15.0 | 0.0 | 0.0 | 107 |
| **Extavia®** | 0.0 | 0.0 | 0.0 | 0.0 | 0.0 | 0.0 | 0.0 | 13 |
| **Total** | 0.1 | 1.0 | 0.0 | 0.0 | 15.0 | 0.0 | 0.0 | 379 |

### Did he/she have to adapt?

|  | | **Did home have to be adapted?** | | | **Total** |
| --- | --- | --- | --- | --- | --- |
|  |  | **No** | **Yes** | **NA** |  |
| **Treatment** | **Betaferon®** | 54 (93.1%) | 4 (6.9%) | 0 (0.0%) | 58 (100.0%) |
|  | **Rebif® 22µg** | 33 (89.2%) | 3 (8.1%) | 1 (2.7%) | 37 (100.0%) |
|  | **Rebif® 44µg** | 105 (96.3%) | 4 (3.7%) | 0 (0.0%) | 109 (100.0%) |
|  | **Copaxone®** | 96 (96.0%) | 2 (2.0%) | 2 (2.0%) | 100 (100.0%) |
|  | **Avonex®** | 122 (96.1%) | 3 (2.4%) | 2 (1.6%) | 127 (100.0%) |
|  | **Extavia®** | 13 (92.9%) | 1 (7.1%) | 0 (0.0%) | 14 (100.0%) |
| **Total** | | 423 (95.1%) | 17 (3.8%) | 5 (1.1%) | 445 (100.0%) |

|  | | **Did car have to be adapted?** | | | **Total** |
| --- | --- | --- | --- | --- | --- |
|  |  | **No** | **Yes** | **NA** |  |
| **Treatment** | **Betaferon®** | 54 (93.1%) | 3 (5.2%) | 1 (1.7%) | 58 (100.0%) |
|  | **Rebif® 22µg** | 35 (94.6%) | 1 (2.7%) | 1 (2.7%) | 37 (100.0%) |
|  | **Rebif® 44µg** | 108 (99.1%) | 0 (0.0%) | 1 (0.9%) | 109 (100.0%) |
|  | **Copaxone®** | 98 (98.0%) | 1 (1.0%) | 1 (1.0%) | 100 (100.0%) |
|  | **Avonex®** | 125 (98.4%) | 0 (0.0%) | 2 (1.6%) | 127 (100.0%) |
|  | **Extavia®** | 14 (100.0%) | 0 (0.0%) | 0 (0.0%) | 14 (100.0%) |
| **Total** | | 434 (97.5%) | 5 (1.1%) | 6 (1.3%) | 445 (100.0%) |

|  | | **Did workplace has to be adapted?** | | | **Total** |
| --- | --- | --- | --- | --- | --- |
|  |  | **No** | **Yes** | **NA** |  |
| **Treatment** | **Betaferon®** | 55 (94.8%) | 3 (5.2%) | 0 (0.0%) | 58 (100.0%) |
|  | **Rebif® 22µg** | 34 (91.9%) | 0 (0.0%) | 3 (8.1%) | 37 (100.0%) |
|  | **Rebif® 44µg** | 104 (95.4%) | 3 (2.8%) | 2 (1.8%) | 109 (100.0%) |
|  | **Copaxone®** | 95 (95.0%) | 3 (3.0%) | 2 (2.0%) | 100 (100.0%) |
|  | **Avonex®** | 119 (93.7%) | 2 (1.6%) | 6 (4.7%) | 127 (100.0%) |
|  | **Extavia®** | 14 (100.0%) | 0 (0.0%) | 0 (0.0%) | 14 (100.0%) |
| **Total** | | 421 (94.6%) | 11 (2.5%) | 13 (2.9%) | 445 (100.0%) |

### Has he/she received rehabilitation?

|  | | **Has he/she received rehabilitation?** | | | **Total** |
| --- | --- | --- | --- | --- | --- |
|  |  | **No** | **Yes** | **NA** |  |
| **Treatment** | **Betaferon®** | 47 (81.0%) | 11 (19.0%) | 0 (0.0%) | 58 (100.0%) |
|  | **Rebif® 22µg** | 31 (83.8%) | 4 (10.8%) | 2 (5.4%) | 37 (100.0%) |
|  | **Rebif® 44µg** | 93 (85.3%) | 16 (14.7%) | 0 (0.0%) | 109 (100.0%) |
|  | **Copaxone®** | 86 (86.0%) | 13 (13.0%) | 1 (1.0%) | 100 (100.0%) |
|  | **Avonex®** | 109 (85.8%) | 17 (13.4%) | 1 (0.8%) | 127 (100.0%) |
|  | **Extavia®** | 13 (92.9%) | 1 (7.1%) | 0 (0.0%) | 14 (100.0%) |
| **Total** | | 379 (85.2%) | 62 (13.9%) | 4 (0.9%) | 445 (100.0%) |

### Has he/she received informal care?

|  | | **Has he/she received informal care?** | | | **Total** |
| --- | --- | --- | --- | --- | --- |
|  |  | **No** | **Yes** | **NA** |  |
| **Treatment** | **Betaferon®** | 50 (86.2%) | 8 (13.8%) | 0 (0.0%) | 58 (100.0%) |
|  | **Rebif® 22µg** | 33 (89.2%) | 2 (5.4%) | 2 (5.4%) | 37 (100.0%) |
|  | **Rebif® 44µg** | 104 (95.4%) | 5 (4.6%) | 0 (0.0%) | 109 (100.0%) |
|  | **Copaxone®** | 95 (95.0%) | 3 (3.0%) | 2 (2.0%) | 100 (100.0%) |
|  | **Avonex®** | 120 (94.5%) | 6 (4.7%) | 1 (0.8%) | 127 (100.0%) |
|  | **Extavia®** | 13 (92.9%) | 1 (7.1%) | 0 (0.0%) | 14 (100.0%) |
| **Total** | | 415 (93.3%) | 25 (5.6%) | 5 (1.1%) | 445 (100.0%) |

### Number of pharmacological treatment received by the patient

| **Number of pharmacological treatment received by the patient for MS** | **Mean** | **SD** | **Median** | **Minimum** | **Maximum** | **Q1** | **Q3** | **N** |
| --- | --- | --- | --- | --- | --- | --- | --- | --- |
| **Betaferon®** | 1.3 | 0.7 | 1.0 | 0.0 | 3.0 | 1.0 | 2.0 | 55 |
| **Rebif® 22µg** | 1.0 | 0.8 | 1.0 | 0.0 | 4.0 | 1.0 | 1.0 | 36 |
| **Rebif® 44µg** | 1.2 | 0.8 | 1.0 | 0.0 | 4.0 | 1.0 | 1.0 | 103 |
| **Copaxone®** | 1.4 | 0.9 | 1.0 | 0.0 | 5.0 | 1.0 | 2.0 | 98 |
| **Avonex®** | 1.3 | 0.8 | 1.0 | 0.0 | 4.0 | 1.0 | 2.0 | 118 |
| **Extavia®** | 1.9 | 2.0 | 1.0 | 0.0 | 7.0 | 1.0 | 2.5 | 14 |
| **Total** | 1.3 | 0.9 | 1.0 | 0.0 | 7.0 | 1.0 | 2.0 | 424 |

| **Number of pharmacological treatment received by the patient for concomitant diseases** | **Mean** | **SD** | **Median** | **Minimum** | **Maximum** | **Q1** | **Q3** | **N** |
| --- | --- | --- | --- | --- | --- | --- | --- | --- |
| **Betaferon®** | 1.3 | 2.3 | 0.0 | 0.0 | 12.0 | 0.0 | 1.8 | 52 |
| **Rebif® 22µg** | 0.6 | 1.8 | 0.0 | 0.0 | 10.0 | 0.0 | 1.0 | 30 |
| **Rebif® 44µg** | 0.9 | 1.6 | 0.0 | 0.0 | 9.0 | 0.0 | 1.0 | 88 |
| **Copaxone®** | 1.2 | 1.8 | 0.0 | 0.0 | 9.0 | 0.0 | 2.0 | 94 |
| **Avonex®** | 0.9 | 1.5 | 0.0 | 0.0 | 9.0 | 0.0 | 1.0 | 112 |
| **Extavia®** | 1.9 | 3.7 | 0.0 | 0.0 | 13.0 | 0.0 | 2.0 | 13 |
| **Total** | 1.0 | 1.8 | 0.0 | 0.0 | 13.0 | 0.0 | 1.0 | 389 |

## Questionnaires

### TSQM Questionnaire

The degree of satisfaction questionnaire, TSQM, consists of 14 questions rated on a Likert type scale and classified into 4 categories (domains):

- Treatment effectiveness
- Adverse reactions
- Convenience
- Overall satisfaction

The score of each domains ranges from 0 (lowest degree of satisfaction) to 100 (highest degree of satisfaction).

| **Effectiveness** | **Mean** | **SD** | **Median** | **Minimum** | **Maximum** | **Q1** | **Q3** | **N** |
| --- | --- | --- | --- | --- | --- | --- | --- | --- |
| **Betaferon®** | 63.2 | 17.9 | 66.7 | 0.0 | 100.0 | 55.6 | 72.2 | 58 |
| **Rebif® 22µg** | 65.6 | 22.9 | 66.7 | 0.0 | 100.0 | 55.6 | 77.8 | 37 |
| **Rebif® 44µg** | 70.1 | 16.9 | 72.2 | 0.0 | 100.0 | 66.7 | 83.3 | 106 |
| **Copaxone®** | 65.2 | 18.6 | 66.7 | 16.7 | 100.0 | 55.6 | 77.8 | 99 |
| **Avonex®** | 67.4 | 18.9 | 66.7 | 0.0 | 100.0 | 58.3 | 80.6 | 125 |
| **Extavia®** | 65.5 | 19.4 | 66.7 | 33.3 | 100.0 | 48.6 | 83.3 | 14 |
| **Total** | 66.8 | 18.7 | 66.7 | 0.0 | 100.0 | 55.6 | 77.8 | 439 |

| **Adverse reactions** | **Mean** | **SD** | **Median** | **Minimum** | **Maximum** | **Q1** | **Q3** | **N** |
| --- | --- | --- | --- | --- | --- | --- | --- | --- |
| **Betaferon®** | 78.2 | 23.1 | 87.5 | 25.0 | 100.0 | 62.5 | 100.0 | 57 |
| **Rebif® 22µg** | 73.1 | 23.6 | 75.0 | 25.0 | 100.0 | 53.1 | 100.0 | 37 |
| **Rebif® 44µg** | 71.1 | 22.1 | 68.8 | 25.0 | 100.0 | 50.0 | 100.0 | 105 |
| **Copaxone®** | 80.6 | 22.2 | 87.5 | 12.5 | 100.0 | 68.8 | 100.0 | 99 |
| **Avonex®** | 63.9 | 24.6 | 62.5 | 18.8 | 100.0 | 43.8 | 87.5 | 126 |
| **Extavia®** | 77.7 | 24.1 | 90.6 | 37.5 | 100.0 | 54.7 | 100.0 | 14 |
| **Total** | 72.5 | 23.9 | 68.8 | 12.5 | 100.0 | 50.0 | 100.0 | 438 |

| **Convenience** | **Mean** | **SD** | **Median** | **Minimum** | **Maximum** | **Q1** | **Q3** | **N** |
| --- | --- | --- | --- | --- | --- | --- | --- | --- |
| **Betaferon®** | 55.5 | 17.2 | 54.2 | 16.7 | 100.0 | 41.7 | 66.7 | 58 |
| **Rebif® 22µg** | 69.4 | 17.4 | 66.7 | 25.0 | 100.0 | 58.3 | 79.2 | 37 |
| **Rebif® 44µg** | 62.7 | 18.5 | 66.7 | 25.0 | 100.0 | 50.0 | 75.0 | 107 |
| **Copaxone®** | 62.0 | 19.7 | 66.7 | 16.7 | 100.0 | 50.0 | 75.0 | 100 |
| **Avonex®** | 62.7 | 20.2 | 66.7 | 0.0 | 100.0 | 50.0 | 75.0 | 126 |
| **Extavia®** | 63.7 | 18.4 | 62.5 | 33.3 | 91.7 | 50.0 | 83.3 | 14 |
| **Total** | 62.2 | 19.2 | 66.7 | 0.0 | 100.0 | 50.0 | 75.0 | 442 |

| **Overall satisfaction** | **Mean** | **SD** | **Median** | **Minimum** | **Maximum** | **Q1** | **Q3** | **N** |
| --- | --- | --- | --- | --- | --- | --- | --- | --- |
| **Betaferon®** | 64.8 | 18.4 | 67.9 | 0.0 | 100.0 | 57.1 | 71.4 | 58 |
| **Rebif® 22µg** | 72.4 | 20.3 | 71.4 | 14.3 | 100.0 | 60.7 | 85.7 | 37 |
| **Rebif® 44µg** | 71.0 | 15.7 | 71.4 | 28.6 | 100.0 | 57.1 | 78.6 | 108 |
| **Copaxone®** | 68.7 | 17.8 | 71.4 | 21.4 | 100.0 | 57.1 | 78.6 | 100 |
| **Avonex®** | 68.6 | 20.3 | 71.4 | 0.0 | 100.0 | 57.1 | 78.6 | 126 |
| **Extavia®** | 61.7 | 23.7 | 53.6 | 14.3 | 100.0 | 50.0 | 82.1 | 14 |
| **Total** | 68.8 | 18.6 | 71.4 | 0.0 | 100.0 | 57.1 | 78.6 | 443 |

### MSQOL-54 questionnaire

The quality of life questionnaire specific for multiple sclerosis, MSQOL-54, consists of 54 items, 52 distributed in 12 dimensions, plus 2 individual items that measure changes in health status and satisfaction with sexual function. The 12 dimensions are:

- Physical health
- Role limitations for physical problems
- Limitations for emotional problems
- Pain
- Emotional wellbeing
- Energy
- Health perception
- Social function
- Cognitive function
- Health concern
- Sexual function
- Overall quality of life

The total score of each dimension has a range from 0 to 100, where the higher value indicates better health-related quality of life. Two subtotals corresponding to two scales are obtained:

- Physical health
- Mental health

| **Physical health** | **Mean** | **SD** | **Median** | **Minimum** | **Maximum** | **Q1** | **Q3** | **N** |
| --- | --- | --- | --- | --- | --- | --- | --- | --- |
| **Betaferon®** | 65.4 | 17.3 | 67.5 | 25.7 | 93.5 | 53.7 | 80.8 | 57 |
| **Rebif® 22µg** | 69.2 | 16.6 | 75.8 | 24.9 | 89.3 | 58.6 | 81.0 | 35 |
| **Rebif® 44µg** | 69.7 | 15.9 | 75.2 | 22.8 | 91.7 | 56.4 | 82.6 | 106 |
| **Copaxone®** | 66.8 | 16.5 | 68.4 | 20.8 | 95.3 | 51.4 | 81.3 | 97 |
| **Avonex®** | 67.3 | 17.6 | 72.4 | 2.2 | 93.5 | 54.9 | 79.7 | 114 |
| **Extavia®** | 69.5 | 19.1 | 76.9 | 40.8 | 89.8 | 47.1 | 85.2 | 10 |
| **Total** | 67.8 | 16.8 | 71.1 | 2.2 | 95.3 | 55.6 | 81.2 | 419 |

| **Mental health** | **Mean** | **SD** | **Median** | **Minimum** | **Maximum** | **Q1** | **Q3** | **N** |
| --- | --- | --- | --- | --- | --- | --- | --- | --- |
| **Betaferon®** | 68.9 | 13.2 | 71.9 | 36.9 | 89.9 | 61.1 | 79.0 | 58 |
| **Rebif® 22µg** | 68.3 | 13.2 | 73.2 | 33.4 | 86.5 | 57.6 | 78.5 | 37 |
| **Rebif® 44µg** | 71.4 | 11.7 | 74.5 | 29.5 | 88.1 | 64.3 | 79.8 | 107 |
| **Copaxone®** | 67.1 | 14.5 | 69.4 | 10.5 | 89.5 | 58.6 | 78.9 | 100 |
| **Avonex®** | 69.0 | 14.9 | 73.6 | 6.7 | 89.8 | 60.2 | 79.8 | 126 |
| **Extavia®** | 65.5 | 14.7 | 71.8 | 36.4 | 84.4 | 52.2 | 75.8 | 14 |
| **Total** | 69.0 | 13.7 | 72.6 | 6.7 | 89.9 | 60.5 | 79.3 | 442 |

### Additional questions

|  | | **N** | **% total ^(*)^** | **% valid ^(*)^** |
| --- | --- | --- | --- | --- |
| **For you personally, what are the advantages of the drugs you are currently taking or have taken for MS?** | **Less deterioration of my health** | 250 | 56.2 | 60.7 |
|  | **Simple administration** | 202 | 45.4 | 49.0 |
|  | **Safe administration** | 185 | 41.6 | 44.9 |
|  | **Improvement of my symptoms** | 182 | 40.9 | 44.2 |
|  | **Fewer relapses** | 174 | 39.1 | 42.2 |
|  | **Periodic administration** | 148 | 33.3 | 35.9 |
|  | **No inflammatory activity in MS** | 112 | 25.2 | 27.2 |
| **Patients who indicated at least one advantage** | | 412 | 92.6 | 100.0 |
| **Patients who did not indicate any advantage** | | 33 | 7.4 | - |
| **Total patients** | | 445 | 100.0 | - |

^(*)^ Multiple response, so the sum of the percentages does not necessarily equal 100.

| **For you personally, what are the advantages of the drugs you are currently taking or have taken for MS?** | **Betaferon®** | | | **Rebif® 22µg** | | | **Rebif® 44µg** | | | **Copaxone®** | | | **Avonex®** | | | **Extavia®** | | |
| --- | --- | --- | --- | --- | --- | --- | --- | --- | --- | --- | --- | --- | --- | --- | --- | --- | --- | --- |
|  | **N** | **% (1)** | **% (2)** | **N** | **% (1)** | **% (2)** | **N** | **% (1)** | **% (2)** | **N** | **% (1)** | **% (2)** | **N** | **% (1)** | **% (2)** | **N** | **% (1)** | **% (2)** |
| **Less deterioration of my health** | 35 | 60.3 | 68.6 | 21 | 56.8 | 58.3 | 55 | 50.5 | 54.5 | 55 | 55.0 | 57.9 | 74 | 58.3 | 63.8 | 10 | 71.4 | 76.9 |
| **Simple administration** | 18 | 31.0 | 35.3 | 26 | 70.3 | 72.2 | 48 | 44.0 | 47.5 | 39 | 39.0 | 41.1 | 65 | 51.2 | 56.0 | 6 | 42.9 | 46.2 |
| **Safe administration** | 16 | 27.6 | 31.4 | 23 | 62.2 | 63.9 | 51 | 46.8 | 50.5 | 36 | 36.0 | 37.9 | 54 | 42.5 | 46.6 | 5 | 35.7 | 38.5 |
| **Improvement of my symptoms** | 20 | 34.5 | 39.2 | 14 | 37.8 | 38.9 | 50 | 45.9 | 49.5 | 42 | 42.0 | 44.2 | 51 | 40.2 | 44.0 | 5 | 35.7 | 38.5 |
| **Fewer relapses** | 21 | 36.2 | 41.2 | 15 | 40.5 | 41.7 | 44 | 40.4 | 43.6 | 38 | 38.0 | 40.0 | 51 | 40.2 | 44.0 | 5 | 35.7 | 38.5 |
| **Periodic administration** | 15 | 25.9 | 29.4 | 20 | 54.1 | 55.6 | 33 | 30.3 | 32.7 | 24 | 24.0 | 25.3 | 53 | 41.7 | 45.7 | 3 | 21.4 | 23.1 |
| **No inflammatory activity in MS** | 10 | 17.2 | 19.6 | 9 | 24.3 | 25.0 | 31 | 28.4 | 30.7 | 28 | 28.0 | 29.5 | 31 | 24.4 | 26.7 | 3 | 21.4 | 23.1 |
| **Patients with at least one advantage** | 51 | 87.9 | 100.0 | 36 | 97.3 | 100.0 | 101 | 92.7 | 100.0 | 95 | 95.0 | 100.0 | 116 | 91.3 | 100.0 | 13 | 92.9 | 100.0 |
| **Patients with no advantages** | 7 | 12.1 | - | 1 | 2.7 | - | 8 | 7.3 | - | 5 | 5.0 | - | 11 | 8.7 | - | 1 | 7.1 | - |
| **Total patients** | 58 | 100.0 | - | 37 | 100.0 | - | 109 | 100.0 | - | 100 | 100.0 | - | 127 | 100.0 | - | 14 | 100.0 | - |

^(1)^ Total percentage.

^(2)^ Valid percentage.

|  | | **N** | **% total ^(*)^** | **% valid ^(*)^** |
| --- | --- | --- | --- | --- |
| **For you personally, what are the disadvantages of the drugs you are currently taking or have taken for MS?** | **Problems in the injection site** | 243 | 54.6 | 66.8 |
|  | **Side effects in general** | 193 | 43.4 | 53.0 |
|  | **Expensive administration** | 85 | 19.1 | 23.4 |
|  | **Insufficient efficacy** | 59 | 13.3 | 16.2 |
| **Patients who indicated at least one disadvantage** | | 364 | 81.8 | 100.0 |
| **Patients who did not indicate any disadvantage** | | 81 | 18.2 | - |
| **Total patients** | | 445 | 100.0 | - |

^(*)^ Multiple response, so the sum of the percentages does not necessarily equal 100.

| **For you personally, what are the disadvantages of the drugs you are currently taking or have taken for MS?** | **Betaferon®** | | | **Rebif® 22µg** | | | **Rebif® 44µg** | | | **Copaxone®** | | | **Avonex®** | | | **Extavia®** | | |
| --- | --- | --- | --- | --- | --- | --- | --- | --- | --- | --- | --- | --- | --- | --- | --- | --- | --- | --- |
|  | **N** | **% (1)** | **% (2)** | **N** | **% (1)** | **% (2)** | **N** | **% (1)** | **% (2)** | **N** | **% (1)** | **% (2)** | **N** | **% (1)** | **% (2)** | **N** | **% (1)** | **% (2)** |
| **Problems in the injection site** | 40 | 69.0 | 78.4 | 16 | 43.2 | 59.3 | 69 | 63.3 | 74.2 | 67 | 67.0 | 84.8 | 46 | 36.2 | 44.7 | 5 | 35.7 | 45.5 |
| **Side effects in general** | 15 | 25.9 | 29.4 | 21 | 56.8 | 77.8 | 54 | 49.5 | 58.1 | 24 | 24.0 | 30.4 | 74 | 58.3 | 71.8 | 5 | 35.7 | 45.5 |
| **Expensive administration** | 15 | 25.9 | 29.4 | 2 | 5.4 | 7.4 | 19 | 17.4 | 20.4 | 18 | 18.0 | 22.8 | 27 | 21.3 | 26.2 | 4 | 28.6 | 36.4 |
| **Insufficient efficacy** | 9 | 15.5 | 17.6 | 4 | 10.8 | 14.8 | 10 | 9.2 | 10.8 | 9 | 9.0 | 11.4 | 23 | 18.1 | 22.3 | 4 | 28.6 | 36.4 |
| **Number of patients with at least one disadvantage** | 51 | 87.9 | 100.0 | 27 | 73.0 | 100.0 | 93 | 85.3 | 100.0 | 79 | 79.0 | 100.0 | 103 | 81.1 | 100.0 | 11 | 78.6 | 100.0 |
| **Patients with no disadvantages** | 7 | 12.1 | - | 10 | 27.0 | - | 16 | 14.7 | - | 21 | 21.0 | - | 24 | 18.9 | - | 3 | 21.4 | - |
| **Total patients** | 58 | 100.0 | - | 37 | 100.0 | - | 109 | 100.0 | - | 100 | 100.0 | - | 127 | 100.0 | - | 14 | 100.0 | - |

^(1)^ Total percentage.

^(2)^ Valid percentage.

|  | | **Have you ever previously discontinued a treatment or skipped an injection?** | | **Total** |
| --- | --- | --- | --- | --- |
|  |  | **No** | **Yes** |  |
| **Treatment** | **Betaferon®** | 34 (63.0%) | 20 (37.0%) | 54 (100.0%) |
|  | **Rebif® 22µg** | 21 (56.8%) | 16 (43.2%) | 37 (100.0%) |
|  | **Rebif® 44µg** | 70 (67.3%) | 34 (32.7%) | 104 (100.0%) |
|  | **Copaxone®** | 61 (62.2%) | 37 (37.8%) | 98 (100.0%) |
|  | **Avonex®** | 88 (72.7%) | 33 (27.3%) | 121 (100.0%) |
|  | **Extavia®** | 6 (42.9%) | 8 (57.1%) | 14 (100.0%) |
| **Total** | | 280 (65.4%) | 148 (34.6%) | 428 (100.0%) |

|  | | **N** | **% total ^(*)^** | **% valid ^(*)^** |
| --- | --- | --- | --- | --- |
| **Discontinuation for general reasons** | **Side effects in general** | 55 | 37.2 | 59.8 |
|  | **Problems in the injection site** | 45 | 30.4 | 48.9 |
|  | **Expensive administration** | 19 | 12.8 | 20.7 |
|  | **Insufficient efficacy** | 7 | 4.7 | 7.6 |
| **Patients who indicated at least one disadvantage** | | 92 | 62.2 | 100.0 |
| **Patients who did not indicate any disadvantage** | | 56 | 37.8 | - |
| **Total patients** | | 148 | 100.0 | - |

^(*)^ Multiple response, so the sum of the percentages does not necessarily equal 100.

| **Discontinuation for general reasons** | **Betaferon®** | | | **Rebif® 22µg** | | | **Rebif® 44µg** | | | **Copaxone®** | | | **Avonex®** | | | **Extavia®** | | |
| --- | --- | --- | --- | --- | --- | --- | --- | --- | --- | --- | --- | --- | --- | --- | --- | --- | --- | --- |
|  | **N** | **% (1)** | **% (2)** | **N** | **% (1)** | **% (2)** | **N** | **% (1)** | **% (2)** | **N** | **% (1)** | **% (2)** | **N** | **% (1)** | **% (2)** | **N** | **% (1)** | **% (2)** |
| **Side effects in general** | 6 | 30.0 | 66.7 | 6 | 37.5 | 66.7 | 8 | 23.5 | 44.4 | 18 | 48.6 | 75.0 | 15 | 45.5 | 55.6 | 2 | 25.0 | 40.0 |
| **Problems in the injection site** | 6 | 30.0 | 66.7 | 4 | 25.0 | 44.4 | 10 | 29.4 | 55.6 | 7 | 18.9 | 29.2 | 15 | 45.5 | 55.6 | 3 | 37.5 | 60.0 |
| **Expensive administration** | 1 | 5.0 | 11.1 | 2 | 12.5 | 22.2 | 4 | 11.8 | 22.2 | 3 | 8.1 | 12.5 | 8 | 24.2 | 29.6 | 1 | 12.5 | 20.0 |
| **Insufficient efficacy** | 1 | 5.0 | 11.1 | 1 | 6.3 | 11.1 | 1 | 2.9 | 5.6 | 2 | 5.4 | 8.3 | 2 | 6.1 | 7.4 | 0 | 0.0 | 0.0 |
| **Patients with at least one discontinuation** | 9 | 45.0 | 100.0 | 9 | 56.3 | 100.0 | 18 | 52.9 | 100.0 | 24 | 64.9 | 100.0 | 27 | 81.8 | 100.0 | 5 | 62.5 | 100.0 |
| **Patients with no discontinuations** | 11 | 55.0 | - | 7 | 43.8 | - | 16 | 47.1 | - | 13 | 35.1 | - | 6 | 18.2 | - | 3 | 37.5 | - |
| **Total patients** | 20 | 100.0 | - | 16 | 100.0 | - | 34 | 100.0 | - | 37 | 100.0 | - | 33 | 100.0 | - | 8 | 100.0 | - |

^(1)^ Total percentage. ^(2)^ Valid percentage.

|  | | **If yes, how often have you skipped an injection/tablet?** | | | | | | **Total** |
| --- | --- | --- | --- | --- | --- | --- | --- | --- |
|  |  | **Never in the last 3 months** | **Approx. once every 3 months** | **Approx. once a month** | **Approx. once a week** | **More than once a week** | **Never in the last 3 months, approx. once every 3 months** |  |
| **Treatment** | **Betaferon®** | 4 (22.2%) | 12 (66.7%) | 1 (5.6%) | 1 (5.6%) | 0 (0.0%) | 0 (0.0%) | 18 (100.0%) |
|  | **Rebif® 22µg** | 3 (18.8%) | 7 (43.8%) | 4 (25.0%) | 1 (6.3%) | 0 (0.0%) | 1 (6.3%) | 16 (100.0%) |
|  | **Rebif® 44µg** | 10 (32.3%) | 13 (41.9%) | 6 (19.4%) | 1 (3.2%) | 1 (3.2%) | 0 (0.0%) | 31 (100.0%) |
|  | **Copaxone®** | 12 (36.4%) | 11 (33.3%) | 6 (18.2%) | 2 (6.1%) | 2 (6.1%) | 0 (0.0%) | 33 (100.0%) |
|  | **Avonex®** | 14 (46.7%) | 14 (46.7%) | 1 (3.3%) | 1 (3.3%) | 0 (0.0%) | 0 (0.0%) | 30 (100.0%) |
|  | **Extavia®** | 3 (37.5%) | 2 (25.0%) | 2 (25.0%) | 1 (12.5%) | 0 (0.0%) | 0 (0.0%) | 8 (100.0%) |
| **Total** | | 46 (33.8%) | 59 (43.4%) | 20 (14.7%) | 7 (5.1%) | 3 (2.2%) | 1 (0.7%) | 136 (100.0%) |

# ANALYSIS OF OBJECTIVES

## Primary objective

**To measure patient satisfaction with immunomodulatory treatment for clinically isolated syndrome (CIS) and/or relapsing-remitting multiple sclerosis (RRMS)**

This objective was resolved in section 5.9.1 of this report.

## Secondary objective 1

**To determine the main factors affecting treatment satisfaction.**

Bivariate analyses were performed between the dimensions of the TSQM questionnaire and each of the variables described below:

- Drug
- Injection-related factors: Pain / Injection anxiety / Self-injected or needs help to self-inject / Local reactions / Interference with social life / Difficulty in preparation or administration
- Social factors: Occupation / Family status / Caregiver
- Economic factors: No of visits to PA for multiple sclerosis / No visits to hospitals for multiple sclerosis / Number of hospitalizations for multiple sclerosis / No of tests for multiple sclerosis
- No of trips for MS: By ambulance / By taxi
- Need to adapt: His/her home / His/her car / His/her workplace
- Receives rehabilitation
- Receives informal care
- No of drugs received for MS

Based on the bivariate analyses, variables with a p-value equal to or less than 0.200 are selected and entered in the multivariate analysis (multiple linear regression model).

As the TSQM satisfaction questionnaire has four dimensions, four different models were performed, one for each dimension.

### Dimension 1: Treatment effectiveness

| **DIMENSION 1** | | | | | |
| --- | --- | --- | --- | --- | --- |
| **Variable** | **B** | **Standard error** | **p-value** | **LCI for (B)** | **UCI for (B)** |
| **Drug** | 0.469 | 0.635 | 0.460 | -0.778 | 1.717 |
| **Pain** | -1.304 | 1.787 | 0.466 | -4.816 | 2.207 |
| **Injection anxiety** | -4.944 | 2.467 | 0.046 | -9.794 | -0.095 |
| **Self-injected or needs help to self-inject** | 1.557 | 2.509 | 0.535 | -3.374 | 6.488 |
| **Local reactions** | -0.926 | 1.785 | 0.604 | -4.434 | 2.582 |
| **Interference with social life** | -8.141 | 3.146 | 0.010 | -14.325 | -1.958 |
| **Difficulty in preparation or administration** | -1.152 | 4.620 | 0.803 | -10.232 | 7.929 |
| **Occupation** | -0.547 | 0.690 | 0.429 | -1.904 | 0.810 |
| **Family status** | 0.410 | 0.926 | 0.658 | -1.409 | 2.229 |
| **Caregiver?** | -3.855 | 2.933 | 0.189 | -9.620 | 1.910 |
| **Number of visits to primary care for multiple sclerosis** | -0.084 | 0.171 | 0.623 | -0.420 | 0.252 |
| **Number of visits to the hospital for multiple sclerosis** | 0.029 | 0.070 | 0.678 | -0.108 | 0.166 |
| **Number of hospitalizations for multiple sclerosis** | 0.179 | 1.333 | 0.894 | -2.443 | 2.800 |
| **Total number of tests/tests for multiple sclerosis** | 0.065 | 0.106 | 0.539 | -0.143 | 0.274 |
| **Number of ambulance trips for multiple sclerosis** | -8.261 | 4.543 | 0.070 | -17.193 | 0.670 |
| **Number of taxi trips for multiple sclerosis** | -2.504 | 0.986 | 0.012 | -4.443 | -0.565 |
| **Did home have to be adapted?** | -1.197 | 3.141 | 0.703 | -7.371 | 4.977 |
| **Did car have to adapted?** | -0.468 | 3.508 | 0.894 | -7.363 | 6.427 |
| **Did workplace have to be adapted?** | -2.861 | 2.410 | 0.236 | -7.598 | 1.875 |
| **Has he/she received rehabilitation?** | -6.850 | 2.273 | 0.003 | -11.317 | -2.383 |
| **Has he/she received informal care?** | -2.079 | 2.873 | 0.470 | -7.726 | 3.568 |
| **Number of pharmacological treatment received by the patient for MS** | -2.021 | 1.015 | 0.047 | -4.017 | -0.026 |

The multiple linear regression model was then obtained that allows us to establish statistically the possible relationship between dimension 1 of the TSQM questionnaire (dependent variable) and the independent variables that had a p-value equal to or less than 0.200 in the bivariate analysis, namely:

- Injection-related factors: Injection anxiety / Interference with social life
- Social factors: Caregiver
- No of trips for MS: By ambulance / By taxi
- Receives rehabilitation
- No of drugs received for MS

|  | **Nonstandardized coefficients** | | **p-value** | **95% CI for (B)** | |
| --- | --- | --- | --- | --- | --- |
|  | **B** | **Standard Error** |  | **Lower Limit** | **Upper Limit** |
| **(Constant)** | 69.039 | 1.048 | 0.000 | 66.978 | 71.100 |
| **Has he/she received rehabilitation?** | -10.330 | 2.920 | 0.000 | -16.073 | -4.588 |
| **Interference with social life** | -9.202 | 3.573 | 0.010 | -16.228 | -2.176 |

Dependent variable: Dimension 1 of TSQM questionnaire (Treatment effectiveness).

Receiving rehabilitation or having interference with social life reduces the score on 1 dimension of the TSQM questionnaire.

### Dimension 2: Adverse reactions

| **DIMENSION 2** | | | | | |
| --- | --- | --- | --- | --- | --- |
| **Variable** | **B** | **Standard error** | **p-value** | **LCI for (B)** | **UCI for (B)** |
| **Drug** | -2.098 | 0.811 | 0.010 | -3.691 | -0.504 |
| **Pain** | -5.132 | 2.283 | 0.025 | -9.619 | -0.645 |
| **Injection anxiety** | -8.125 | 3.138 | 0.010 | -14.292 | -1.958 |
| **Self-injected or needs help to self-inject** | -2.321 | 3.179 | 0.466 | -8.569 | 3.927 |
| **Local reactions** | -2.542 | 2.290 | 0.268 | -7.042 | 1.958 |
| **Interference with social life** | -14.359 | 4.009 | 0.000 | -22.239 | -6.480 |
| **Difficulty in preparation or administration** | -5.420 | 5.922 | 0.361 | -17.059 | 6.220 |
| **Occupation** | -0.953 | 0.886 | 0.282 | -2.693 | 0.787 |
| **Family status** | 0.556 | 1.190 | 0.640 | -1.783 | 2.895 |
| **Caregiver?** | -7.108 | 3.756 | 0.059 | -14.489 | 0.274 |
| **Number of visits to primary care for multiple sclerosis** | -0.353 | 0.212 | 0.096 | -0.770 | 0.064 |
| **Number of visits to the hospital for multiple sclerosis** | 0.003 | 0.091 | 0.977 | -0.176 | 0.181 |
| **Number of hospitalizations for multiple sclerosis** | 0.936 | 1.746 | 0.592 | -2.496 | 4.369 |
| **Total number of tests/tests for multiple sclerosis** | -0.149 | .137 | 0.275 | -0.418 | 0.119 |
| **Number of ambulance trips for multiple sclerosis** | -0.875 | 5.812 | 0.880 | -12.301 | 10.551 |
| **Number of taxi trips for multiple sclerosis** | -2.656 | 1.257 | 0.035 | -5.128 | -0.185 |
| **Did home have to be adapted?** | 2.273 | 4.030 | 0.573 | -5.647 | 10.193 |
| **Did car have to be adapted?** | 0.624 | 4.501 | 0.890 | -8.222 | 9.471 |
| **Did workplace have to adapted?** | 0.717 | 3.097 | 0.817 | -5.369 | 6.803 |
| **Has he/she received rehabilitation?** | -3.050 | 2.943 | 0.301 | -8.835 | 2.734 |
| **Has he/she received informal care?** | -2.360 | 3.687 | 0.522 | -9.606 | 4.886 |
| **Number of pharmacological treatment received by the patient for MS** | -2.625 | 1.318 | 0.047 | -5.217 | -0.034 |

The multiple linear regression model was then obtained that allows us to establish statistically the possible relationship between dimension 2 of the TSQM questionnaire (dependent variable) and the independent variables that had a p-value equal to or less than 0.200 in the bivariate analysis, namely:

- Drug
- Injection-related factors: Pain / Injection anxiety / Interference with social life
- Social factors: Caregiver
- Economic factors: Number of visits to primary care for multiple sclerosis
- No of trips for MS: By ambulance / By taxi
- No of drugs received for MS

|  | **Nonstandardized coefficients** | | **p-value** | **95% CI for (B)** | |
| --- | --- | --- | --- | --- | --- |
|  | **B** | **Standard Error** |  | **Lower Limit** | **Upper Limit** |
| **(Constant)** | 74.580 | 1.466 | 0.000 | 71.694 | 77.466 |
| **Interference with social life** | -14.461 | 5.147 | 0.005 | -24.597 | -4.324 |

Dependent variable: Dimension 2 of TSQM questionnaire (Adverse reactions).

Having interference with social life reduces he score on dimension 2 of the TSQM questionnaire.

### Dimension 3: Convenience

| **DIMENSION 3** | | | | | |
| --- | --- | --- | --- | --- | --- |
| **Variable** | **B** | **Standard error** | **p-value** | **LCI for (B)** | **UCI for (B)** |
| **Drug** | 0.795 | 0.651 | 0.223 | -0.485 | 2.075 |
| **Pain** | -4.141 | 1.822 | 0.024 | -7.722 | -0.559 |
| **Injection anxiety** | -5.125 | 2.522 | 0.043 | -10.081 | -0.169 |
| **Self-injected or needs help to self-inject** | -1.308 | 2.548 | 0.608 | -6.316 | 3.700 |
| **Local reactions** | -2.489 | 1.827 | 0.174 | -6.079 | 1.101 |
| **Interference with social life** | -13.593 | 3.157 | 0.000 | -19.797 | -7.388 |
| **Difficulty in preparation or administration** | -10.627 | 4.726 | 0.025 | -19.916 | -1.339 |
| **Occupation** | -0.430 | 0.708 | 0.544 | -1.821 | 0.961 |
| **Family status** | -0.462 | 0.951 | 0.627 | -2.331 | 1.407 |
| **Caregiver?** | -7.169 | 3.003 | 0.017 | -13.071 | -1.266 |
| **Number of visits to primary care for multiple sclerosis** | -0.172 | 0.169 | 0.311 | -0.505 | 0.161 |
| **Number of visits to the hospital for multiple sclerosis** | 0.025 | 0.072 | 0.727 | -0.116 | 0.167 |
| **Number of hospitalizations for multiple sclerosis** | -1.250 | 1.376 | 0.364 | -3.955 | 1.455 |
| **Total number of tests/tests for multiple sclerosis** | 0.032 | 0.109 | 0.772 | -0.183 | 0.247 |
| **Number of ambulance trips for multiple sclerosis** | -3.600 | 4.651 | 0.439 | -12.743 | 5.543 |
| **Number of taxi trips for multiple sclerosis** | -1.094 | 1.011 | 0.280 | -3.082 | 0.894 |
| **Did home have to be adapted?** | -0.345 | 3.232 | 0.915 | -6.697 | 6.008 |
| **Did car have to be adapted?** | -2.013 | 3.608 | 0.577 | -9.104 | 5.079 |
| **Did workplace have to be adapted?** | -0.845 | 2.483 | 0.734 | -5.725 | 4.034 |
| **Has he/she received rehabilitation?** | -2.497 | 2.358 | 0.290 | -7.132 | 2.138 |
| **Has he/she received informal care?** | -4.372 | 2.950 | 0.139 | -10.169 | 1.426 |
| **Number of pharmacological treatment received by the patient for MS** | -0.758 | 1.042 | 0.467 | -2.806 | 1.289 |

The multiple linear regression model was then obtained that allows us to establish statistically the possible relationship between dimension 3 of the TSQM questionnaire (dependent variable) and the independent variables that had a p-value equal to or less than 0.200 in the bivariate analysis, namely:

- Injection-related factors: Pain / Injection anxiety / Local reactions / Interference with social life / Difficulty in preparation or administration
- Social factors: Caregiver
- Receives informal care

|  | **Nonstandardized coefficients** | | **p-value** | **95% CI for (B)** | |
| --- | --- | --- | --- | --- | --- |
|  | **B** | **Standard Error** |  | **Lower Limit** | **Upper Limit** |
| **(Constant)** | 66.306 | 1.297 | 0.000 | 63.757 | 68.856 |
| **Interference with social life** | -12.743 | 3.126 | 0.000 | -18.888 | -6.599 |
| **Difficulty in preparation or administration** | -8.488 | 4.664 | 0.069 | -17.655 | 0.678 |
| **Pain** | -4.325 | 1.792 | 0.016 | -7.847 | -0.803 |
| **Caregiver?** | -6.581 | 2.997 | 0.029 | -12.473 | -0.690 |

Dependent variable: Dimension 3 of TSQM questionnaire (Convenience).

Having pain, interference with social life, difficulty in preparation or administration or having a caregiver reduces the score on dimension 3 of the questionnaire.

### Dimension 4: Overall satisfaction

| **DIMENSION 4** | | | | | |
| --- | --- | --- | --- | --- | --- |
| **Variable** | **B** | **Standard error** | **p-value** | **LCI for (B)** | **UCI for (B)** |
| **Drug** | -0.031 | 0.633 | 0.961 | -1.275 | 1.213 |
| **Pain** | -5.852 | 1.755 | 0.001 | -9.302 | -2.403 |
| **Injection anxiety** | -0.984 | 2.458 | 0.689 | -5.815 | 3.846 |
| **Self-injected or needs help to self-inject** | -1.646 | 2.472 | 0.506 | -6.504 | 3.212 |
| **Local reactions** | -2.600 | 1.770 | 0.143 | -6.078 | 0.879 |
| **Interference with social life** | -14.585 | 3.049 | 0.000 | -20.578 | -8.592 |
| **Difficulty in preparation or administration** | -3.816 | 4.609 | 0.408 | -12.875 | 5.243 |
| **Occupation** | -0.763 | 0.686 | 0.267 | -2.110 | 0.585 |
| **Family status** | 0.122 | 0.923 | 0.895 | -1.692 | 1.935 |
| **Caregiver?** | -8.112 | 2.908 | 0.006 | -13.826 | -2.397 |
| **Number of visits to primary care for multiple sclerosis** | -0.110 | 0.173 | 0.526 | -0.451 | 0.231 |
| **Number of visits to the hospital for multiple sclerosis** | -0.078 | 0.070 | 0.267 | -0.215 | 0.060 |
| **Number of hospitalizations for multiple sclerosis** | -0.069 | 1.375 | 0.960 | -2.773 | 2.634 |
| **Total number of tests/tests for multiple sclerosis** | -0.054 | 0.107 | 0.615 | -0.265 | 0.157 |
| **Number of ambulance trips for multiple sclerosis** | -4.998 | 4.550 | 0.273 | -13.944 | 3.948 |
| **Number of taxi trips for multiple sclerosis** | -2.632 | 0.988 | 0.008 | -4.575 | -0.690 |
| **Did home have to be adapted?** | 3.223 | 3.133 | 0.304 | -2.934 | 9.380 |
| **Did car have to be adapted?** | 1.579 | 3.502 | 0.652 | -5.304 | 8.462 |
| **Did workplace have to be adapted?** | 1.972 | 2.408 | 0.413 | -2.760 | 6.704 |
| **Has he/she received rehabilitation?** | -5.410 | 2.265 | 0.017 | -9.861 | -0.958 |
| **Has he/she received informal care?** | -0.439 | 2.870 | 0.878 | -6.079 | 5.201 |
| **Number of pharmacological treatment received by the patient for MS** | -3.749 | 1.014 | 0.000 | -5.741 | -1.756 |

The multiple linear regression model was then obtained that allows us to establish statistically the possible relationship between dimension 4 of the TSQM questionnaire (dependent variable) and the independent variables that had a p-value equal to or less than 0.200 in the bivariate analysis, namely:

- Injection-related factors: Pain / Local reactions / Interference with social life
- Social factors: Caregiver
- No of trips for MS: By taxi
- Receives rehabilitation
- No of drugs received for MS

|  | **Nonstandardized coefficients** | | **p-value** | **95% CI for (B)** | |
| --- | --- | --- | --- | --- | --- |
|  | **B** | **Standard Error** |  | **Lower Limit** | **Upper Limit** |
| **(Constant)** | 77.059 | 1.788 | 0.000 | 73.542 | 80.576 |
| **Interference with social life** | -15.280 | 3.427 | 0.000 | -22.019 | -8.540 |
| **Number of pharmacological treatment received by the patient for MS** | -3.155 | 1.077 | 0.004 | -5.272 | -1.038 |
| **Has he/she received rehabilitation?** | -8.296 | 2.932 | 0.005 | -14.062 | -2.531 |
| **Pain** | -4.410 | 1.881 | 0.020 | -8.110 | -0.710 |

Dependent variable: Dimension 4 of TSQM questionnaire (Overall satisfaction).

Receiving rehabilitation, having pain or interference with social life reduces the score on dimension 4 of the TSQM questionnaire. In addition, the higher the number of pharmacological treatments for MS, the lower the overall satisfaction.

## Secondary objective 2

**To evaluate the impact of local/systemic reactions related to parenteral administration on treatment satisfaction.**

A variable with two categories was created:

- **At least 1 parenterally-related AR:** When the patient has had at least one adverse reaction related to parenteral administration.
- **No parenterally-related AR:** When the patient has not had any adverse reaction related to parenteral administration or when the patient has not had adverse reactions.

The dimensions of the TSQM questionnaire according to the new variable created are described below.

| **Dimension 1 of TSQM: Effectiveness** | **Mean** | **SD** | **Median** | **Minimum** | **Maximum** | **Q1** | **Q3** | **N** |
| --- | --- | --- | --- | --- | --- | --- | --- | --- |
| **No parenterally-related AR** | 67.9 | 18.6 | 66.7 | 0.0 | 100.0 | 61.1 | 83.3 | 314 |
| **At least 1 parenterally-related AR** | 64.1 | 18.7 | 66.7 | 0.0 | 100.0 | 55.6 | 77.8 | 125 |
| **Total** | 66.8 | 18.7 | 66.7 | 0.0 | 100.0 | 55.6 | 77.8 | 439 |
| **p-value** | **0.127 ^(*)^** | | | | | | | |

^(*)^ Mann-Whitney.

| **Dimension 2 of TSQM: Adverse reactions** | **Mean** | **SD** | **Median** | **Minimum** | **Maximum** | **Q1** | **Q3** | **N** |
| --- | --- | --- | --- | --- | --- | --- | --- | --- |
| **No parenterally-related AR** | 72.0 | 24.2 | 68.8 | 12.5 | 100.0 | 50.0 | 100.0 | 314 |
| **At least 1 parenterally-related AR** | 73.8 | 23.2 | 75.0 | 25.0 | 100.0 | 56.3 | 100.0 | 124 |
| **Total** | 72.5 | 23.9 | 68.8 | 12.5 | 100.0 | 50.0 | 100.0 | 438 |
| **p-value** | **0.393 ^(*)^** | | | | | | | |

^(*)^ Mann-Whitney.

| **Dimension 3 of TSQM: Convenience** | **Mean** | **SD** | **Median** | **Minimum** | **Maximum** | **Q1** | **Q3** | **N** |
| --- | --- | --- | --- | --- | --- | --- | --- | --- |
| **No parenterally-related AR** | 61.4 | 19.6 | 66.7 | 0.0 | 100.0 | 50.0 | 75.0 | 317 |
| **At least 1 parenterally-related AR** | 64.1 | 18.0 | 66.7 | 16.7 | 100.0 | 50.0 | 75.0 | 125 |
| **Total** | 62.2 | 19.2 | 66.7 | 0.0 | 100.0 | 50.0 | 75.0 | 442 |
| **p-value** | **0.115 ^(*)^** | | | | | | | |

^(*)^ Mann-Whitney.

| **Dimension 4 of TSQM: Overall satisfaction** | **Mean** | **SD** | **Median** | **Minimum** | **Maximum** | **Q1** | **Q3** | **N** |
| --- | --- | --- | --- | --- | --- | --- | --- | --- |
| **No parenterally-related AR** | 69.4 | 18.5 | 71.4 | 0.0 | 100.0 | 57.1 | 78.6 | 318 |
| **At least 1 parenterally-related AR** | 67.3 | 19.0 | 71.4 | 0.0 | 100.0 | 53.6 | 78.6 | 125 |
| **Total** | 68.8 | 18.6 | 71.4 | 0.0 | 100.0 | 57.1 | 78.6 | 443 |
| **p-value** | **0.321 ^(*)^** | | | | | | | |

^(*)^ Mann-Whitney.

## Secondary objective 3

**To evaluate the impact of satisfaction with immunomodulatory therapies on patient treatment adherence.**

This objective cannot be resolved because no information was collected on adherence in the CRF.

## Secondary objective 4

**To evaluate the effect of treatment satisfaction and adherence on the results of disease activity (EDSS and relapses).**

The relationship between adherence and disease activity cannot be performed because no information was collected on adherence in the CRF.

The relationship between satisfaction and disease activity is described below:

### Current EDSS score


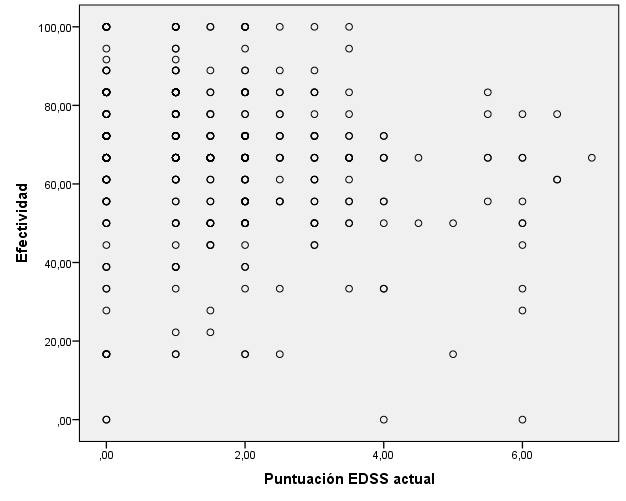


|  | **Current EDSS score** | | |
| --- | --- | --- | --- |
|  | **N** | **Correlation coefficient ^(*)^** | **p-value** |
| **Dimension 1: Effectiveness** | 434 | -0.171 | 0.000 |

^(*)^ Spearman.


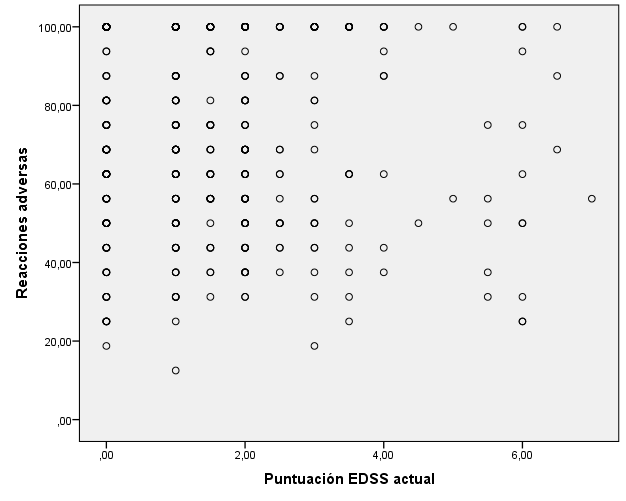


|  | **Current EDSS score** | | |
| --- | --- | --- | --- |
|  | **N** | **Correlation coefficient ^(*)^** | **p-value** |
| **Dimension 2: Adverse reactions** | 433 | -0.031 | 0.514 |

^(*)^ Spearman.


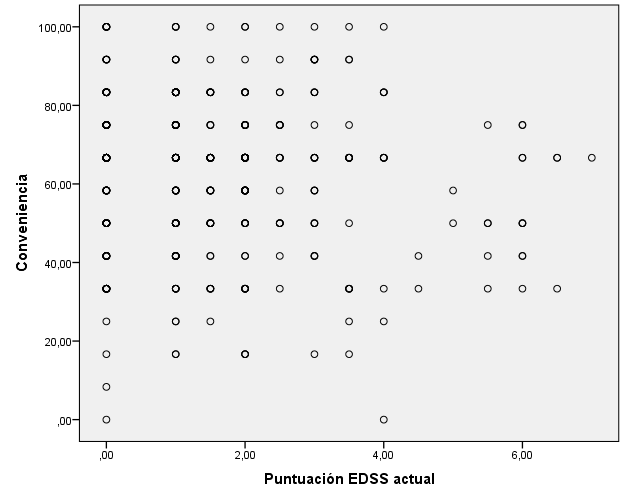


|  | **Current EDSS score** | | |
| --- | --- | --- | --- |
|  | **N** | **Correlation coefficient ^(*)^** | **p-value** |
| **Dimension 3: Convenience** | 437 | -0.032 | 0.506 |

^(*)^ Spearman.


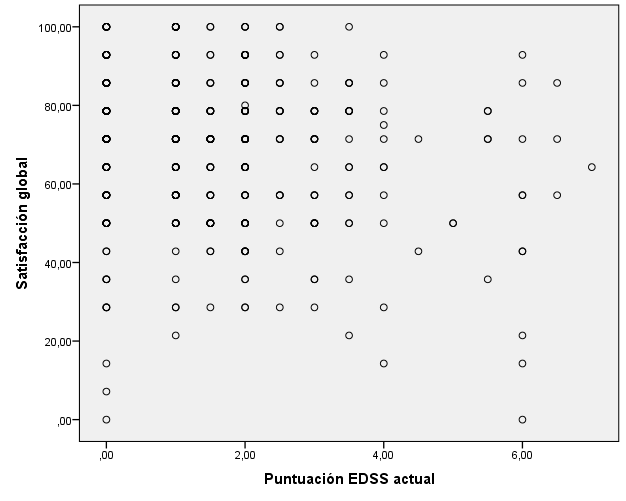


|  | **Current EDSS score** | | |
| --- | --- | --- | --- |
|  | **N** | **Correlation coefficient ^(*)^** | **p-value** |
| **Dimension 4: Overall satisfaction** | 438 | -0.110 | 0.021 |

^(*)^ Spearman.

### Number of relapses in the last year


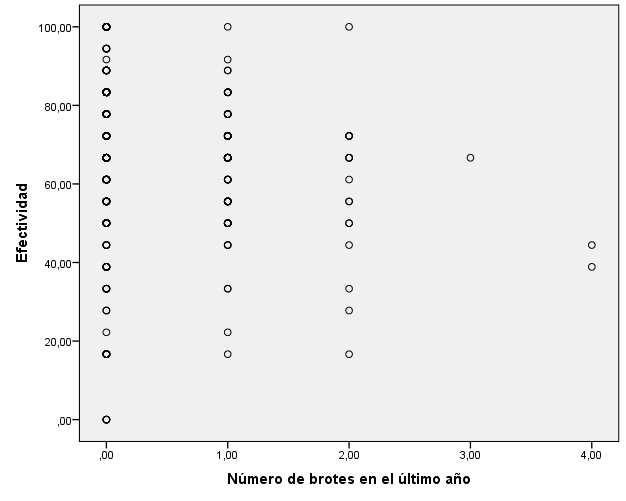


|  | **Number of relapses in the last year** | | |
| --- | --- | --- | --- |
|  | **N** | **Correlation coefficient ^(*)^** | **p-value** |
| **Dimension 1: Effectiveness** | 438 | -0.169 | 0.000 |

^(*)^ Spearman.


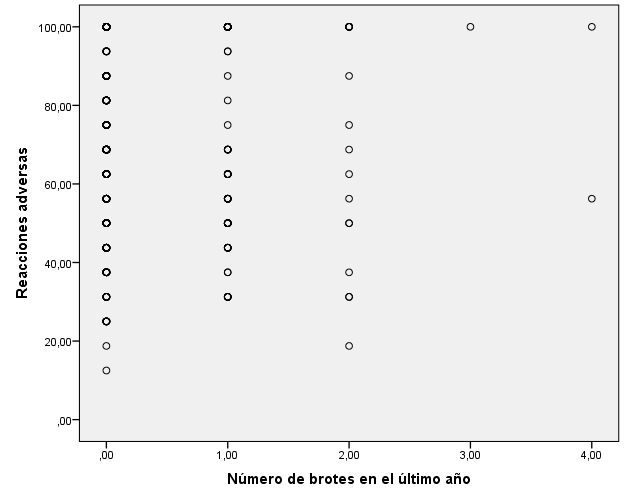


|  | **Number of relapses in the last year** | | |
| --- | --- | --- | --- |
|  | **N** | **Correlation coefficient ^(*)^** | **p-value** |
| **Dimension 2: Adverse reactions** | 438 | -0.002 | 0.967 |

^(*)^ Spearman.


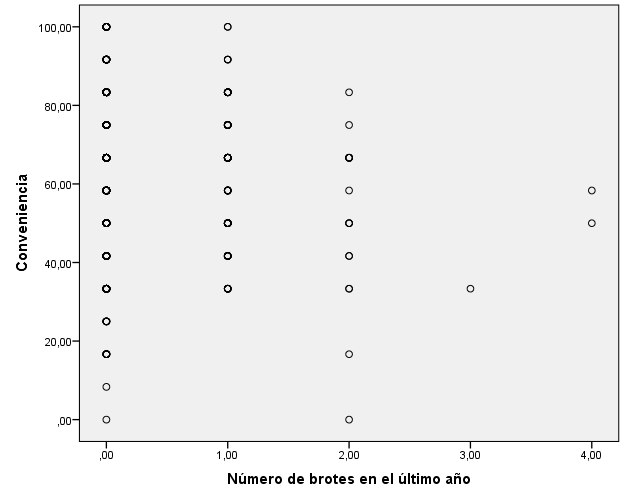


|  | **Number of relapses in the last year** | | |
| --- | --- | --- | --- |
|  | **N** | **Correlation coefficient ^(*)^** | **p-value** |
| **Dimension 3: Convenience** | 441 | -0.068 | 0.153 |

^(*)^ Spearman.


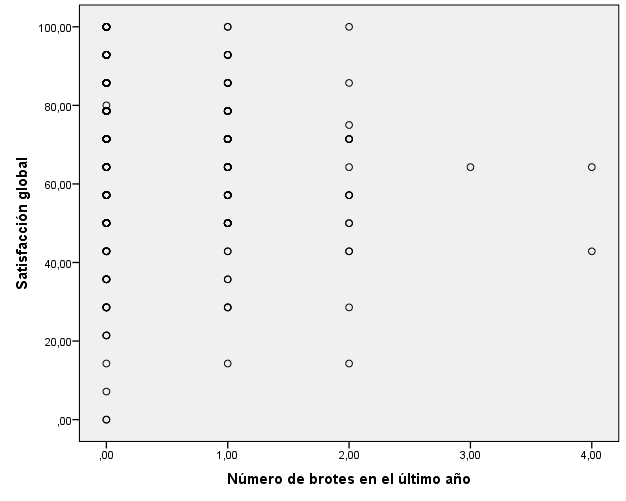


|  | **Number of relapses in the last year** | | |
| --- | --- | --- | --- |
|  | **N** | **Correlation coefficient ^(*)^** | **p-value** |
| **Dimension 4: Overall satisfaction** | 442 | -0.186 | 0.000 |

^(*)^ Spearman.

## Secondary objective 5

**To determine the effect of treatment satisfaction and adherence on the costs associated with disease management.**

The relationship between adherence and resources associated with disease management cannot be performed because no information was collected on adherence in the CRF.

The relationship between satisfaction and resources associated with disease management is described below:

### Dimension 1: Treatment effectiveness

| **Number of visits to primary care for multiple sclerosis** | **Dimension 1 of TSQM: Effectiveness** | | | | | | | |
| --- | --- | --- | --- | --- | --- | --- | --- | --- |
|  | **Mean** | **SD** | **Median** | **Minimum** | **Maximum** | **Q1** | **Q3** | **N** |
| **None** | 67.0 | 18.3 | 66.7 | 16.7 | 100.0 | 55.6 | 77.8 | 202 |
| **At least one** | 65.6 | 19.0 | 66.7 | 16.7 | 100.0 | 55.6 | 77.8 | 80 |
| **Total** | 66.6 | 18.4 | 66.7 | 16.7 | 100.0 | 55.6 | 77.8 | 282 |
| **p-value** | **0.339 ^(*)^** | | | | | | | |

^(*)^ Mann-Whitney.

| **Number of visits to the hospital for multiple sclerosis** | **Dimension 1 of TSQM: Effectiveness** | | | | | | | |
| --- | --- | --- | --- | --- | --- | --- | --- | --- |
|  | **Mean** | **SD** | **Median** | **Minimum** | **Maximum** | **Q1** | **Q3** | **N** |
| **None** | 68.8 | 15.8 | 66.7 | 16.7 | 100.0 | 66.7 | 77.8 | 29 |
| **At least one** | 66.9 | 18.6 | 66.7 | 0.0 | 100.0 | 55.6 | 77.8 | 361 |
| **Total** | 67.1 | 18.4 | 66.7 | 0.0 | 100.0 | 59.7 | 77.8 | 390 |
| **p-value** | **0.461 ^(*)^** | | | | | | | |

^(*)^ Mann-Whitney.

| **Number of hospitalizations for multiple sclerosis** | **Dimension 1 of TSQM: Effectiveness** | | | | | | | |
| --- | --- | --- | --- | --- | --- | --- | --- | --- |
|  | **Mean** | **SD** | **Median** | **Minimum** | **Maximum** | **Q1** | **Q3** | **N** |
| **None** | 67.3 | 18.5 | 66.7 | 0.0 | 100.0 | 61.1 | 77.8 | 371 |
| **At least one** | 66.3 | 16.1 | 66.7 | 33.3 | 94.4 | 50.0 | 77.8 | 31 |
| **Total** | 67.2 | 18.3 | 66.7 | 0.0 | 100.0 | 61.1 | 77.8 | 402 |
| **p-value** | **0.498 ^(*)^** | | | | | | | |

^(*)^ Mann-Whitney.

| **Total number of tests/tests for multiple sclerosis** | **Dimension 1 of TSQM: Effectiveness** | | | | | | | |
| --- | --- | --- | --- | --- | --- | --- | --- | --- |
|  | **Mean** | **SD** | **Median** | **Minimum** | **Maximum** | **Q1** | **Q3** | **N** |
| **None** | 61.1 | 20.0 | 61.1 | 33.3 | 100.0 | 44.4 | 72.2 | 9 |
| **At least one** | 67.1 | 18.5 | 66.7 | 0.0 | 100.0 | 61.1 | 77.8 | 378 |
| **Total** | 66.9 | 18.5 | 66.7 | 0.0 | 100.0 | 55.6 | 77.8 | 387 |
| **p-value** | **0.227 ^(*)^** | | | | | | | |

^(*)^ Mann-Whitney.

| **Number of ambulance trips for multiple sclerosis** | **Dimension 1 of TSQM: Effectiveness** | | | | | | | |
| --- | --- | --- | --- | --- | --- | --- | --- | --- |
|  | **Mean** | **SD** | **Median** | **Minimum** | **Maximum** | **Q1** | **Q3** | **N** |
| **None** | 67.0 | 18.7 | 66.7 | 0.0 | 100.0 | 55.6 | 77.8 | 390 |
| **At least one** | 47.2 | 19.6 | 47.2 | 33.3 | 61.1 | 33.3 | - | 2 |
| **Total** | 66.9 | 18.7 | 66.7 | 0.0 | 100.0 | 55.6 | 77.8 | 392 |
| **p-value** | **0.105 ^(*)^** | | | | | | | |

^(*)^ Mann-Whitney.

| **Number of taxi trips for multiple sclerosis** | **Dimension 1 of TSQM: Effectiveness** | | | | | | | |
| --- | --- | --- | --- | --- | --- | --- | --- | --- |
|  | **Mean** | **SD** | **Median** | **Minimum** | **Maximum** | **Q1** | **Q3** | **N** |
| **None** | 67.2 | 18.7 | 66.7 | 0.0 | 100.0 | 56.9 | 77.8 | 368 |
| **At least one** | 51.9 | 19.1 | 58.3 | 16.7 | 66.7 | 37.5 | 66.7 | 6 |
| **Total** | 66.9 | 18.8 | 66.7 | 0.0 | 100.0 | 55.6 | 77.8 | 374 |
| **p-value** | **<0.05 ^(*)^** | | | | | | | |

^(*)^ Mann-Whitney.

| **Did home have to be adapted?** | **Dimension 1 of TSQM: Effectiveness** | | | | | | | |
| --- | --- | --- | --- | --- | --- | --- | --- | --- |
|  | **Mean** | **SD** | **Median** | **Minimum** | **Maximum** | **Q1** | **Q3** | **N** |
| **No** | 67.0 | 18.6 | 66.7 | 0.0 | 100.0 | 61.1 | 77.8 | 417 |
| **Yes** | 60.5 | 21.4 | 61.1 | 16.7 | 100.0 | 50.0 | 77.8 | 17 |
| **Total** | 66.7 | 18.7 | 66.7 | 0.0 | 100.0 | 55.6 | 77.8 | 434 |
| **p-value** | **0.144 ^(*)^** | | | | | | | |

^(*)^ Mann-Whitney.

| **Did car have to be adapted?** | **Dimension 1 of TSQM: Effectiveness** | | | | | | | |
| --- | --- | --- | --- | --- | --- | --- | --- | --- |
|  | **Mean** | **SD** | **Median** | **Minimum** | **Maximum** | **Q1** | **Q3** | **N** |
| **No** | 66.8 | 18.7 | 66.7 | 0.0 | 100.0 | 56.9 | 77.8 | 428 |
| **Yes** | 60.0 | 21.3 | 66.7 | 27.8 | 77.8 | 38.9 | 77.8 | 5 |
| **Total** | 66.8 | 18.7 | 66.7 | 0.0 | 100.0 | 55.6 | 77.8 | 433 |
| **p-value** | **0.528 ^(*)^** | | | | | | | |

^(*)^ Mann-Whitney.

| **Did workplace have to be adapted?** | **Dimension 1 of TSQM: Effectiveness** | | | | | | | |
| --- | --- | --- | --- | --- | --- | --- | --- | --- |
|  | **Mean** | **SD** | **Median** | **Minimum** | **Maximum** | **Q1** | **Q3** | **N** |
| **No** | 67.1 | 18.6 | 66.7 | 0.0 | 100.0 | 61.1 | 77.8 | 415 |
| **Yes** | 56.6 | 24.9 | 66.7 | 0.0 | 77.8 | 55.6 | 66.7 | 11 |
| **Total** | 66.9 | 18.8 | 66.7 | 0.0 | 100.0 | 59.7 | 77.8 | 426 |
| **p-value** | **0.162 ^(*)^** | | | | | | | |

^(*)^ Mann-Whitney.

| **Has he/she received rehabilitation?** | **Dimension 1 of TSQM: Effectiveness** | | | | | | | |
| --- | --- | --- | --- | --- | --- | --- | --- | --- |
|  | **Mean** | **SD** | **Median** | **Minimum** | **Maximum** | **Q1** | **Q3** | **N** |
| **No** | 68.1 | 18.3 | 66.7 | 0.0 | 100.0 | 61.1 | 83.3 | 374 |
| **Yes** | 58.7 | 19.2 | 66.7 | 0.0 | 100.0 | 50.0 | 69.4 | 61 |
| **Total** | 66.7 | 18.7 | 66.7 | 0.0 | 100.0 | 55.6 | 77.8 | 435 |
| **p-value** | **<0.001 ^(*)^** | | | | | | | |

^(*)^ Mann-Whitney.

| **Has he/she received informal care?** | **Dimension 1 of TSQM: Effectiveness** | | | | | | | |
| --- | --- | --- | --- | --- | --- | --- | --- | --- |
|  | **Mean** | **SD** | **Median** | **Minimum** | **Maximum** | **Q1** | **Q3** | **N** |
| **No** | 67.1 | 18.9 | 66.7 | 0.0 | 100.0 | 61.1 | 77.8 | 409 |
| **Yes** | 61.6 | 15.1 | 61.1 | 27.8 | 88.9 | 50.0 | 69.4 | 25 |
| **Total** | 66.7 | 18.7 | 66.7 | 0.0 | 100.0 | 55.6 | 77.8 | 434 |
| **p-value** | **<0.05 ^(*)^** | | | | | | | |

^(*)^ Mann-Whitney.

| **Number of pharmacological treatment received per patient for multiple sclerosis** | **Dimension 1 of TSQM: Effectiveness** | | | | | | | |
| --- | --- | --- | --- | --- | --- | --- | --- | --- |
|  | **Mean** | **SD** | **Median** | **Minimum** | **Maximum** | **Q1** | **Q3** | **N** |
| **None** | 67.9 | 21.8 | 66.7 | 0.0 | 100.0 | 61.1 | 83.3 | 41 |
| **At least one** | 66.9 | 18.1 | 66.7 | 0.0 | 100.0 | 55.6 | 77.8 | 378 |
| **Total** | 67.0 | 18.4 | 66.7 | 0.0 | 100.0 | 55.6 | 77.8 | 419 |
| **p-value** | **0.519 ^(*)^** | | | | | | | |

^(*)^ Mann-Whitney.

### Dimension 2: Adverse reactions

| **Number of visits to primary care for multiple sclerosis** | **Dimension 2 of TSQM: Adverse reactions** | | | | | | | |
| --- | --- | --- | --- | --- | --- | --- | --- | --- |
|  | **Mean** | **SD** | **Median** | **Minimum** | **Maximum** | **Q1** | **Q3** | **N** |
| **None** | 73.0 | 22.5 | 75.0 | 25.0 | 100.0 | 56.3 | 100.0 | 201 |
| **At least one** | 72.7 | 24.1 | 68.8 | 25.0 | 100.0 | 56.3 | 100.0 | 83 |
| **Total** | 72.9 | 23.0 | 68.8 | 25.0 | 100.0 | 56.3 | 100.0 | 284 |
| **p-value** | **0.973 ^(*)^** | | | | | | | |

^(*)^ Mann-Whitney.

| **Number of visits to the hospital for multiple sclerosis** | **Dimension 2 of TSQM: Adverse reactions** | | | | | | | |
| --- | --- | --- | --- | --- | --- | --- | --- | --- |
|  | **Mean** | **SD** | **Median** | **Minimum** | **Maximum** | **Q1** | **Q3** | **N** |
| **None** | 73.7 | 24.2 | 68.8 | 31.3 | 100.0 | 53.1 | 100.0 | 29 |
| **At least one** | 73.3 | 24.0 | 75.0 | 12.5 | 100.0 | 56.3 | 100.0 | 359 |
| **Total** | 73.3 | 24.0 | 75.0 | 12.5 | 100.0 | 56.3 | 100.0 | 388 |
| **p-value** | **0.992 ^(*)^** | | | | | | | |

^(*)^ Mann-Whitney.

| **Number of hospitalizations for multiple sclerosis** | **Dimension 2 of TSQM: Adverse reactions** | | | | | | | |
| --- | --- | --- | --- | --- | --- | --- | --- | --- |
|  | **Mean** | **SD** | **Median** | **Minimum** | **Maximum** | **Q1** | **Q3** | **N** |
| **None** | 73.2 | 24.0 | 75.0 | 12.5 | 100.0 | 56.3 | 100.0 | 371 |
| **At least one** | 73.8 | 24.2 | 68.8 | 31.3 | 100.0 | 50.0 | 100.0 | 31 |
| **Total** | 73.3 | 24.0 | 75.0 | 12.5 | 100.0 | 56.3 | 100.0 | 402 |
| **p-value** | **0.929 ^(*)^** | | | | | | | |

^(*)^ Mann-Whitney.

| **Total number of tests/tests for multiple sclerosis** | **Dimension 2 of TSQM: Adverse reactions** | | | | | | | |
| --- | --- | --- | --- | --- | --- | --- | --- | --- |
|  | **Mean** | **SD** | **Median** | **Minimum** | **Maximum** | **Q1** | **Q3** | **N** |
| **None** | 72.9 | 20.0 | 68.8 | 50.0 | 100.0 | 53.1 | 93.8 | 9 |
| **At least one** | 73.4 | 24.0 | 75.0 | 12.5 | 100.0 | 56.3 | 100.0 | 376 |
| **Total** | 73.4 | 23.9 | 75.0 | 12.5 | 100.0 | 56.3 | 100.0 | 385 |
| **p-value** | **0.846 ^(*)^** | | | | | | | |

^(*)^ Mann-Whitney.

| **Number of ambulance trips for multiple sclerosis** | **Dimension 2 of TSQM: Adverse reactions** | | | | | | | |
| --- | --- | --- | --- | --- | --- | --- | --- | --- |
|  | **Mean** | **SD** | **Median** | **Minimum** | **Maximum** | **Q1** | **Q3** | **N** |
| **None** | 73.0 | 23.9 | 68.8 | 12.5 | 100.0 | 50.0 | 100.0 | 391 |
| **At least one** | 62.5 | 17.7 | 62.5 | 50.0 | 75.0 | 50.0 | - | 2 |
| **Total** | 73.0 | 23.9 | 68.8 | 12.5 | 100.0 | 50.0 | 100.0 | 393 |
| **p-value** | **0.504 ^(*)^** | | | | | | | |

^(*)^ Mann-Whitney.

| **Number of taxi trips for multiple sclerosis** | **Dimension 2 of TSQM: Adverse reactions** | | | | | | | |
| --- | --- | --- | --- | --- | --- | --- | --- | --- |
|  | **Mean** | **SD** | **Median** | **Minimum** | **Maximum** | **Q1** | **Q3** | **N** |
| **None** | 73.4 | 23.8 | 75.0 | 12.5 | 100.0 | 56.3 | 100.0 | 369 |
| **At least one** | 57.3 | 24.5 | 53.1 | 25.0 | 100.0 | 43.8 | 71.9 | 6 |
| **Total** | 73.2 | 23.8 | 75.0 | 12.5 | 100.0 | 56.3 | 100.0 | 375 |
| **p-value** | **0.109 ^(*)^** | | | | | | | |

^(*)^ Mann-Whitney.

| **Did home have to be adapted?** | **Dimension 2 of TSQM: Adverse reactions** | | | | | | | |
| --- | --- | --- | --- | --- | --- | --- | --- | --- |
|  | **Mean** | **SD** | **Median** | **Minimum** | **Maximum** | **Q1** | **Q3** | **N** |
| **No** | 72.4 | 24.1 | 68.8 | 12.5 | 100.0 | 50.0 | 100.0 | 416 |
| **Yes** | 74.3 | 21.4 | 75.0 | 31.3 | 100.0 | 56.3 | 96.9 | 17 |
| **Total** | 72.4 | 23.9 | 68.8 | 12.5 | 100.0 | 50.0 | 100.0 | 433 |
| **p-value** | **0.854 ^(*)^** | | | | | | | |

^(*)^ Mann-Whitney.

| **Did car have to be adapted?** | **Dimension 2 of TSQM: Adverse reactions** | | | | | | | |
| --- | --- | --- | --- | --- | --- | --- | --- | --- |
|  | **Mean** | **SD** | **Median** | **Minimum** | **Maximum** | **Q1** | **Q3** | **N** |
| **No** | 72.6 | 23.9 | 68.8 | 12.5 | 100.0 | 50.0 | 100.0 | 427 |
| **Yes** | 50.0 | 11.7 | 50.0 | 31.3 | 62.5 | 40.6 | 59.4 | 5 |
| **Total** | 72.3 | 23.9 | 68.8 | 12.5 | 100.0 | 50.0 | 100.0 | 432 |
| **p-value** | **<0.05 ^(*)^** | | | | | | | |

^(*)^ Mann-Whitney.

| **Did workplace have to be adapted?** | **Dimension 2 of TSQM: Adverse reactions** | | | | | | | |
| --- | --- | --- | --- | --- | --- | --- | --- | --- |
|  | **Mean** | **SD** | **Median** | **Minimum** | **Maximum** | **Q1** | **Q3** | **N** |
| **No** | 72.4 | 24.0 | 68.8 | 12.5 | 100.0 | 50.0 | 100.0 | 414 |
| **Yes** | 72.7 | 25.0 | 68.8 | 37.5 | 100.0 | 50.0 | 100.0 | 11 |
| **Total** | 72.4 | 24.0 | 68.8 | 12.5 | 100.0 | 50.0 | 100.0 | 425 |
| **p-value** | **0.972 ^(*)^** | | | | | | | |

^(*)^ Mann-Whitney.

| **Has he/she received rehabilitation?** | **Dimension 2 of TSQM: Adverse reactions** | | | | | | | |
| --- | --- | --- | --- | --- | --- | --- | --- | --- |
|  | **Mean** | **SD** | **Median** | **Minimum** | **Maximum** | **Q1** | **Q3** | **N** |
| **No** | 73.0 | 23.7 | 68.8 | 12.5 | 100.0 | 53.1 | 100.0 | 373 |
| **Yes** | 69.3 | 25.7 | 62.5 | 18.8 | 100.0 | 50.0 | 100.0 | 61 |
| **Total** | 72.5 | 23.9 | 68.8 | 12.5 | 100.0 | 50.0 | 100.0 | 434 |
| **p-value** | **0.289 ^(*)^** | | | | | | | |

^(*)^ Mann-Whitney.

| **Has he/she received informal care?** | **Dimension 2 of TSQM: Adverse reactions** | | | | | | | |
| --- | --- | --- | --- | --- | --- | --- | --- | --- |
|  | **Mean** | **SD** | **Median** | **Minimum** | **Maximum** | **Q1** | **Q3** | **N** |
| **No** | 72.8 | 23.9 | 68.8 | 12.5 | 100.0 | 50.0 | 100.0 | 408 |
| **Yes** | 66.5 | 24.6 | 62.5 | 25.0 | 100.0 | 50.0 | 93.8 | 25 |
| **Total** | 72.4 | 23.9 | 68.8 | 12.5 | 100.0 | 50.0 | 100.0 | 433 |
| **p-value** | **0.200 ^(*)^** | | | | | | | |

^(*)^ Mann-Whitney.

| **Number of pharmacological treatment received per patient for multiple sclerosis** | **Dimension 2 of TSQM: Adverse reactions** | | | | | | | |
| --- | --- | --- | --- | --- | --- | --- | --- | --- |
|  | **Mean** | **SD** | **Median** | **Minimum** | **Maximum** | **Q1** | **Q3** | **N** |
| **None** | 71.7 | 26.3 | 75.0 | 18.8 | 100.0 | 50.0 | 100.0 | 42 |
| **At least one** | 73.0 | 23.7 | 68.8 | 12.5 | 100.0 | 56.3 | 100.0 | 375 |
| **Total** | 72.9 | 24.0 | 68.8 | 12.5 | 100.0 | 53.1 | 100.0 | 417 |
| **p-value** | **0.789 ^(*)^** | | | | | | | |

^(*)^ Mann-Whitney.

### Dimension 3: Convenience

| **Number of visits to primary care for multiple sclerosis** | **Dimension 3 of TSQM: Convenience** | | | | | | | |
| --- | --- | --- | --- | --- | --- | --- | --- | --- |
|  | **Mean** | **SD** | **Median** | **Minimum** | **Maximum** | **Q1** | **Q3** | **N** |
| **None** | 63.0 | 18.3 | 66.7 | 0.0 | 100.0 | 50.0 | 75.0 | 202 |
| **At least one** | 60.5 | 18.4 | 66.7 | 16.7 | 100.0 | 50.0 | 66.7 | 83 |
| **Total** | 62.3 | 18.3 | 66.7 | 0.0 | 100.0 | 50.0 | 75.0 | 285 |
| **p-value** | **0.175 ^(*)^** | | | | | | | |

^(*)^ Mann-Whitney.

| **Number of visits to the hospital for multiple sclerosis** | **Dimension 3 of TSQM: Convenience** | | | | | | | |
| --- | --- | --- | --- | --- | --- | --- | --- | --- |
|  | **Mean** | **SD** | **Median** | **Minimum** | **Maximum** | **Q1** | **Q3** | **N** |
| **None** | 60.3 | 17.6 | 66.7 | 16.7 | 91.7 | 50.0 | 66.7 | 29 |
| **At least one** | 62.5 | 19.2 | 66.7 | 0.0 | 100.0 | 50.0 | 75.0 | 362 |
| **Total** | 62.4 | 19.0 | 66.7 | 0.0 | 100.0 | 50.0 | 75.0 | 391 |
| **p-value** | **0.558 ^(*)^** | | | | | | | |

^(*)^ Mann-Whitney.

| **Number of hospitalizations for multiple sclerosis** | **Dimension 3 of TSQM: Convenience** | | | | | | | |
| --- | --- | --- | --- | --- | --- | --- | --- | --- |
|  | **Mean** | **SD** | **Median** | **Minimum** | **Maximum** | **Q1** | **Q3** | **N** |
| **None** | 62.6 | 18.7 | 66.7 | 0.0 | 100.0 | 50.0 | 75.0 | 374 |
| **At least one** | 57.5 | 21.3 | 66.7 | 16.7 | 100.0 | 33.3 | 75.0 | 31 |
| **Total** | 62.2 | 18.9 | 66.7 | 0.0 | 100.0 | 50.0 | 75.0 | 405 |
| **p-value** | **0.186 ^(*)^** | | | | | | | |

^(*)^ Mann-Whitney.

| **Total number of tests/tests for multiple sclerosis** | **Dimension 3 of TSQM: Convenience** | | | | | | | |
| --- | --- | --- | --- | --- | --- | --- | --- | --- |
|  | **Mean** | **SD** | **Median** | **Minimum** | **Maximum** | **Q1** | **Q3** | **N** |
| **None** | 68.5 | 12.3 | 75.0 | 41.7 | 83.3 | 62.5 | 75.0 | 9 |
| **At least one** | 62.3 | 19.2 | 66.7 | 0.0 | 100.0 | 50.0 | 75.0 | 379 |
| **Total** | 62.5 | 19.1 | 66.7 | 0.0 | 100.0 | 50.0 | 75.0 | 388 |
| **p-value** | **0.241 ^(*)^** | | | | | | | |

^(*)^ Mann-Whitney.

| **Number of ambulance trips for multiple sclerosis** | **Dimension 3 of TSQM: Convenience** | | | | | | | |
| --- | --- | --- | --- | --- | --- | --- | --- | --- |
|  | **Mean** | **SD** | **Median** | **Minimum** | **Maximum** | **Q1** | **Q3** | **N** |
| **None** | 62.3 | 19.2 | 66.7 | 0.0 | 100.0 | 50.0 | 75.0 | 393 |
| **At least one** | 50.0 | .0 | 50.0 | 50.0 | 50.0 | 50.0 | 50.0 | 2 |
| **Total** | 62.2 | 19.1 | 66.7 | 0.0 | 100.0 | 50.0 | 75.0 | 395 |
| **p-value** | **0.236 ^(*)^** | | | | | | | |

^(*)^ Mann-Whitney.

| **Number of taxi trips for multiple sclerosis** | **Dimension 3 of TSQM: Convenience** | | | | | | | |
| --- | --- | --- | --- | --- | --- | --- | --- | --- |
|  | **Mean** | **SD** | **Median** | **Minimum** | **Maximum** | **Q1** | **Q3** | **N** |
| **None** | 62.4 | 19.2 | 66.7 | 0.0 | 100.0 | 50.0 | 75.0 | 371 |
| **At least one** | 54.2 | 11.5 | 54.2 | 41.7 | 66.7 | 41.7 | 66.7 | 6 |
| **Total** | 62.2 | 19.1 | 66.7 | 0.0 | 100.0 | 50.0 | 75.0 | 377 |
| **p-value** | **0.183 ^(*)^** | | | | | | | |

^(*)^ Mann-Whitney.

| **Did home have to be adapted?** | **Dimension 3 of TSQM: Convenience** | | | | | | | |
| --- | --- | --- | --- | --- | --- | --- | --- | --- |
|  | **Mean** | **SD** | **Median** | **Minimum** | **Maximum** | **Q1** | **Q3** | **N** |
| **No** | 62.3 | 19.3 | 66.7 | 0.0 | 100.0 | 50.0 | 75.0 | 420 |
| **Yes** | 57.8 | 19.4 | 58.3 | 33.3 | 100.0 | 41.7 | 66.7 | 17 |
| **Total** | 62.1 | 19.3 | 66.7 | 0.0 | 100.0 | 50.0 | 75.0 | 437 |
| **p-value** | **0.207 ^(*)^** | | | | | | | |

^(*)^ Mann-Whitney.

| **Did car have to be adapted?** | **Dimension 3 of TSQM: Convenience** | | | | | | | |
| --- | --- | --- | --- | --- | --- | --- | --- | --- |
|  | **Mean** | **SD** | **Median** | **Minimum** | **Maximum** | **Q1** | **Q3** | **N** |
| **No** | 62.3 | 19.3 | 66.7 | 0.0 | 100.0 | 50.0 | 75.0 | 431 |
| **Yes** | 53.3 | 17.3 | 50.0 | 33.3 | 75.0 | 37.5 | 70.8 | 5 |
| **Total** | 62.2 | 19.2 | 66.7 | 0.0 | 100.0 | 50.0 | 75.0 | 436 |
| **p-value** | **0.283 ^(*)^** | | | | | | | |

^(*)^ Mann-Whitney.

| **Did workplace have to be adapted?** | **Dimension 3 of TSQM: Convenience** | | | | | | | |
| --- | --- | --- | --- | --- | --- | --- | --- | --- |
|  | **Mean** | **SD** | **Median** | **Minimum** | **Maximum** | **Q1** | **Q3** | **N** |
| **No** | 62.3 | 19.1 | 66.7 | 0.0 | 100.0 | 50.0 | 75.0 | 418 |
| **Yes** | 56.1 | 27.7 | 58.3 | 0.0 | 100.0 | 41.7 | 66.7 | 11 |
| **Total** | 62.2 | 19.3 | 66.7 | 0.0 | 100.0 | 50.0 | 75.0 | 429 |
| **p-value** | **0.367 ^(*)^** | | | | | | | |

^(*)^ Mann-Whitney.

| **Has he/she received rehabilitation?** | **Dimension 3 of TSQM: Convenience** | | | | | | | |
| --- | --- | --- | --- | --- | --- | --- | --- | --- |
|  | **Mean** | **SD** | **Median** | **Minimum** | **Maximum** | **Q1** | **Q3** | **N** |
| **No** | 62.7 | 19.1 | 66.7 | 0.0 | 100.0 | 50.0 | 75.0 | 377 |
| **Yes** | 58.6 | 20.0 | 58.3 | 0.0 | 100.0 | 45.8 | 66.7 | 61 |
| **Total** | 62.1 | 19.2 | 66.7 | 0.0 | 100.0 | 50.0 | 75.0 | 438 |
| **p-value** | **0.096 ^(*)^** | | | | | | | |

^(*)^ Mann-Whitney.

| **Has he/she received informal care?** | **Dimension 3 of TSQM: Convenience** | | | | | | | |
| --- | --- | --- | --- | --- | --- | --- | --- | --- |
|  | **Mean** | **SD** | **Median** | **Minimum** | **Maximum** | **Q1** | **Q3** | **N** |
| **No** | 62.7 | 19.1 | 66.7 | 0.0 | 100.0 | 50.0 | 75.0 | 412 |
| **Yes** | 53.0 | 19.5 | 50.0 | 0.0 | 91.7 | 37.5 | 66.7 | 25 |
| **Total** | 62.1 | 19.3 | 66.7 | 0.0 | 100.0 | 50.0 | 75.0 | 437 |
| **p-value** | **<0.05 ^(*)^** | | | | | | | |

^(*)^ Mann-Whitney.

| **Number of pharmacological treatment received per patient for multiple sclerosis** | **Dimension 3 of TSQM: Convenience** | | | | | | | |
| --- | --- | --- | --- | --- | --- | --- | --- | --- |
|  | **Mean** | **SD** | **Median** | **Minimum** | **Maximum** | **Q1** | **Q3** | **N** |
| **None** | 64.3 | 18.7 | 66.7 | 8.3 | 100.0 | 50.0 | 77.1 | 42 |
| **At least one** | 62.1 | 18.9 | 66.7 | 0.0 | 100.0 | 50.0 | 75.0 | 379 |
| **Total** | 62.3 | 18.9 | 66.7 | 0.0 | 100.0 | 50.0 | 75.0 | 421 |
| **p-value** | **0.549 ^(*)^** | | | | | | | |

^(*)^ Mann-Whitney.

### Dimension 4: Overall satisfaction

| **Number of visits to primary care for multiple sclerosis** | **Dimension 4 of TSQM: Overall satisfaction** | | | | | | | |
| --- | --- | --- | --- | --- | --- | --- | --- | --- |
|  | **Mean** | **SD** | **Median** | **Minimum** | **Maximum** | **Q1** | **Q3** | **N** |
| **None** | 70.3 | 18.0 | 71.4 | 0.0 | 100.0 | 57.1 | 85.7 | 202 |
| **At least one** | 66.5 | 20.1 | 71.4 | 14.3 | 100.0 | 50.0 | 78.6 | 83 |
| **Total** | 69.2 | 18.7 | 71.4 | 0.0 | 100.0 | 57.1 | 78.6 | 285 |
| **p-value** | **0.095 ^(*)^** | | | | | | | |

^(*)^ Mann-Whitney.

| **Number of visits to the hospital for multiple sclerosis** | **Dimension 4 of TSQM: Overall satisfaction** | | | | | | | |
| --- | --- | --- | --- | --- | --- | --- | --- | --- |
|  | **Mean** | **SD** | **Median** | **Minimum** | **Maximum** | **Q1** | **Q3** | **N** |
| **None** | 72.4 | 15.8 | 71.4 | 50.0 | 100.0 | 57.1 | 82.1 | 29 |
| **At least one** | 68.6 | 18.7 | 71.4 | 0.0 | 100.0 | 57.1 | 78.6 | 362 |
| **Total** | 68.9 | 18.5 | 71.4 | 0.0 | 100.0 | 57.1 | 78.6 | 391 |
| **p-value** | **0.372 ^(*)^** | | | | | | | |

^(*)^ Mann-Whitney.

| **Number of hospitalizations for multiple sclerosis** | **Dimension 4 of TSQM: Overall satisfaction** | | | | | | | |
| --- | --- | --- | --- | --- | --- | --- | --- | --- |
|  | **Mean** | **SD** | **Median** | **Minimum** | **Maximum** | **Q1** | **Q3** | **N** |
| **None** | 68.7 | 19.1 | 71.4 | 0.0 | 100.0 | 57.1 | 78.6 | 374 |
| **At least one** | 66.8 | 16.4 | 71.4 | 28.6 | 85.7 | 57.1 | 78.6 | 31 |
| **Total** | 68.5 | 18.9 | 71.4 | 0.0 | 100.0 | 57.1 | 78.6 | 405 |
| **p-value** | **0.609 ^(*)^** | | | | | | | |

^(*)^ Mann-Whitney.

| **Total number of tests/tests for multiple sclerosis** | **Dimension 4 of TSQM: Overall satisfaction** | | | | | | | |
| --- | --- | --- | --- | --- | --- | --- | --- | --- |
|  | **Mean** | **SD** | **Median** | **Minimum** | **Maximum** | **Q1** | **Q3** | **N** |
| **None** | 65.9 | 17.4 | 71.4 | 35.7 | 92.9 | 53.6 | 75.0 | 9 |
| **At least one** | 68.7 | 18.8 | 71.4 | 0.0 | 100.0 | 57.1 | 78.6 | 379 |
| **Total** | 68.7 | 18.8 | 71.4 | 0.0 | 100.0 | 57.1 | 78.6 | 388 |
| **p-value** | **0.533 ^(*)^** | | | | | | | |

^(*)^ Mann-Whitney.

| **Number of ambulance trips for multiple sclerosis** | **Dimension 4 of TSQM: Overall satisfaction** | | | | | | | |
| --- | --- | --- | --- | --- | --- | --- | --- | --- |
|  | **Mean** | **SD** | **Median** | **Minimum** | **Maximum** | **Q1** | **Q3** | **N** |
| **None** | 68.4 | 18.7 | 71.4 | 0.0 | 100.0 | 57.1 | 78.6 | 393 |
| **At least one** | 64.3 | 30.3 | 64.3 | 42.9 | 85.7 | 42.9 | - | 2 |
| **Total** | 68.4 | 18.7 | 71.4 | 0.0 | 100.0 | 57.1 | 78.6 | 395 |
| **p-value** | **0.839 ^(*)^** | | | | | | | |

^(*)^ Mann-Whitney.

| **Number of taxi trips for multiple sclerosis** | **Dimension 4 of TSQM: Overall satisfaction** | | | | | | | |
| --- | --- | --- | --- | --- | --- | --- | --- | --- |
|  | **Mean** | **SD** | **Median** | **Minimum** | **Maximum** | **Q1** | **Q3** | **N** |
| **None** | 68.7 | 18.7 | 71.4 | 0.0 | 100.0 | 57.1 | 78.6 | 371 |
| **At least one** | 57.1 | 22.1 | 57.1 | 21.4 | 85.7 | 42.9 | 75.0 | 6 |
| **Total** | 68.5 | 18.8 | 71.4 | 0.0 | 100.0 | 57.1 | 78.6 | 377 |
| **p-value** | **0.163 ^(*)^** | | | | | | | |

^(*)^ Mann-Whitney.

| **Did home have to be adapted?** | **Dimension 4 of TSQM: Overall satisfaction** | | | | | | | |
| --- | --- | --- | --- | --- | --- | --- | --- | --- |
|  | **Mean** | **SD** | **Median** | **Minimum** | **Maximum** | **Q1** | **Q3** | **N** |
| **No** | 68.7 | 18.5 | 71.4 | 0.0 | 100.0 | 57.1 | 78.6 | 421 |
| **Yes** | 65.5 | 21.3 | 71.4 | 14.3 | 100.0 | 50.0 | 82.1 | 17 |
| **Total** | 68.6 | 18.6 | 71.4 | 0.0 | 100.0 | 57.1 | 78.6 | 438 |
| **p-value** | **0.497 ^(*)^** | | | | | | | |

^(*)^ Mann-Whitney.

| **Did car have to be adapted?** | **Dimension 4 of TSQM: Overall satisfaction** | | | | | | | |
| --- | --- | --- | --- | --- | --- | --- | --- | --- |
|  | **Mean** | **SD** | **Median** | **Minimum** | **Maximum** | **Q1** | **Q3** | **N** |
| **No** | 68.8 | 18.5 | 71.4 | 0.0 | 100.0 | 57.1 | 78.6 | 432 |
| **Yes** | 51.4 | 23.9 | 57.1 | 14.3 | 71.4 | 28.6 | 71.4 | 5 |
| **Total** | 68.6 | 18.6 | 71.4 | 0.0 | 100.0 | 57.1 | 78.6 | 437 |
| **p-value** | **0.073 ^(*)^** | | | | | | | |

^(*)^ Mann-Whitney.

| **Did workplace have to be adapted?** | **Dimension 4 of TSQM: Overall satisfaction** | | | | | | | |
| --- | --- | --- | --- | --- | --- | --- | --- | --- |
|  | **Mean** | **SD** | **Median** | **Minimum** | **Maximum** | **Q1** | **Q3** | **N** |
| **No** | 68.7 | 18.8 | 71.4 | 0.0 | 100.0 | 57.1 | 78.6 | 419 |
| **Yes** | 67.9 | 14.6 | 71.4 | 50.0 | 92.9 | 57.1 | 78.6 | 11 |
| **Total** | 68.6 | 18.7 | 71.4 | 0.0 | 100.0 | 57.1 | 78.6 | 430 |
| **p-value** | **0.706 ^(*)^** | | | | | | | |

^(*)^ Mann-Whitney.

| **Has he/she received rehabilitation?** | **Dimension 4 of TSQM: Overall satisfaction** | | | | | | | |
| --- | --- | --- | --- | --- | --- | --- | --- | --- |
|  | **Mean** | **SD** | **Median** | **Minimum** | **Maximum** | **Q1** | **Q3** | **N** |
| **No** | 69.9 | 18.1 | 71.4 | 0.0 | 100.0 | 57.1 | 78.6 | 377 |
| **Yes** | 61.2 | 20.3 | 67.9 | 0.0 | 92.9 | 42.9 | 78.6 | 62 |
| **Total** | 68.7 | 18.6 | 71.4 | 0.0 | 100.0 | 57.1 | 78.6 | 439 |
| **p-value** | **<0.005 ^(*)^** | | | | | | | |

^(*)^ Mann-Whitney.

| **Has he/she received informal care?** | **Dimension 4 of TSQM: Overall satisfaction** | | | | | | | |
| --- | --- | --- | --- | --- | --- | --- | --- | --- |
|  | **Mean** | **SD** | **Median** | **Minimum** | **Maximum** | **Q1** | **Q3** | **N** |
| **No** | 69.0 | 18.4 | 71.4 | 0.0 | 100.0 | 57.1 | 78.6 | 413 |
| **Yes** | 62.4 | 21.5 | 71.4 | 14.3 | 92.9 | 42.9 | 78.6 | 25 |
| **Total** | 68.6 | 18.6 | 71.4 | 0.0 | 100.0 | 57.1 | 78.6 | 438 |
| **p-value** | **0.168 ^(*)^** | | | | | | | |

^(*)^ Mann-Whitney.

| **Number of pharmacological treatment received per patient for multiple sclerosis** | **Dimension 4 of TSQM: Overall satisfaction** | | | | | | | |
| --- | --- | --- | --- | --- | --- | --- | --- | --- |
|  | **Mean** | **SD** | **Median** | **Minimum** | **Maximum** | **Q1** | **Q3** | **N** |
| **None** | 70.9 | 20.3 | 71.4 | 14.3 | 100.0 | 57.1 | 85.7 | 42 |
| **At least one** | 68.5 | 18.5 | 71.4 | 0.0 | 100.0 | 57.1 | 78.6 | 380 |
| **Total** | 68.8 | 18.7 | 71.4 | 0.0 | 100.0 | 57.1 | 78.6 | 422 |
| **p-value** | **0.290 ^(*)^** | | | | | | | |

^(*)^ Mann-Whitney.
